# Unlocking the Potential of Carbon Nitride (C*_x_* N*_y_*) Electrocatalysts in Hydrogen Evolution, Oxygen Evolution and Overall Water Splitting

**DOI:** 10.1002/EXP.20240008

**Published:** 2026-02-11

**Authors:** Zi Wei Tan, Neil Felicio Agesta, Ryan Yow Zhong Yeo, Joel Jie Foo, Wee‐Jun Ong

**Affiliations:** ^1^ School of Energy and Chemical Engineering Xiamen University Malaysia Selangor Darul Ehsan Malaysia; ^2^ Center of Excellence for NaNo Energy & Catalysis Technology (CONNECT) Xiamen University Malaysia Selangor Darul Ehsan Malaysia; ^3^ State Key Laboratory of Physical Chemistry of Solid Surfaces College of Chemistry and Chemical Engineering Xiamen University Xiamen China; ^4^ Gulei Innovation Institute Xiamen University Zhangzhou China; ^5^ Shenzhen Research Institute of Xiamen University Shenzhen China; ^6^ Department of Chemical and Biological Engineering College of Engineering Korea University Seoul Republic of Korea

**Keywords:** carbon nitride, electrocatalysis, water splitting

## Abstract

Electrocatalytic water splitting with carbon nitride (C*_x_* N*_y_*) materials has gained significant interest, driven by extensive research validating their synthesis, structure, and applications. Among various C*_x_* N*_y_* stoichiometric ratios, graphitic carbon nitride (g‐C_3_N_4_) stands out as the most stable configuration and is extensively studied in the literature. However, other C*_x_* N*_y_* structures like g‐CN, C_2_N, C_3_N_5_, and C_9_N_4_ have been booming recently as well by exploration of their greatness. Recent progress of C*_x_* N*_y_*‐based electrocatalysts in hydrogen evolution reaction (HER), oxygen evolution reaction (OER), and overall water splitting (OWS) has been examined meticulously. More emphasis has been placed on exploring the synthesis‐structure‐performance and simulate‐structure‐performance relationships of C*_x_* N*_y_* electrocatalysts in experimental and computational studies, respectively. This review outlines a clear framework for unifunctional C*_x_* N*_y_* electrocatalysts in HER and OER, focusing on recent advancements in (1) defect engineering, (2) structural engineering, and (3) hybridization. The research on bifunctional C*_x_* N*_y_* electrocatalysts is highlighted and explored through two main approaches: intrinsic and extrinsic modifications. Finally, the strategies and perspectives for creating novel highly efficient C*_x_* N*_y_* electrocatalysts for HER, OER, and OWS are analyzed. This review aims to inspire researchers to incorporate computational methods into experimental studies for developing highly efficient bifunctional C*_x_* N*_y_*‐based electrocatalysts.

## Graphical abstract

Electrocatalytic water splitting with carbon nitride (C*_x_* N*_y_*) materials has gained attention due to their synthesis, structure, and robust application. Among various C*_x_* N*_y_* types, g‐C_3_N_4_ is the most stable allotrope and extensively researched. This review explores recent advances in HER, OER, and OWS, focusing on defect engineering, structural engineering, hybridization, and the potential of bifunctional electrocatalysts through intrinsic and extrinsic modifications.

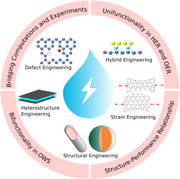

## 1 Introduction

The emergence of hydrogen (H_2_) energy as an alternative to fossil fuels is believed to be the key resolution towards a sustainable and carbon neutral future [1, 2], as well as addressing conundrums associated with energy security faced by nations globally [3]. Global energy consumption increased steadily since the beginning of the twentieth century (Figure 2A), with a slight dip in the year 2020 due to the COVID‐19 pandemic where transportation fuels experienced the greatest reduction during the strict lockdowns [4, 5]. However, the recovery of industries in the post‐pandemic era will surely drive up the energy consumption trends on forthcoming years. In 2020, non‐renewable sources namely oil, coal, and gas had represented 80% of the world's energy consumption, while electricity and biomass as renewable energy merely contributed 20% (Figure 2B). In spite of that, the aforementioned energy breakdown ratio in terms of renewable energy will expect a rise in the future owing to H_2_’s exceptional electricity storage capabilities [6]. In November 2021, United Nations Framework Convention on Climate Change (UNFCCC) COP26 was held at Glasgow and highlighted that renewable and low carbon containing H_2_ will be available globally by 2030 [7]. As such, the synthesis and utilization of H_2_ have been pronounced as a promising pathway to concurrently curb greenhouse gases emissions by fossil fuels and generate clean electricity to achieve carbon neutrality [8, 9, 10]. In view of the importance of H_2_ generation in large‐scale, efficient electrochemical overall water splitting (OWS) can be achieved with the aid of electrocatalysts [11, 12]. The usage of electrocatalysts has proven the necessity to overcome a major bottleneck of electrochemical OWS, which is large overpotentials of water electroreduction (H_2_O→H_2_) and electrooxidation (H_2_O→O_2_) reaction by lowering the activation energy and improving the efficiency of OWS [13, 14, 15, 16]. Therefore, exploration of excellent electrocatalysts for this matter will ensure a rewarding direction towards a clean future.

Electrocatalysts for water splitting are broadly categorized into noble metal‐based, transition metal‐based, and metal‐free catalysts, each with distinct advantages and challenges. Noble metal‐based catalysts, such as platinum (Pt), ruthenium oxide (RuO_2_) or iridium oxide (IrO_2_), exhibit superior catalytic activity and stability but suffer from high cost and scarcity. Pt is recognized as one of the most effective catalysts for HER [17, 18] while IrO_2_ and RuO_2_ are excellent catalysts for OER in acidic media [19, 20, 21, 22]. Transition metal‐based catalysts, including sulfides (e.g., MoS_2_, CoS_2_) [23, 24, 25], phosphides (e.g., Ni_2_P, CoP) [26, 27, 28, 29, 30], oxides (e.g., Co_3_O_4_, Fe_2_O_3_) [31, 32, 33, 34], and hydroxide (e.g., Ni(OH)_2_) [35, 36, 37, 38], have gained attention as cost‐effective alternatives with tunable electronic structures and abundant active sites. These materials leverage earth‐abundant elements such as nickel (Ni), cobalt (Co), molybdenum (Mo), and iron (Fe), which exhibit excellent catalytic performance through synergistic charge transfer mechanisms.

Carbon‐based metal‐free catalysts have emerged as promising alternatives to traditional metal‐based electrocatalysts due to their abundance, environmental friendliness, chemical stability, and tunable electronic properties. Unlike noble metal catalysts, carbon materials offer cost‐effective and sustainable solutions for various electrocatalytic reactions. The exceptional electrical conductivity, high surface area, and tunable surface chemistry of carbon‐based materials contribute significantly to their catalytic performance [39, 40]. Carbon dots (CDs), on the other hand, leverage their quantum confinement effects and abundant functional groups for enhanced charge transfer and reaction kinetics in electrocatalytic reaction [41, 42, 43, 44, 45, 46, 47]. Carbon nanotubes (CNTs) provide excellent mechanical strength and electrical conductivity, with their tubular morphology offering unique advantages for mass transport and active site exposure [48, 49]. Graphene and its derivatives (e.g., reduced graphene oxide, doped graphene) exhibit high electron mobility and conductivity, making them ideal for charge transfer in electrocatalysis [50, 51, 52, 53]. However, their pristine form lacks active sites, necessitating modifications such as heteroatom doping (N, B, S, P) [54] or structural engineering [55] to enhance their catalytic activity. Table 1 summarizes structural and properties, electrocatalytic application and comparison of C*_x_* N*_y_* with other carbon allotropes.

**TABLE 1:** Summary of structure and properties, electrocatalytic application, and comparison of C*_x_* N*_y_* with other carbon allotropes.

Material	Structure and properties	Electrocatalytic applications	Comparison with carbon nitride (C*x*N*y*)	Ref.
**Carbon dots (CDs)**	Quantum‐sized particles (<10 nm), tunable photoluminescence, excellent solubility	Electrochemical sensor, AOR, ORR, OER, HER, and CO_2_RR	CDs have high surface area and fluorescence, but C*_x_* N*_y_* offers better stability and catalytic activity in HER/OER	[41, 42, 43, 44, 45, 46, 47, 56]
**Carbon nanotubes (CNTs)**	Cylindrical tubes, high conductivity, mechanical strength, moderate surface area (100–400 m^2^ g^−1^)	Fuel cell, electrochemical sensor, capacitor, ORR, OER, HER, NRR, and CO_2_RR	CNTs offer superior electrical conductivity, but C*_x_* N*_y_* has better metal‐support interactions for catalysis	[48, 49, 57]
**Graphene**	Single‐layer sp^2^ hybridized carbon, high conductivity, large surface area	Fuel cell, DSC, batteries, electrochemical sensor, AOR, ORR, OER, HER, NRR, and CO_2_RR	Graphene is conductive but suffers from sheet restacking; C*_x_* N*_y_* has intrinsic catalytic sites	[50, 51, 58, 59]

*Note*: DSC refer to dye sensitized solar cell, AOR refer to alcohol oxidation, ORR refer to oxygen oxidation reaction, OER refer to oxygen evolution reaction, HER refer to hydrogen evolution reaction, NRR refer to nitrogen reduction reaction and CO_2_RR refer to carbon dioxide reduction reaction.

**FIGURE 1:**
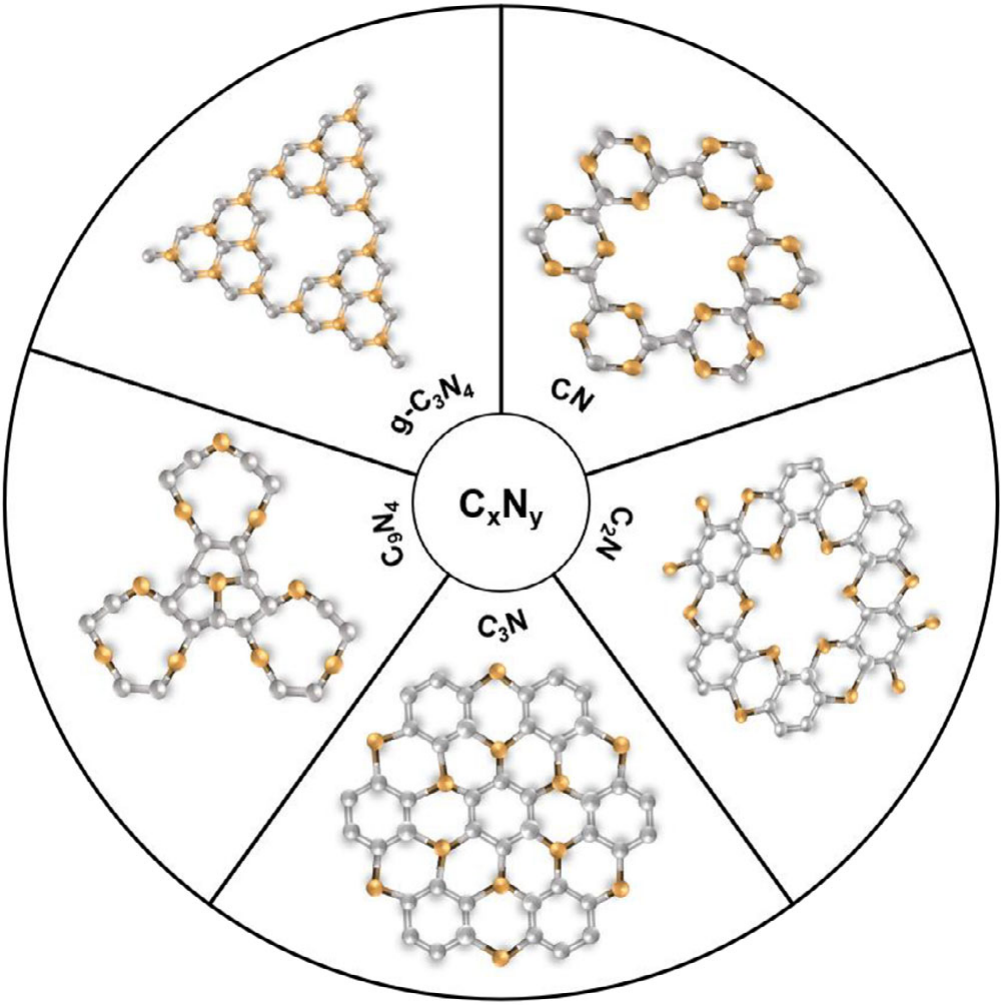
C*_x_* N*_y_* with different stoichiometric ratios for electrocatalytic OWS.

Among existing electrocatalysts, carbon nitride (C*_x_* N*_y_*) is an exceptional candidate to serve for this purpose. Generally, C*_x_* N*_y_* is a metal‐free material consisting of two earth‐abundant elements mainly carbon (*x*) and nitrogen (*y*) [60], which can be easily synthesized through thermal polymerization at low cost from a diversity of nitrogen‐rich precursors [61, 62]. Furthermore, C*_x_* N*_y_* materials have been garnering attention in electrocatalyst fabrication due to its low toxicity [63, 64], low cost [65], high thermal and chemical stability [66, 67], which serves as a viable alternative to expensive noble metals. Considering the aforementioned characteristics of virtue, C*_x_* N*_y_*‐based electrocatalysts have been widely studied in different electrocatalytic operations, such as overall water splitting [68, 69, 70], carbon dioxide reduction [71, 72], oxygen reduction [73, 74], nitrogen fixation [75, 76, 77], and degradation of organic pollutants [78, 79]. Noticeably, graphitic carbon nitride (g‐C_3_N_4_) is the most extensively researched C*_x_* N*_y_* material among its peers in this topic. Starting with its structure, g‐C_3_N_4_ is constructed of thermodynamically stable 2D tri‐*s*‐triazine ring structure with conjugated π‐bonds [80, 81, 82, 83], which is formed between sp^2^‐hybridization of carbon and nitrogen atoms [84]. Additionally, these nitrogen atoms on g‐C_3_N_4_ and other C*_x_* N*_y_* frameworks have lone pair electrons acting as Lewis base sites, which allow the incorporation of metal atoms [85, 86], and further improve the electrocatalytic conversion reactions. Next, the polymeric nature of C*_x_* N*_y_* materials also contributes to its flexibility and surface tunability, granting it the capability to anchor inorganic nanoparticles as a host matrix [87, 88]. In short, C*_x_* N*_y_* materials and their intrinsic characteristics open up many new avenues for researchers to perform experiments with it, rendering them such a popular material recently. Beyond graphitic carbon nitride (g‐C_3_N_4_) electrocatalysts, C*_x_* N*_y_* with different stoichiometric ratios between carbon and nitrogen have also been discovered and documented, which include g‐CN, C_2_N, C_2_N_2_, C_2_N_3_, C_3_N, C_3_N_5_, C_3_N_6_, C_4_N, C_4_N_2_, C_5_N, C_5_N_2_, and C_9_N_4_ [89, 90, 91, 92]_._ Nevertheless, only a number of C*_x_* N*_y_* materials (g‐C_3_N_4_ [93, 94], CN [95], C_2_N [91, 96], C_3_N [97], and C_9_N_4_ [98, 99]) are studied previously in the context of electrocatalytic OWS due to their bifunctionality (Figure 1).

**FIGURE 2:**
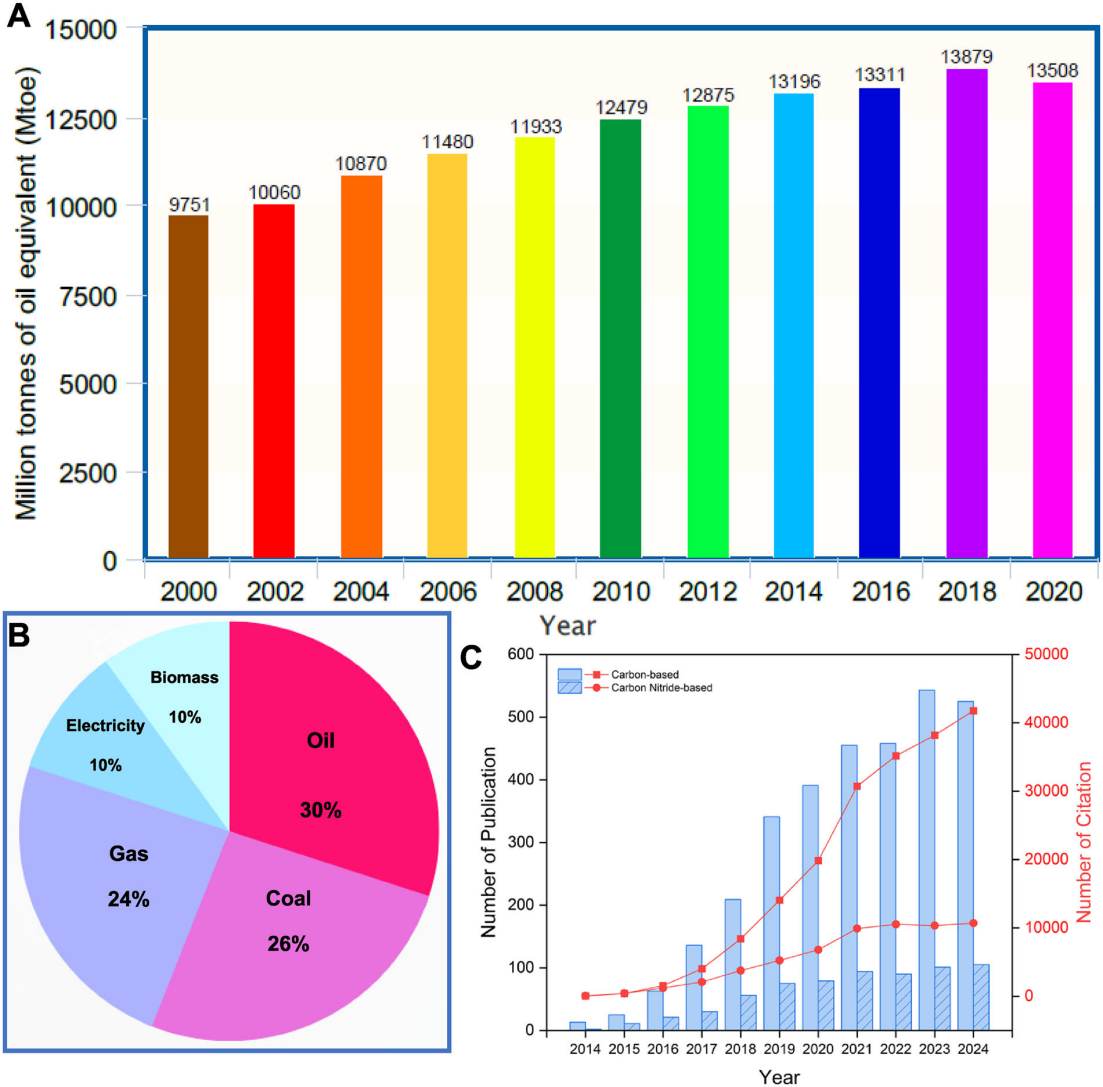
(A) Global energy consumption from year 2000 to 2020 [102]. (B) Energy consumption breakdown of year 2020 [102]. (C) Annual publications and citations from 2012 to 2024 on carbon‐based and carbon nitride‐based electrocatalysts for water splitting, sourced from the Web of Science database, accessed December 31, 2024.

In contrast to bifunctional OWS electrocatalysts, there are also various unifunctional C*_x_* N*_y_* electrocatalysts reported previously that showed exceptional performances in either one of the OWS half reactions, which is hydrogen evolution reaction (HER) and oxygen evolution reaction (OER). From the perspective of renewable energy development, OER shares similar significance with its OWS half reaction counterpart, especially in the promising metal–air battery technologies [100]. Over the past decade, an increasing number of articles published on carbon‐based and carbon nitride‐based electrocatalysts for OWS can be observed in Figure 2c. Up‐to‐date, C*_x_* N*_y_* was included in various review works as a highlight among other catalysts in electrocatalytic processes [101]. In addition to that, review articles associating C*_x_* N*_y_*‐based electrocatalysts with HER, OER, and OWS were usually published separately based on their individual domains and not collectively within a single work. Therefore, a timely review on C*_x_* N*_y_*‐based catalysts in electrocatalytic HER, OER, and OWS of such merit is immensely desirable for this rapidly growing field.

**FIGURE 3:**
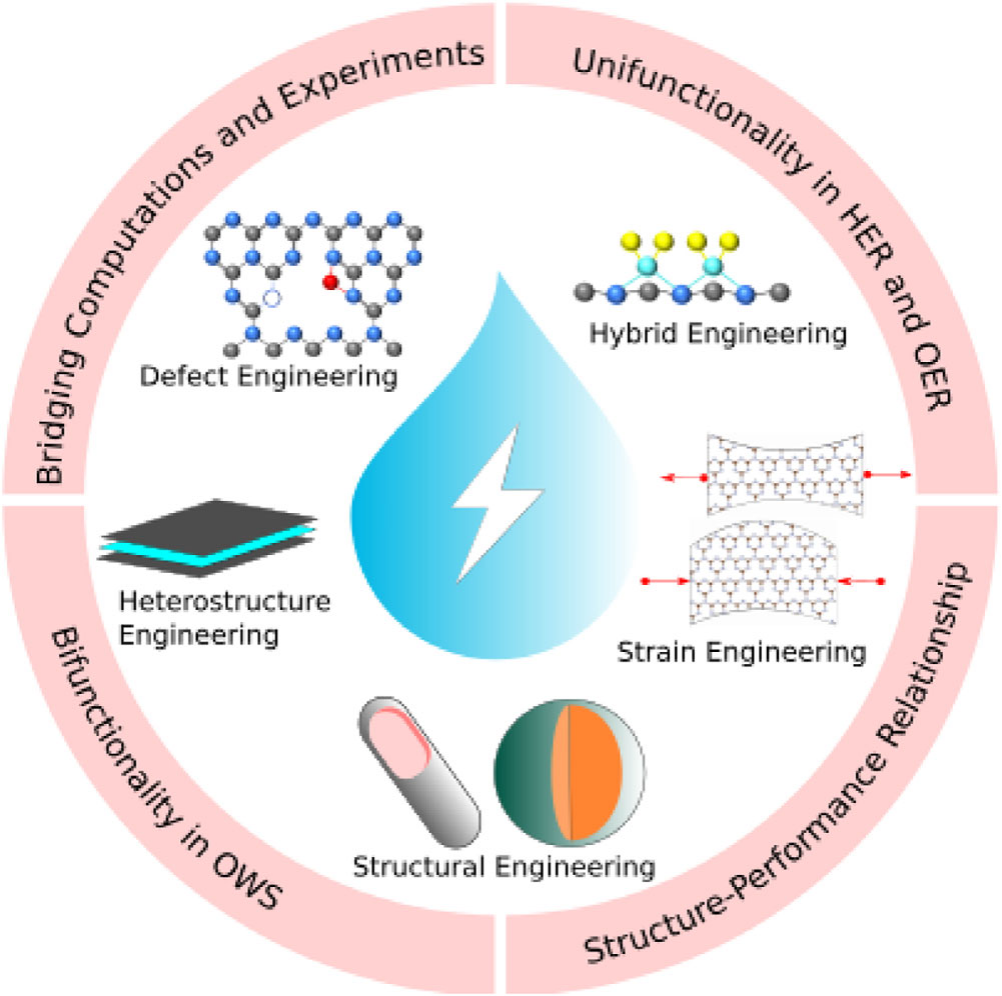
Investigation of structure‐performance relationship on the modification techniques of C*_x_* N*_y_* via computations and experiments for unifunctional HER/OER and bifunctional OWS.

With an aim to provide a comprehensive account of C*_x_* N*_y_* electrocatalysts, we introduce the topic with an overview of OWS and its half reactions: HER and OER, followed by the evaluation criteria for electrocatalytic activities. With the aid of computational simulations and experimental outcome, numerous unifunctional and bifunctional C*_x_* N*_y_*‐based electrocatalysts are highlighted. Most importantly, the structure‐performance relationship of each work is clearly disclosed and comparative studies with state‐of‐the‐art electrocatalysts are also discussed (Figure 3). Furthermore, new and exciting applications of these electrocatalysts such as seawater splitting will be discussed. Lastly, future prospects in this frontier are presented based on catalytic environment, self‐standing catalysis, catalyst and system design, and machine learning.

## 2 Principles of Electrocatalytic Water Splitting

### 2.1 Hydrogen Evolution Reaction (HER) Mechanism

In general, electrocatalytic HER is a water reduction reaction, which is influenced by the pH value of the electrolyte. Desired product H_2_ can be generated by reducing proton (H^+^) in acidic media or H_2_O in alkaline media through a series of elementary steps [103, 104, 105]. HER in acidic media occurs via two possible pathways: Volmer–Heyrovsky and Volmer–Tafel. In the V‐H pathway, HER begins with the adsorption of H^+^ onto the catalyst's surface (Equation 1) yielding H_ads_, followed by the combination of another H^+^ with H_ads_ to form H_2_ and further desorbed from the electrocatalyst surface (Equation 2). Meanwhile, Volmer–Tafel route follows a similar adsorption method, but the H_2_ is formed by the combination of two H_ads_ (Equation 3).

(1) $$\left(i\right)\;\text{Volmer step}\;\left(V\right)\;\;\;:H^{+}+e^{-}\to H_{\mathrm{ads}}$$

(2) $$\left(\mathrm{ii}\right)\;\text{Heyrovsky step}\;\left(H\right)\;\;\;:H^{+}+e^{-}+H_{\mathrm{ads}}\to H_{2}$$

(3) $$\left(\mathrm{iii}\right)\;\text{Tafel step}\;\;\left(T\right)\;\;\;:2H_{\mathrm{ads}}\to H_{2}$$

where H_ads_ represents the hydrogen adsorbed on the catalyst active sites.

Similarly, HER in alkaline media also proceeds via Volmer–Heyrovsky and Volmer–Tafel pathways. However, prior to the adsorption step, HER in alkaline media begins with the dissociation of H_2_O molecules to provide H^+^ for the adsorption process (Equation 4), as well as H^+^ for the combination with H_ads_ to produce H_2_ (Equation 5). For Volmer–Tafel pathway in alkaline media, the Tafel step remains the same as of acidic conditions (Equation 3).

(4) $$\left(i\right)\;\text{Volmer step}\;\left(V\right)\;\;\;:H_{2}O+e^{-}\to OH^{-}+H_{\mathrm{ads}}$$

(5) $$\left(\mathrm{ii}\right)\;\text{Heyrovsky step}\;\left(H\right)\;\;\;\;:H_{2}O+e^{-}+H_{\mathrm{ads}}\to OH^{-}+H_{2}$$

### 2.2 Oxygen Evolution Reaction (OER) Mechanism

On the other hand, electrocatalytic OER is a water oxidation reaction involving four cooperative proton‐coupled electron transfer (PCET) steps, which limits the performance of the overall electrocatalytic process of water splitting [106]. Despite the sluggish OER kinetics, it is still considered as an essential anodic half reaction for other electrochemical processes, such as HER, carbon dioxide reduction reaction (CO_2_RR), and nitrogen reduction reaction (N_2_RR) [107]. Furthermore, the initiation of the PCET mechanism of OER is greatly influenced by the pH value of the reaction medium [108]. When an acidic media is used, H_2_O molecules are first adsorbed on the catalytic active site, undergoing oxidation process that releases an electron and leads to the formation of OH_ads_ and byproduct H^+^ (Equation 6), followed by another oxidation step to obtain O_ads_ (Equation 7). Next, the intermediate O_ads_ reacts with another H_2_O molecule to form OOH_ads_ through O─O bond formation (Equation 8). Lastly, OOH_ads_ is oxidized to release O_2_ from the catalyst surface and the active site is regenerated (Equation 9).

OER mechanism in acidic conditions, [109, 110]

(6) $$H_{2}O\left(l\right)\to OH_{\mathrm{ads}}+H^{+}+e^{-}$$

(7) $$OH_{\mathrm{ads}}\to O_{\mathrm{ads}}+H^{+}+e^{-}$$

(8) $$H_{2}O\left(l\right)+O_{\mathrm{ads}}\to \mathrm{OO}H_{\mathrm{ads}}+H^{+}+e^{-}$$

(9) $$\mathrm{OO}H_{\mathrm{ads}}\to O_{2}\left(g\right)+H^{+}+e^{-}$$

where (*l*) and (*g*) refer to liquid and gas phase, OH_ads_, O_ads_, and OOH_ads_ represent the intermediate species adsorbed on the catalytic active site.

In contrast, the presence of hydroxide ions (OH^−^) in alkaline media initiates the OER with an adsorption step of OH^−^ onto the active site giving rise to OH_ads_ (Equation 10). Due to the abundance of OH^−^ present in the medium, a consecutive reaction with OH^−^ leads to the formation of intermediate O_ads_ and OOH_ads_, byproduct H_2_O molecule, and the desired O_2_ (Equations (11), (12), (13)).

OER mechanism in alkaline conditions, [109, 111]

(10) $$OH^{-}\to OH_{\mathrm{ads}}+e^{-}$$

(11) $$OH_{\mathrm{ads}}+OH^{-}\to O_{\mathrm{ads}}+H_{2}O\left(l\right)+e^{-}$$

(12) $$O_{\mathrm{ads}}+OH^{-}\to \mathrm{OO}H_{\mathrm{ads}}+e^{-}$$

(13) $$\mathrm{OO}H_{\mathrm{ads}}+OH^{-}\to O_{2}\left(g\right)+H_{2}O\left(l\right)+e^{-}$$

In short, OER mechanisms in acidic and alkali media both releases four electrons for the evolution of a single O_2_ molecule. The overall OER equations in both acidic and alkaline conditions can be summarized as below: [21]

(14) $$\text{Acidic conditions}:2H_{2}O\left(l\right)\to O_{2}\left(g\right)+4H^{+}+4e^{-}$$

(15) $$\text{Alkaline conditions}:4OH^{-}\to O_{2}\left(g\right)+2H_{2}O\left(l\right)+4e^{-}$$

### 2.3 Properties of Graphitic Carbon Nitride as HER and OER Electrocatalysts

As mentioned before, thermodynamic stability of g‐C_3_N_4_ comes from its tri‐*s*‐triazine ring, allowing it to possess superb thermal stability. Zhu et al. conducted thermogravimetric analysis (TGA) measurements on g‐C_3_N_4_, demonstrating that this material remains stable up to 600°C in different environments (N_2_, O_2_, and atmospheric conditions), indicating its capability to withstand high temperatures and perform reliably in HER and OER at room temperature [112]. However, g‐C_3_N_4_ will not be able to withstand high temperature water electrolysis (700–1000°C) in which steam is dissociated into H_2_ and O_2_ instead of water, as g‐C_3_N_4_ will decompose beyond 600°C [112, 113].

Another unique property of g‐C_3_N_4_ materials is their inertness towards molecular O_2_, making them well suited for electrocatalytic OER [112], this inertness also supports the initial claim of its stability in oxidative environment. This property is significantly important as the subsequent reaction between g‐C_3_N_4_ and O_2_ after the OER might lead to generation of undesired products, which further dampens the overall yield.

A computational study by Afshan Mohajeri et al. identified CN as the most effective bifunctional electrocatalyst among C*_x_* N*_y_*, exhibiting remarkable performance for both OER and HER [114]. The results indicate that C_2_N, CN, and C_3_N_4_ exhibit greater charge transfer and higher adsorption energies compared to C_4_N and C_3_N suggesting stronger interactions between water molecules and the catalyst. The observed trend further reveals that increasing nitrogen content enhances intermediate adsorption, which favors the water splitting reaction. Adsorption energies generally decrease with higher nitrogen content due to charge density accumulation on the surface, reducing the tendency to adsorb negatively charged species. Moreover, a higher nitrogen content provides more active sites for hydrogen adsorption, while the increased electron density around the central cavities in C_2_N, CN, and C_3_N_4_ further facilitates hydrogen adsorption. In general, the catalytic performance of C*_x_* N*_y_* materials is strongly influenced by the C/N ratio, where a higher nitrogen content enhances intermediate adsorption and charge transfer, while a more balanced ratio optimizes active site availability and structural stability for efficient HER and OER.

### 2.4 Overview and Mechanism of OWS

**FIGURE 4:**
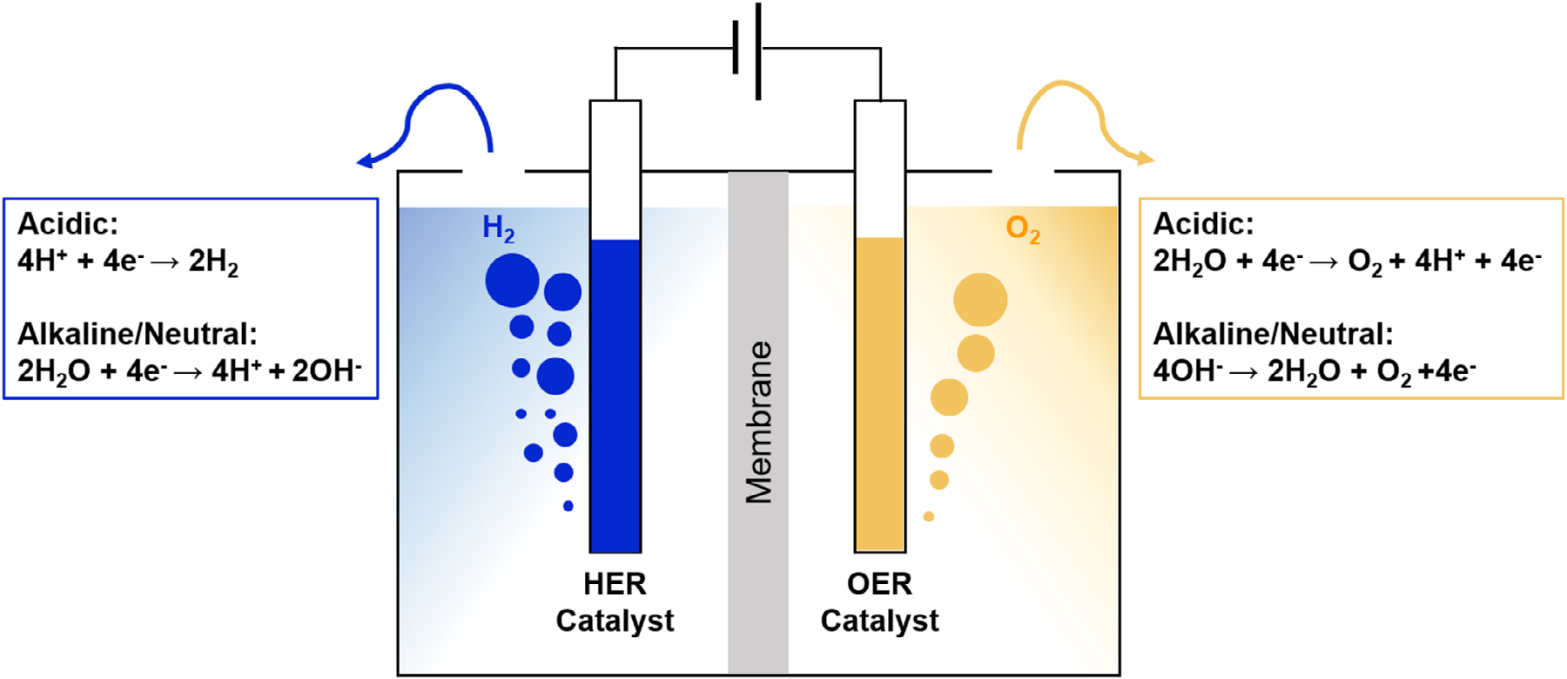
Schematic of electrochemical water splitting under different conditions.

Electrocatalytic OWS is an electrochemical setup consisting of HER (cathodic reaction) and OER (anodic reaction) occurring simultaneously within the same electrolyte, with H_2_O being the sole raw material. At the cathode, H^+^ undergoes reduction reaction to generate H_2_, while OH^−^ undergo oxidation reaction to generate O_2_ at the anode. Figure 4 shows the mechanism of OWS under various conditions. The OWS equation can be described as follows:

(16) $$H_{2}O\left(l\right)\to H_{2}\left(g\right)+\frac{1}{2}O_{2}\left(g\right)$$

Theoretically, the standard Gibbs free energy change (∆*G*°) of water splitting is 237 kJ/mol and its thermodynamic potential is 1.23 V under standard conditions of 1 atm and 25°C [115, 116]. However, the voltage applied in real scenario will always exceed the theoretical values due to overpotential and uncompensated resistances of the system, which will be discussed further in the next subsection. Based on Xiang et al., water electrolysis systems with low overpotentials (0.7–1.0 V) and resistances (0.5 V) can exhibit up to 50% electrical‐to‐chemical conversion efficiency in H_2_ and O_2_ production [117].

As we all know, there are two distinguished methods that energy can be supplied to drive the onset of OWS process. Photocatalysis relies on visible light irradiation in which the catalyst's behavior operates based on the band theory of semiconductors [118, 119]; while electrocatalysis receives its energy from an externally supplied electrical current. Hence, the operating principle of electrocatalytic OWS largely relies on the electrical conductivity of the cell. Apart from the electrodes’ electrical conductivity, a low resistance conducting wire connecting the electrodes and power source externally should also be used to improve electrical conductivity and prevent voltage loss [120, 121]. Conclusively, electrical conductivity of the cell internally and externally greatly affects the electrocatalytic OWS process, which should be improved to ensure a high efficiency process.

The principles of OWS also extend towards its electrocatalysts design. HER and OER electrocatalysts are highly competitive in their respective acidic and alkaline mediums, but a common complication arises when they are integrated jointly during OWS as the catalysts are usually synthesized under different conditions [101, 122]. For instance, HER electrocatalysts produce H_2_ in abundance but show poor OER performance during OWS process when an acidic electrolyte is used and vice versa. Furthermore, the aforementioned PCET steps of OER consisting of O‐H bonds breaking and O─O bonds formation are kinetically sluggish and lead to a higher overpotential of the OWS process [123, 124]. Thus, electrocatalysts design is constantly being one of the focal points in the field of catalysis. Huang et al. highlighted two principles in designing electrocatalysts for the application of industrial water electrolysis: (1) electrocatalysts possess high catalytic activity that can produce large current densities with minimum overpotential and (2) electrocatalysts have high stability that can undergo multiple cycles of water splitting with sustained performance [125]. Up to date, prevalent modification strategies were devised to develop electrocatalysts with high catalytic activity, which include defect engineering [126, 127, 128], surface and interface engineering [129, 130, 131], heterostructure engineering [132, 133], electronic and structural engineering, [134, 135, 136] and hybrids construction [137, 138, 139].

### 2.5 Evaluation Criteria of Electrocatalytic HER, OER, and OWS

With the rapid growth of the electrocatalysis field, a range of standardized criteria is used to evaluate the electrocatalytic performances of the synthesized catalyst in their respective processes. The key evaluation criteria discussed in this subsection include overpotential (*η*), exchange current density (*j*_0_), Tafel slopes, turnover frequency (TOF), electrochemically active surface area (ECSA), as well as catalyst stability.

#### 2.5.1 Overpotential (*η*)

Overpotential, denoted by symbol ‘*η*’ is commonly referred as additional potential required to drive an electrochemical reaction, which is obtained from the difference between thermodynamically determined potential and experimental equilibrium potential of a redox reaction. The reason is a real system will never proceed as an ideal system predicted only by theoretical calculations. Based on Anantharaj et al., the overall overpotential obtained in HER and OER is made up of three sources, namely activation overpotential, concentration overpotential, and ohmic overpotential [140]. For an electrochemical reaction to occur, the system must have sufficient energy to overcome an activation energy barrier, this requirement is known as activation overpotential [141, 142]. Jeremiasse et al. had reported methods of lowering the activation overpotential for electrocatalytic HER, which is done by employing trustworthy noble metal‐based cathodes and increasing the specific catalytic surface area [143]. Hence, developing a competent electrocatalyst that greatly minimizes the activation overpotential is the first step in achieving highly efficient electrocatalytic process. Next, concentration overpotential is a situation where imbalance of the concentration gradient occurs between the reactants and products on the catalytic surface [142, 144]. In other words, the rate of reactants adsorption and products desorption from the catalytic surface that is unable to achieve equilibrium leads to an increase of concentration overpotential. Additionally, concentration overpotential caused by build‐up of a pH gradient during PCET reactions, such as OER can be overcame by adding supporting buffer ions [145]. Ohmic overpotential encountered in OWS comprises of various physical resistances within the electrochemical system, namely resistances present in the electrodes, electrolyte, membranes, H_2_ and O_2_ bubbles, and external wire connection [146, 147]. Ohmic drop compensation (also known as iR correction, where *E*_(iR‐corrected)_ = *E*_(RHE)_ − iR) strategies such as selecting high electrical conductivity electrolytes, closing the distance between the cathode and anode, preventing bubbles formed on electrodes’ surface, and proper wiring connections can be utilized in reducing the ohmic overpotential [148]. In conclusion, lowering the overpotential in all forms (physical, chemical, intrinsic) of an electrochemical system ensures a better electrocatalytic performance.

#### 2.5.2 Tafel Slope

As one of the key evaluation parameters, Tafel slope's greatest purpose is providing information of the rate determining steps (RDS) by comparing the experimental and theoretical value of the electrochemical reaction [149]. Prior to achieving this result, Tafel plot is constructed based on an increasing overpotential against a rising current density or replotting the linear sweep voltammogram (LSV) curves with logarithmic current densities value, and the linear portion of the plot is fitted by the Tafel equation as shown in Equation 17 [150, 151].

(17) η=blogj+a

where *η* represents the overpotential, *b* denotes the Tafel slope, *j* is the current density, and *a* is the intercept relative to the exchange current density [152]. From the equation, a relationship between the parameters (*η* informs about the potential difference of the reaction; *b* elucidates the catalytic mechanism of the electrocatalytic reaction; while *j* describes the intrinsic properties of the electrocatalytic material under equilibrium conditions) [153] can be established where a lower *η* value required to achieve a fixed *j* value leads to a smaller *b* value, which indicates a higher reaction kinetics. Additionally, Tafel slope values of 120, 40, and 30 mV dec^−1^ signified Volmer, Heyrovsky, and Tafel EDS [151], and a low Tafel slope below 30 mV dec^−1^ is often associated with Volmer–Tafel mechanism, while Tafel slope between 40–120 mV dec^−1^ represents Volmer–Heyrovsky mechanism.

#### 2.5.3 Current Density (*j*) and Exchange Current Density (*j*_0_)

Current density, denoted by *j* can be referred as the amount of current traveling through the electrocatalyst with a specific cross‐sectional area, and it is mostly used in analyzing the electrocatalyst's efficiency. Generally, recording the overpotential at current density of 10 mA cm^−2^, denoted by *η*_10_ is the normalized technique in evaluating an electrocatalyst. However, Zhang et al. reported that large current densities up to *η*_1000_ should be attained by electrocatalysts for industrial applications [154]. When the electrochemical reaction reaches an equilibrium state, catalytic activities of an electrocatalyst can be evaluated using exchange current density (*j*_0_), which can be obtained through two methods: (1) the current density of Tafel equation with *η* assumed to be zero; (2) the current density at the interception of an extrapolated linear fit on the Tafel plot [155]. A high *j*_0_ is an indication towards an efficient electrocatalyst as the electron transfer across the electrocatalyst surface will require very low activation energies. Hence, the resultant overpotential of the system would be low and is highly desirable.

#### 2.5.4 Turnover Frequency (TOF)

TOF is one of the best figures of merit to implement when comparing the intrinsic activities of different electrocatalysts, which corresponds to the number of reactant evolved into product (H_2_ in HER and O_2_ in OER) per catalytic active site per unit time [156]. TOF of an electrocatalyst can be obtained from the Equation 18 [157].

(18) $$\mathrm{TOF}=\left(j\;\times \;N_{A}\right)\;{\left(F\;\times \;n\;\times \;\tau \right)}^{-1}$$

where *j* represents the current density, *N*_A_ is the Avogadro cumber, *F* stands for Faraday constant, *n* is the number of electrons transferred, and *T* depicts the surface concentration or active site numbers catalyzing the reaction. At present, the biggest challenge of TOF is to obtain the precise TOF value for heterogeneous catalytic materials, which is due to certain inaccessible internal atoms or catalytic species. Hence, TOF is often calculated solely based on the easily attainable atoms or catalytic groups on the surface of the electrocatalysts [153]. This method is the most reasonable approach and widely implemented by researchers but it clearly does not provide the exact value.

#### 2.5.5 Mass Activity (MA), Specific Activity (SA), and Electrochemically Active Surface Area (ECSA)

MA (current normalized by the loaded catalyst mass, mA mg^−1^) and SA (current normalized by electrochemically active surface area (ECSA) or Brunauer–Emmett–Teller (BET), mA cm^−2^) are two alternative quantitative parameters used to investigate the catalytic performance and a higher MA/SA value represents a greater efficiency of the electrocatalyst [158]. Examining deeper on the intrinsic stage, ECSA ensures a more promising strategy recently as compared to BET due to the inaccuracy of BET measurements discussed in literature. [159, 160] Generally, ECSA depicts the area accessible to the electrolyte that involves directly in the electrocatalytic conversion, which is extremely significant in electrochemical studies. ECSA of an electrocatalyst can be calculated from Equation 19 [161].

(19) $$\mathrm{ECSA}=\left(C_{\mathrm{dl}}\right){\left(C_{s}\right)}^{-1}$$

where *C*_dl_ represents the double layer capacitance and *C*_s_ is the specific capacitance of a flat surface of the catalyst. ECSA can be evaluated by the electrochemical double‐layer capacitance (*C*_dl_) of cyclic voltammetry (CV) measurements at different scan rates.

#### 2.5.6 Catalyst Stability

Apart from the aforementioned evaluation criteria that focus on the catalytic activities, catalyst stability is also a vital indicator that determines its potential for commercialization. Therefore, abiding durability of the catalysts and its factors must be identified and accessed. Currently, there are two common methods for evaluating catalyst stability: (1) cyclic voltammetry (CV) and (2) chronoamperometry or chronopotentiometry. CV method is an accelerated degradation test that subjects the catalyst to 1000 cycles of experiments, where the shift in onset overpotential is taken as a representative measurement of catalyst stability. Next, chronoamperometry or chronopotentiometry curves are recorded after operating for more than 12 h at fixed current densities, where a change in the current density (at fixed potential) or overpotential (at fixed current density) reveals the stability of the catalysts [160]. In short, a stable catalyst that is feasible for practical applications must be able to work under large current densities over a long period of time with negligible alterations.

## 3 Unifunctional C*_x_* N*_y_* Based Electrocatalysts for HER and OER

### 3.1 Defect Engineering

#### 3.1.1 Doping Engineering

As part of the carbon‐based family, pristine C*_x_* N*_y_* material is an excellent candidate for HER electrocatalysts due to its high flexibility, nitrogen content abundancy, and superb thermal and chemical stabilities. Nevertheless, the material's intrinsic catalytic activities can be further enhanced by incorporating non‐metal anionic dopants from common p‐block elements at different doping sites. Elemental sulfur (S), phosphorus (P), and boron (B) are most common p‐block dopants and deemed worthy for HER electrocatalysts due to their corrosion resistance [162, 163], which is highly compelling towards the recent development of corrosion engineered seawater splitting electrocatalysts for hydrogen production purposes [164, 165]. In addition, doping C*_x_* N*_y_* catalysts with S, P, and B element was investigated computationally and experimentally that S, P, and B dopants could improve the electrical conductivity of g‐C_3_N_4_ [166, 167], creating an electrocatalyst that carries high charge densities. First‐principles studies have also been conducted on doping C*_x_* N*_y_* with transition metals to explore high‐efficiency electrocatalyst for HER and OER [99, 168, 169, 170].

**FIGURE 5:**
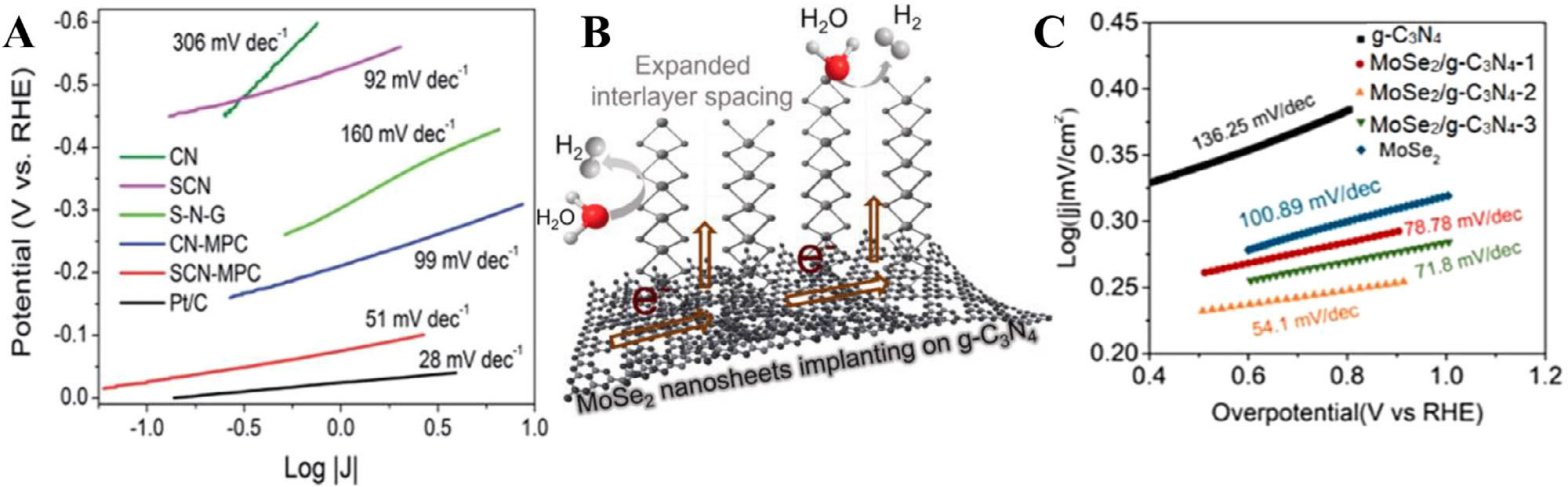
(A) Tafel plots of SCN‐MPC and other varied catalysts (CN: pristine g‐C_3_N_4_, SCN: S‐doped g‐C_3_N_4_, S‐N‐G: S and N co‐doped graphene, Pt/C: Platinum on carbon). Reproduced with permission [175]. Copyright 2016, The Royal Society of Chemistry. (B) Schematic illustration of vertical implantation procedure of MoSe_2_ nanosheets on superior thin g‐C_3_N_4_ nanosheets. (C) Tafel plots of MoSe_2_/g–C_3_N_4_ and other varied catalysts. Reproduced with permission [176]. Copyright 2024, Elsevier Inc.

As the pioneer of S doping on C*_x_* N*_y_* materials for electrocatalytic HER, Pei et al. had successfully synthesized a S‐doped g‐C_3_N_4_ electrocatalyst supported on mesoporous carbon (SCN‐MPC) by in situ polycondensation. The as‐synthesized electrocatalyst recorded an overpotential at 10 mA cm^−2^ ($\eta_{10}$) of 145 mV and Tafel slope of 51 mV dec^−1^, which was considerably better than the pristine g‐C_3_N_4_ supported on MC (CN‐MPC) with a Tafel slope value of 99 mV dec^−1^ in 0.5 m H_2_SO_4_ solution (Figure 5A). Notably, the improved results of the S‐doped electrocatalyst was contributed by the smaller bandgap structure of S‐doped g‐C_3_N_4_ (0.20 eV) as compared to pristine g‐C_3_N_4_ (approximately 2.70 eV), which implied efficient charge transfer [171, 172]. To further acquire an accurate depiction of the relationship between S‐doping sites and improved electrocatalytic HER activity, density functional theory (DFT) calculations were performed on different doping sites (N1, N2, N3, C1, C2). In accordance with the DFT results, the most optimal HER activity was recorded by doping S atom on the pyridinic N3 sites, which led to the redistribution of charge and spin density of the S‐doped N atoms, synergistically enhancing the HER activity by lowering Gibbs free energy of H^+^ adsorption [173, 174], giving rise to a remarkably small ∆*G*(H_ads_) value of 0.03 eV and serving as a major solution to pyridinic N active sites of pristine g‐C_3_N_4_ with ∆*G*(H_ads_) of 0.58 eV [175]. In short, the magnificent experimental results corresponded well with the computational findings, which initiated more researches to examine deeper into non‐metal dopants.

Zhang et al. synthesized MoSe_2_ nanosheets vertically implanted on ultrathin C‐doped g‐C_3_N_4_ nanosheets (Figure 5B) via Mo─N bonding to develop an electrocatalyst with enhanced electrochemical activity [176]. Using a solvothermal method, the uniform growth of MoSe_2_ improved its dispersibility and exposed more active edge sites. By using optimized preparation parameters, the MoSe_2_/g–C_3_N_4_^−2^ exhibited superior HER performance in acidic electrolytes, achieving a Tafel slope of 54.1 mV dec^−1^ and an overpotential of 258 mV at 10 mA cm^−2^, compared to 100.89 mV dec^−1^ and 319.3 mV for pure MoSe_2_ (Figure 5C). The Tafel slope of the composite samples, falling within the range of 40–120 mV dec^−1^, indicates that the HER process follows the Volmer–Heyrovsky mechanism [177]. The heterostructure demonstrated high stability with negligible degradation after 1000 CV cycles, and XPS and TEM analyses confirmed the retention of morphology and composition. Mechanistically, the g‐C_3_N_4_ nanosheets provided a large surface area for MoSe_2_ dispersion, while carbon doping introduced N vacancies, enhancing active sites and lowering charge transfer resistance. The results showed improved mass activity (23.7 mA mg^−1^) and current density (4.8 mA cm^−2^ at −0.3 V vs. RHE) for the heterostructure compared to pure MoSe_2_, underscoring the synergy between the components for efficient HER.

**FIGURE 6:**
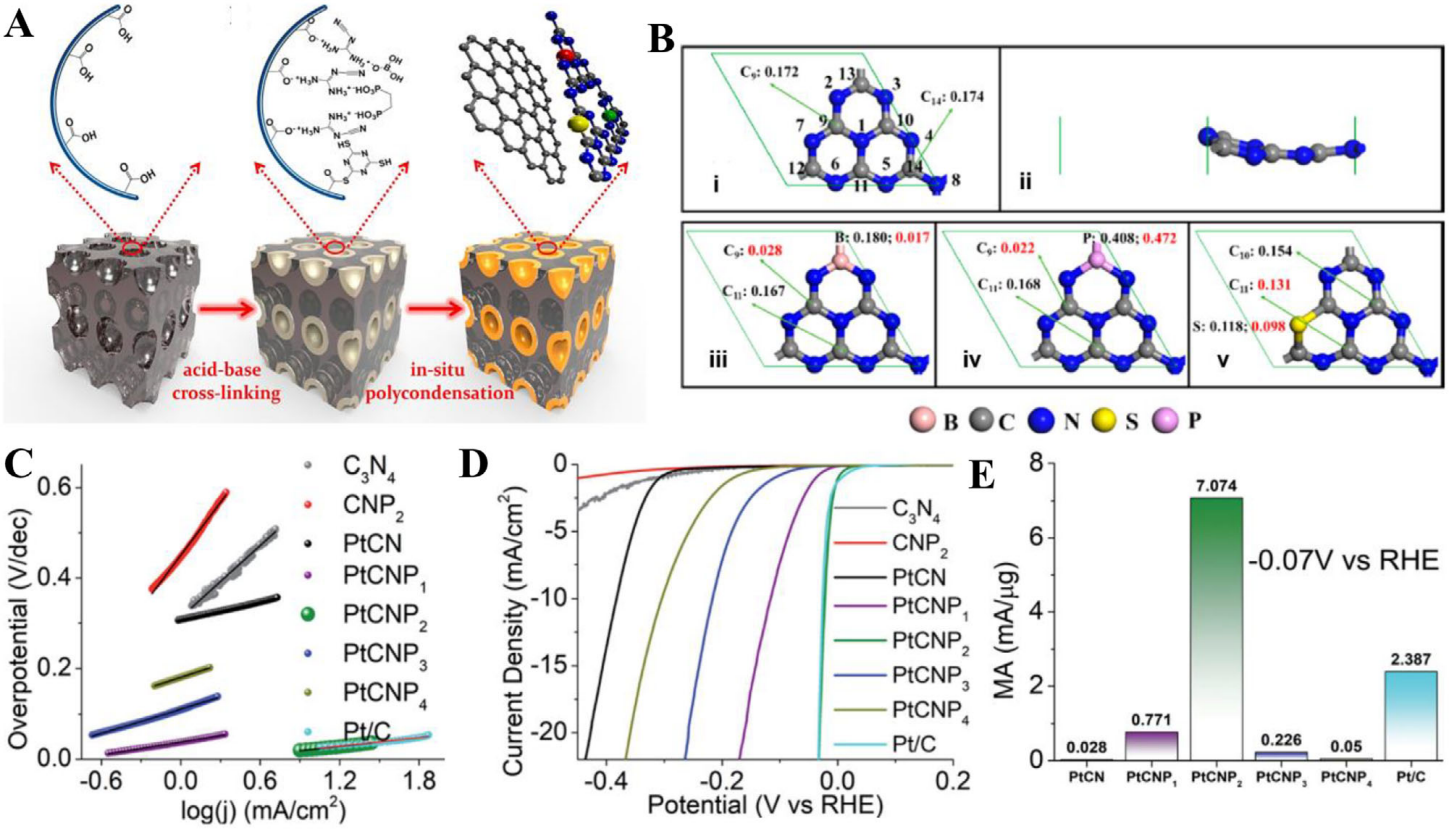
(A) Schematic illustration for the synthesis of B‐, P‐, S‐CN/PC electrocatalysts. (B) Schematic representation of interstitial and substitutional atomic modification by non‐metal doping into the fcc Pd host lattice structure. Reproduced with permission [180]. Copyright 2017, American Chemical Society. (C) DFT calculated free‐energy diagram of HER. (D) Schematic illustration of the synthetic process of PtCNP. (E) Tafel plots of various electrocatalysts. Reproduced with permission [184]. Copyright 2022, The Royal Society of Chemistry.

Inspired from their previous work [175], Pei et al. had simultaneously investigated three non‐metal p‐block dopants (boron (B), phosphorus (P), and sulfur (S) on g‐C_3_N_4_ with a different support (macroporous carbon denoted by PC) via in situ polycondensation of B, P, S precursors with g‐C_3_N_4_ supported by PC (Figure 6A), giving rise to three non‐metal doped electrocatalysts: B‐CN/PC, P‐CN/PC, and S‐CN/PC. Generally, there are two modes of doping: (1) interstitial doping occurs when foreign elements occupy interstitial sites of the host structure, while (2) substitutional doping involves foreign elements replacing atoms of the host [178, 179]. To identify the mode of doping, DFT computations were carried out to determine the optimized structure of pure g‐C_3_N_4_ as well as the most thermodynamically favorable doping positions of B, P, and S (Figure 6B). Similarly, all three dopants were subjected to substitutional doping, but they preferred different doping sites. B and P atoms’ most favorable site was the bay‐carbon (C13), while pyridinic N (N7) was more stable to be substituted by S atoms. Among these electrocatalysts, S‐CN/PC's HER performance triumphed over its peers with $\eta_{10}$, Tafel slope, and ∆*G*(H_ads_) of 186 mV, 84 mV dec^−1^, and 0.03 eV, while B‐CN/PC with 405 mV, 93 mV dec^−1^, 2.25 eV, and P‐CN/PC with and 343 mV, 100 mV dec^−1^, 1.87 eV in 0.5 m H_2_SO_4_ solution [180]. Intrinsically, S‐dopant on the g‐C_3_N_4_ structure introduces an impurity band near the conduction band minimum (CBM) which favors the protons to accept electrons during HER, [181] hence the superb electrocatalytic HER activities. Between two S‐doped electrocatalysts, SCN‐MPC ($\eta_{10}$ of 145 mV, Tafel slope of 51 mV dec^−1^) displayed a better electrocatalytic activity in contrast to S‐CN/PC, this implied that the difference in the support's pore size of SCN‐MPC (mesoporous) and S‐CN/PC (macroporous) will greatly affect the electrocatalyst's capabilities. Despite its amazing catalytic activities, both S‐doped g‐C_3_N_4_ electrocatalysts were far from being on par with the commercial platinum on carbon (Pt/C) electrocatalyst for HER with a Tafel slope value of 28 mV dec^−1^ and $\eta_{10}$ of 31 mV. One of the possible reasons is due to Pt's metallic characteristics (high thermal and electrical conductivity, low resistance, able to carry huge charge densities) that grants it to be a dominant material in the electrocatalysis field.

Currently, commercial Pt/C is undoubtedly the best electrocatalyst for HER but is limited to large‐scale applications on account of their scarcity, high cost and low durability [182]. A resolution to this phenomenon was utilizing g‐C_3_N_4_ as a scaffold to incorporate of P and Pt atoms, which was able to retain certain characteristics of the Pt and synergistically improve the electrocatalytic activity with the doped P atoms. Such electrocatalyst was successfully synthesized by Nichols et al. through a two‐step P doping and Pt complexation process, four samples of P‐doped Pt‐g‐C_3_N_4_ (PtCNP_1_, PtCNP_2,_ PtCNP_3_, and PtCNP_4_) electrocatalysts were prepared. Despite having the similar synthesis route, X‐ray photoelectron spectroscopy (XPS) measurements confirmed that each sample had different P contents with PtCNP_1_ (1.13 at%), PtCNP_2_ (1.11 at%)_,_ PtCNP_3_ (6.32 at%), and PtCNP_4_ (11.03 at%). Doping of P atoms had contributed to the structural defects of g‐C_3_N_4_, the increasing C: N atomic ratios of the samples in the order of PtCN (0.86), PtCNP_1_ and PtCNP_2_ (0.96), PtCNP_3_ (1.04), and PtCNP_4_ (2.75) implied that N atoms were gradually being replaced by the doped P atoms in the g‐C_3_N_4_ structure, which was obviously regarded as substitutional doping. Under 0.5 m H_2_SO_4_, the HER performance recorded in Figure 6C–E showed that PtCNP_2_ ($\eta_{10}$ of 22 mV, Tafel slope of 31.2 mV dec^−1^, and MA of 7.074 mA µg^−1^) with the lowest P composition was on par and even slightly better than Pt/C due to the synergistic effect of P atoms leading an electron enrichment towards Pt which improved the overall HER charge transfer [183], and structural defects of g‐C_3_N_4_ induced by P dopants favored the attachments of protons. [184] In summary, interstitial doping of mono‐atoms on g‐C_3_N_4_ for electrocatalytic HER is still lacking and can be focused on upcoming researches. Logically thinking, interstitial doping in between the g‐C_3_N_4_ sheets is anticipated to bring about new interlayer chemical bonds between the dopant and tri‐*s*‐triazine structures that ultimately boost the electrocatalytic process.

**FIGURE 7:**
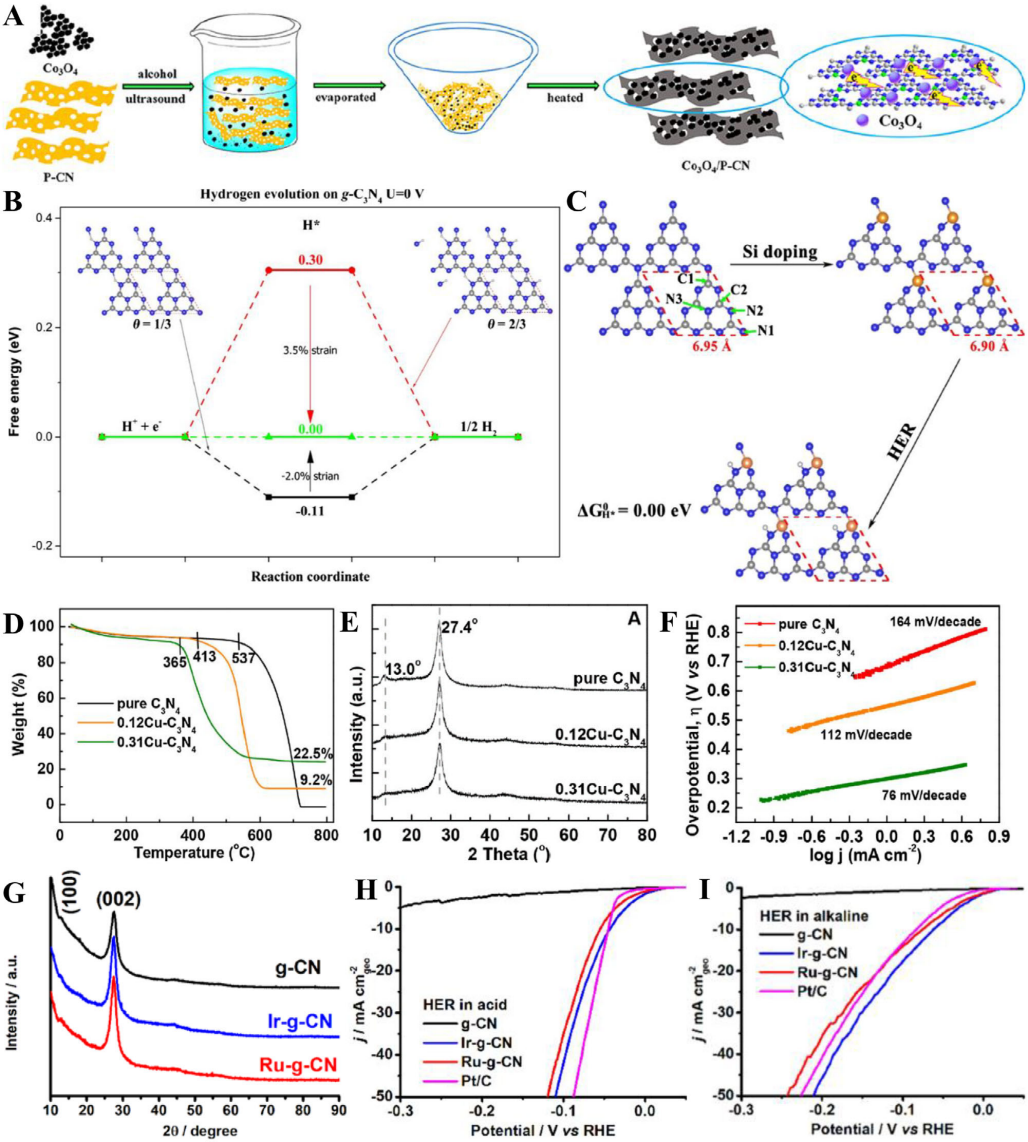
(A) Schematic illustration of the process of P‐doped CO_3_O_4_/P‐CN electrocatalyst preparation. Reproduced with permission. [185] Copyright 2019, American Chemical Society. (B) Free energy diagram for HER on strained g‐C_3_N_4_ under hydrogen coverages (*θ*) of *θ* = 1/3 and *θ* = 2/3. Coverage of 1/3 is defined by one H_ads_ atom on one g‐C_3_N_4_ unit cell, while *θ* = 2/3 means two H_ads_ atoms. (C) Catalyst stability test of 0.31Cu‐C_3_N_4_ at an overpotential of 390 mV. Reproduced with permission [189]. Copyright 2015, Elsevier Inc. (D) Thermogravimetric analysis (TGA) for pure g‐C_3_N_4_, 0.12Cu‐C_3_N_4_, and 0.31Cu‐C_3_N_4_. Molar ratios Cu/g‐C_3_N_4_ of 0.12Cu‐C_3_N_4_ and 0.31Cu‐C_3_N_4_ were 0.12/1.00 and 0.31/1.00, respectively. (E) X‐ray diffraction (XRD) patterns and (F) Tafel slopes of g‐C_3_N_4_, 0.12Cu‐C_3_N_4_, and 0.31Cu‐C_3_N_4_. Reproduced with permission. [197] Copyright 2015, Elsevier Inc. (G) XRD patterns of g‐CN, Ir‐g‐CN, and Ru‐g‐CN. LSV curves of g‐CN, Ir‐g‐CN, Ru‐g‐CN, and Pt/C in (H) acidic and (I) alkaline media. Reproduced with permission [198]. Copyright 2022, Elsevier Inc.

In OER electrocatalysis, p‐block dopants were investigated as well by virtue of its superior electrical conductivity. Zhu et al. fabricated Co_3_O_4_ nanocrystals with different mass content (5%, 20%, 40%, 60%, and 80%) supported on phosphorus‐doped g‐C_3_N_4_ (Co_3_O_4_/P‐CN) as illustrated in Figure 7A. The as‐synthesized electrocatalysts were subjected to electrocatalytic OER experiments in a three‐electrode electrolytic cell under alkaline conditions (1.0 m KOH) and yielded satisfactory results with $\eta_{10}$ and Tafel slope values ranging from 320–428 mV and 65–106 mV dec^−1^ (lowest $\eta_{10}$ and Tafel slope value was recorded by 60% Co_3_O_4_/P‐CN) owing to the synergistic effects between Co_3_O_4_ and P‐CN. Naturally, OER's four PCET step acknowledged previously is the origin of large overpotentials and sluggish kinetics, which dampens the OER as well as OWS process. The introduction of P dopants into the Co_3_O_4_/P‐CN hybrid had effectively improved the electrical conductivity, which was proven by the impressively lower charge‐transfer resistance (*R*_ct_) of the most optimal 60% Co_3_O_4_/P‐CN electrocatalyst at 0.524 Ω in opposition to pristine CN and Co_3_O_4_ at 20,000 and 22.8 Ω, respectively. The lower *R*_ct_ value induced by the synergistic coupling of Co_3_O_4_ and P‐doped CN assisted the electron transfer process by decreasing the charge recombination rate, further empowering the charge transfer process of OER [185]. In another study, porous B‐doped g‐C_3_N_4_ nanosheets (B‐doped g‐C_3_N_4_) were synthesized by thermal polymerization utilizing H_3_BO_3_ and urea as precursors. At 0.1 m KOH solution, B‐doped g‐C_3_N_4_ (Tafel slope of 50.6 mV dec^−1^) attained a greater electrocatalytic OER results relative to that of pristine g‐C_3_N_4_ (Tafel slope of 92 mV dec^−1^) [186]. Altogether, p‐block dopants had consistently empowered both HER and OER electrocatalysts primarily by the strength of their electrical conductivity.

Based on the strain regulation principle, the existence of compressive or tensile strain within an electrocatalyst was proven to enhance the HER performance. Compressive strain is defined as strain induced by the shortening of the 2D materials; while tensile strain is caused by the lengthening of the 2D materials [187]. In relation to HER, strain engineering effects are often evaluated through the adsorption and desorption energies of reactive intermediates such as hydrogen itself [188]. Strain engineering effects were realized by Gao et al. through the substitutional doping of a bridge C atom on the g‐C_3_N_4_ structure with a silicon (Si) atom. Prior to Si doping, the ideal ∆*G*(H_ads_) = 0.00 of strained g‐C_3_N_4_ under hydrogen coverages of 1/3 and 2/3 was determined by using DFT calculations. It was observed that a compressive strain (–2.0 %) raised the ∆*G*(H_ads_) from –0.11 to 0 eV, while a tensile stress (3.5 %) reduced the ∆*G*(H_ads_) from 0.30 eV to 0 eV (Figure 7B), which implied that a small positive or negative strain was able to tune the catalytic properties of g‐C_3_N_4_. As depicted in Figure 7C, different Si doping sites (C1, C2, N1, N2, N3) on g‐C_3_N_4_ were investigated by theoretically computing the formation energies (∆*E*_form_). The most optimal doping site for Si was located at C1 (bridge C atom) with the lowest ∆*E*_form_ of 2.51 eV when comparing with adjacent doping sites C2 (3.29 eV), N1 (3.27 eV), N2 (2.65 eV), and N3 (4.45 eV) as a lower ∆*E*_form_ of modified electrocatalyst is always desired due to practical implementations. Also, Si‐doping induced obvious deformation around the Si doping site by reducing the equilibrium lattice constant of g‐C_3_N_4_ from 6.95 to 6.90 Å. Despite the tuning of g‐C_3_N_4_ framework, the Si atoms did not introduce odd electrons into the overall system, thus it was concluded that the hydrogen adsorption properties of Si‐doped g‐C_3_N_4_ behaved similarly to those of pristine g‐C_3_N_4_ [189]. In essence, strain engineering via heteroatom doping has huge potential in electrocatalysis owing to part of the mechanical deformation energy is converted into chemical reaction energy, as well as the beneficial properties offered by the dopant itself.

Beyond non‐metal dopants, metal doping on C*_x_* N*_y_* is a promising strategy as metal dopants (e.g., Pt, Fe, Co, Ni, Mn, Mo, Cu) have been displaying great potential to lower electrical resistivity [190], amplify electron transfer [191], increase electronic density [192, 193], activate and enhance catalytic active sites [165, 194, 195]. On account of its abundancy, low cost, and ionic stability, Cu is amongst the most widely used transition metal [196]. For instance, Cu‐doped g‐C_3_N_4_ (Cu‐C_3_N_4_) was synthesized through a one‐step self‐assembly procedure by heating a mixture of dicyandiamide and copper(II) chloride at 500°C. The resulting electrocatalyst demonstrated a magnificent electrochemical stability after 43 h under acidic condition, but the thermal stability of Cu‐C_3_N_4_ samples dropped drastically (Figure 7D) in comparison to the pristine g‐C_3_N_4_ with the increase of Cu composition (pristine g‐C_3_N_4_ stable up to 537°C; 0.12Cu‐C_3_N_4_ stable up to 413; 0.31Cu‐C_3_N_4_ stable up to 365°C). The diminished thermal stability was attributed to the strong influence of Cu dopants that weakened the chemical bonds in g‐C_3_N_4_. Moreover, the XRD patterns revealed that Cu‐C_3_N_4_ samples and pristine g‐C_3_N_4_ had similar structures (Figure 7E), which suggested that Cu dopants not only maintained the structural characteristics of g‐C_3_N_4_ but also enriched the intrinsic electrocatalytic properties of the electrocatalyst as shown Figure 7F [197]. Likewise, the XRD patterns in single‐metal‐atom doping of iridium (Ir) and ruthenium (Ru) on g‐C_3_N_4_ (Figure 7G) did not detect any alteration of the g‐C_3_N_4_ structure, which suggested that the Ir and Ru dopants were atomically dispersed. Despite the tiny amount of dopants, Ir‐g‐CN (Ir at 1.71 wt%) and Ru‐g‐CN (Ru at 0.24 wt%) electrocatalysts’ HER activities were on par with the commercial Pt/C electrocatalyst (Figure 7H,I) in both acidic (0.5 m H_2_SO_4_) and alkaline media (1.0 m KOH). In this research, Yu et al. also elucidated the supremacy of g‐C_3_N_4_ as a dopant support via their electrochemical stability in anodic oxidation situations. Ir‐g‐CN's degradation rates were 0.45 and 0.87 mV h^−1^ in acidic and alkaline solution, respectively, and those of Ru‐g‐CN were 0.66 and 1.11 mV h^−1^, which were lower than that of commercial Pt/C (0.96 and 1.58 mV h^−1^) [198]. Higher electrochemical stability of Ir‐g‐CN and Ru‐g‐CN originated from the strong chemical bonding between the single metallic Ir/Ru atoms and N atoms of g‐C_3_N_4_, while Pt nanoparticles were weakly bonded to the carbon support [199]. Furthermore, the degradation rates of aforementioned electrocatalysts were uniformly higher in acidic conditions, which were assigned to the higher H_2_ production in acidic media leading to a higher corrosion rate by the evolved H_2_ bubbles [200]. This issue can be immaculately resolved by doping of P‐block elements (S, P, B) in recognition to their corrosion resistance as highlighted previously.

**FIGURE 8:**
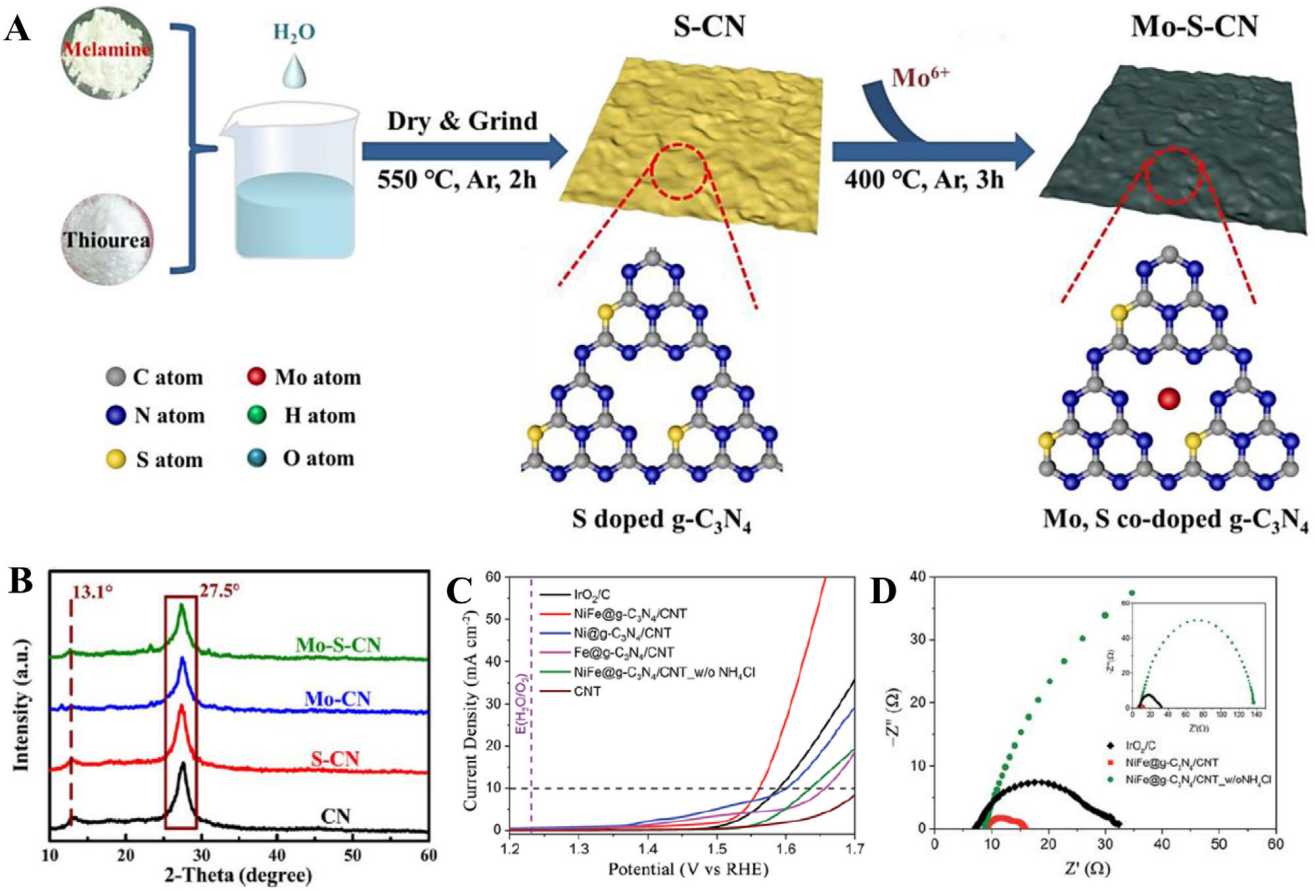
(A) Schematic representation of the formation of Mo‐S‐CN. (B) XRD patterns of Mo‐S‐CN, Mo‐CN, S‐CN, and CN. Reproduced with permission [204]. Copyright 2020, Elsevier Inc. (C) Polarization curves of NiFe@g‐C_3_N_4_/CNT and other electrocatalysts. (D) Nyquist plots of NiFe@g‐C_3_N_4_/CNT and other electrocatalysts. Reproduced with permission [206]. Copyright 2018, The Royal Society of Chemistry.

Since each metal or non‐metal dopant functions via a unique mechanism, the act of co‐doping serves as a viable option to introduce synergistic effects that further elevate the performance of the electrocatalyst beyond those of mono‐doped [201]. In an alkaline electrocatalytic HER study, Mo and S co‐doped g‐C_3_N_4_ (Mo‐S‐CN) was constructed through a simple thermal polycondensation route (Figure 8A) and compared with the single atom doped g‐C_3_N_4_ (Mo‐CN and S‐CN) electrocatalysts. Obviously, Mo‐S‐CN produced greater outcomes in 1.0 m KOH solution with $\eta_{10}$ and Tafel slope of 630 mV and 110 mV dec^−1^, accordingly than Mo‐CN (750 mV and 178 mV dec^−1^) and S‐CN (840 mV and 197 mV dec^−1^). Nevertheless, there was a sharp distinction of HER performance between Mo‐S‐CN and S‐CN in this work with SCN‐MPC (145 mV and 51 mV dec^−1^) [175] and S‐CN/PC (186 mV and 84 mV dec^−1^) [180] discussed previously. This phenomenon occurred because of two reasons: (1) pH value of the environment and (2) usage of electrocatalyst support. Since HER activities of SCN‐MPC and S‐CN/PC were carried out in acidic media (0.5 m H_2_SO_4_), the abundancy of H^+^ could induced a proton rich layer on the electrocatalyst surface that eased the adsorption; while Mo‐S‐CN and S‐CN under alkaline media (1.0 m KOH) could only rely on the protons generated from the water dissociation process, which ultimately led to a higher energy barrier of alkaline HER kinetics [202]. Another study also concluded that during alkaline HER, extra OH^−^ competed with H^+^ for a single catalytic active site, hence weakening the production of H_2_ [203]. Despite the dissimilarity of pH conditions, the as‐synthesized Mo‐S‐CN electrocatalyst behaved similarly as SCN‐MPC and S‐CN/PC in the HER kinetics via Volmer–Heyrovsky mechanism. In this co‐doping process, N atoms of g‐C_3_N_4_ were replaced by S atoms (substitutional doping) and Mo atoms were bonded in between S and surrounding N atoms (interstitial doping). The essence of Mo─S─CN's synergistic effect came from the more electropositive Mo centers (transfer of electrons from Mo to N) that allowed the attachment of O atom of H_2_O for water dissociation, followed by S acting as the electron donor to the Mo─O─H─O bond, further lengthened the H─O bond length by forming π‐back bond and accelerated the water dissociation process. In short, the synergistic electron transfer in Mo─S─CN in contrary to the single atom doped and pristine counterparts led to a better HER activity [204]. With regard to Mo─S─CN, the absence of the catalyst support as well led to the underperformed catalytic activities as catalyst‐support interactions are known to be effective in improving the electrocatalytic properties by modulating the electronic structure, amplifying electron transfer, tweaking the catalytic adsorption properties, and most importantly stabilizing the electrocatalyst [205]. As depicted in Figure 8B, the g‐C_3_N_4_ structure of co‐doped Mo─S─CN was retained, similar to those of single atom doped g‐C_3_N_4_ electrocatalyst.

In OER co‐doping event, Liu et al. presented the fabrication of Ni and Fe dual‐metal atom doped g‐C_3_N_4_ supported on carbon nanotube bundles (NiFe@g‐C_3_N_4_/CNTs) by in situ high temperature polymerization assisted by NH_4_Cl template (Figure 8C). This dual‐metal doping strategy had triggered a positive synergistic effect that accelerated the electron transfer between Ni and Fe and promoted the OER activity by regulating the electronic structure. The electronic structure reconfiguration of NiFe@g‐C_3_N_4_/CNTs brought upon remarkable OER performance in 1.0 m KOH solution of $\eta_{10}$ and Tafel slope of 67 mV and 326 mV dec^−1^, which was on par with the commercial IrO_2_ benchmark as shown in the OER polarization curves (Figure 8C). NiFe@g‐C_3_N_4_/CNTs also showcased a FE of 99.6% with insignificant decrease of catalytic activities after 3000 cycles), indicated that the as‐synthesized electrocatalyst was suitable for long‐term electrochemical OER processes. Besides, this research also highlighted the significance of NH_4_Cl template in the synthesis procedures, it was observed on the Nyquist plots that NiFe@g‐C_3_N_4_/CNTs with NH_4_Cl and without NH_4_Cl (Figure 8D) showcased a massive distinction in the semicircle sizes which reflected the *R*_ct_ values [206]. All things considered, both non‐metal and metal dopants had shown their added values for g‐C_3_N_4_ HER and OER electrocatalysts. On certain occasions, multi‐atom doping strategy were explored and employed to realize new synergistic effects to provide insights for future researchers. There were also other modification strategies coupled with doping such as forming hybrid and using catalyst supports, these simultaneous modification efforts had been proven to be promising in finding the perfect replacement for state‐of‐the‐art noble metal electrocatalysts.

#### 3.1.2 Vacancy

Vacancy is a specific type of point defect engineering and the introduction of vacancy in electrocatalysts preparation promotes the catalytic process in multiple ways, including: (1) tuning the electronic structure, (2) augmenting catalytic active sites, and (3) improving the electrical conductivity. Wu et al. had recently categorized different kinds of vacancies comprised of anionic vacancies, cationic vacancies, and multi vacancies [207]. In regard to C*_x_* N*_y_* materials, this classification can be further adapted with neutral vacancies that involve atoms without charges, namely carbon and nitrogen vacancies.

**FIGURE 9:**
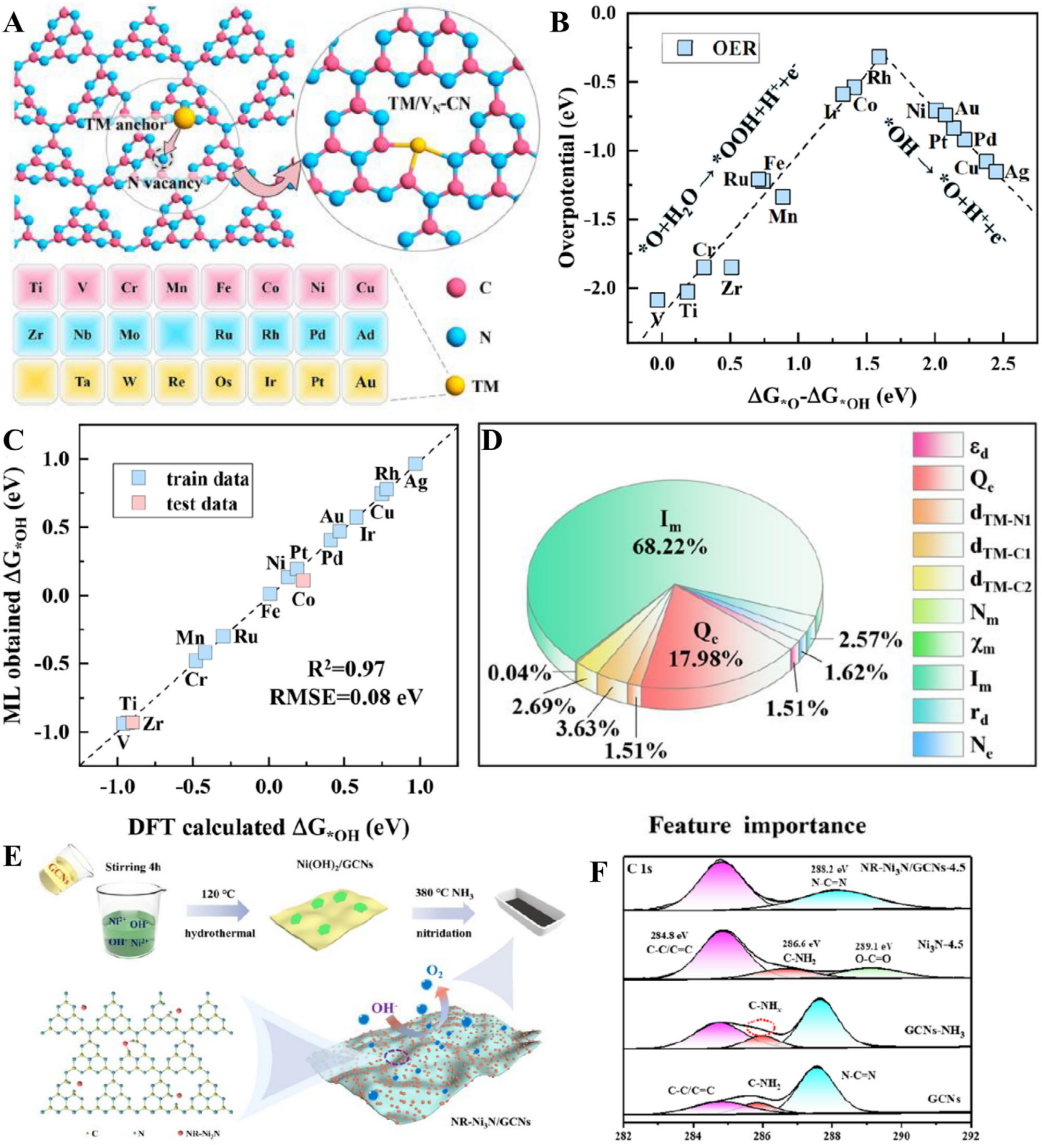
(A) Schematic illustration of TM/V_N_‐CN and different TM atoms. (B) Volcano plot of different TM atoms in simulated OER. (C) Comparison plot of ∆G_*OH_ between DFT and GBR model of ML. (D) Feature analysis of the GBR model, where *ε*_d_ is d‐band center, *Q*_e_ represents charge transfer of TM atoms, d_TM‑N1_, d_TM‑C1_, and d_TM‑C2_ is the bond length between TM and coordination atoms, *N*_m_ implies the electronegativity, *X*_m_ represents electron affinity, *I*_m_ is the first ionization energy, *r*_d_ is the radius of TM atom, *N*_e_ represents the number of TM‐d electrons. Reproduced with permission [208]. Copyright 2021, American Chemical Society. (E) Schematic illustration of the synthetic procedure for defective g‐C_3_N_4_ composite (NR‐Ni_3_N/GCNs). (F) High resolution XPS spectra of C 1s for NR‐Ni_3_N/GCNs‐4.5, Ni_3_N‐4.5, GCNs‐NH_3_, and GCNs, where 4.5 in Ni_3_N‐4.5 and NR‐Ni_3_N/GCNs‐4.5 represents the reaction time in hours. Reproduced with permission [209]. Copyright 2022, American Chemical Society.

Niu et al. combined DFT and machine learning (ML) in studying different transition metal single atoms supported on defective g‐C_3_N_4_ with N vacancies, denoted by TM/V_N_‐CN (Figure 9A). This computational research was carried out to determine the most optimal TM/V_N_‐CN as well as the governing factors that contributed towards the adsorption energies. Based on Figure 9B, Rh stood at the top of the volcano plot with ∆G_*O_—∆G_*OH_ of 1.62 eV. ML studies were carried out in three systematic steps: (1) data generation via DFT, (2) model training and test, and (3) feature analysis. Gradient boosted regression (GBR) model was utilized to examine the adsorption behaviors through a set of structural and atomic descriptors. After the first step was completed by DFT, the input data were randomly categorized into train and test data and the accuracy of GBR model was assessed by the coefficient of determination (*R* ^2^) and root‐mean‐square error (RMSE) using the train and test data. A high *R* ^2^ and low RMSE are expected for GBR model with high accuracy. During model training, GBR model achieved a near ideal *R* ^2^ of 0.99 and low RMSE of 0.03 eV, while model testing obtained *R* ^2^ of 0.97 and RMSE of 0.08 eV (Figure 9C), which indicated that GBR model was viable for the feature analysis. Among the 10 descriptors as shown in Figure 9D, the highest feature importance was scored by *I*_m_ (68.22%), followed by *Q*_e_ (17.98%) implied that these two features (*I*_m_ and *Q*_e_) had the greatest impact in the GBR model and more greatly associated with the adsorption behaviors among the others. Since *I*_m_ is defined as the energy needed to remove an electron from a neutral atom to form a positively charged ion, the *I*_m_ rises from left to right across a period by virtue of the increasing nuclear charge, which resulted the outermost electron being more strongly bounded to the nucleus [208]. This work displays comprehensive simulation efforts, which combines DFT and ML to identify the dominating factors of a highly efficient electrocatalyst. Despite that, experimental works could be carried out to verify the simulated results in order to make it even more meaningful.

On the other hand, Luo et al. introduced a convenient method to create a nitrogen‐deficient g‐C_3_N_4_ composite via two step synthetic procedure: (1) hydrothermal growth of Ni(OH)_2_ and g‐C_3_N_4_ precatalyst continued by (2) low temperature nitridation under NH_3_ atmosphere (Figure 9E). The nitride Ni‐rich Ni_3_N/nitrogen‐deficient g‐C_3_N_4_ OER electrocatalyst was denoted as NR‐Ni_3_N/GCNs [209]. Nitrogen vacancies of GCNs were induced by nitridation process, which were verified through the high resolution XPS spectra of C 1s. Based on Figure 9F, a more obvious C‐NH*_x_* signal was detected in the GCNs‐NH_3_ C 1s spectrum, which was a sign of defect vacancies. Likewise, the N/C ratio of pure GCNs and GCNs‐NH_3_ were reported to be 0.89 and 0.76, respectively, such decrement also implied the introduction of nitrogen vacancies upon nitridation. Nevertheless, the C‐NH*_x_* signal disappeared in the NR‐Ni_3_N/GCNs electrocatalyst (Figure 9F). During the nitridation process, nitrogen atoms from the Ni_3_N lattice were released as well and acted as a glue by forming a bridge connecting Ni_3_N and GCNs, which essentially compensated the nitrogen vacancy defect sites of the GCNs. The formation of Ni_3_N‐GCNs bond suggested the structural compatibility between the metallic species of Ni_3_N and non‐metallic species nitrogen‐deficient GCNs, which is in accordance with other metallic species coupled defective g‐C_3_N_4_ composites in other domains (e.g., transition metal atoms on g‐C_3_N_4_ with nitrogen vacancies (TM@NVs‐g‐C_3_N_4_) in electrocatalytic N_2_ reduction [210]; oxygen‐vacancy‐rich NiCo_2_O_4_/nitrogen‐deficient graphitic carbon nitride (O_v_‐NiCo_2_O_4_/ND‐g‐C_3_N_4_) in high performance supercapacitors [211]; and palladium oxide on zirconium‐doped vacancy‐abundant g‐C_3_N_4_ (Pd/Zr‐C_3_N_4_) in low‐temperature dehydrogenation of liquid hydrocarbons) [212]. In short, vacancy engineering of g‐C_3_N_4_ is a promising route in electrocatalysis, but the resultant vacancy‐rich electrocatalyst requires further modifications like doping or hybridization that could make use of the vacancy sites to enhance the electrocatalytic properties.

### 3.2 Structural Engineering

In the past decade, the status of g‐C_3_N_4_ in electrocatalysis has been experiencing a gradual shift of research paradigm from materials synthesis and characterization toward materials engineering and fine‐tuning. The observed phenomenon was ascribed to the limitations of synthesized materials as well as certain electrocatalytic related structural drawbacks [213]. For example, aggregation and restacking of g‐C_3_N_4_ nanosheets were attributed to the interlayer Van der Waals (VDWs) interaction and π─π stacking, which eventually restricted the catalytic active sites [214]. In this regard, structural engineering of g‐C_3_N_4_ offers viable answers to address the above issues.

**FIGURE 10:**
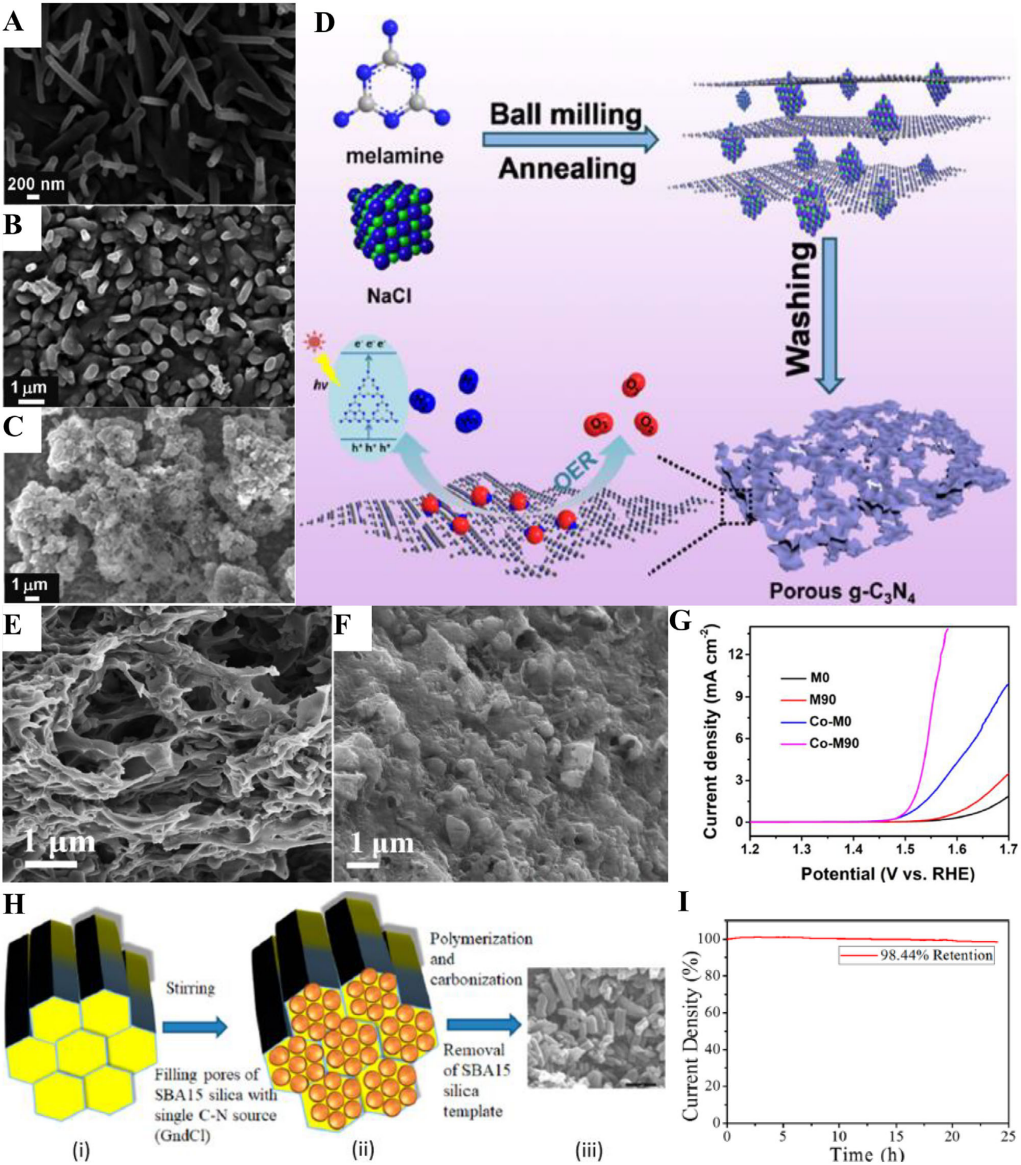
SEM images of g‐C_3_N_4_ deposited on (A) Glass, (B) FTO, and (C) TiO_2_. (Reproduced with permission [215]. Copyright 2014, Wiley‐VCH GmbH. (D) Schematic illustration of the salt‐assisted synthesis procedure of 3D porous g‐C_3_N_4_. SEM images of (E) M90 and (F) Co‐M90. (G) OER polarization curves of M0, M90, Co‐M0, Co‐M90. Reproduced with permission. Reproduced with permission [216]. Copyright 2019, American Chemical Society. (H) Schematic illustration of the nanoconfined‐mediated synthesis of gMesoCN. (i) Chronoamperometric analysis of gMesoCN for 24 h. Reproduced with permission. [217] Copyright 2020, American Chemical Society.

Alkaline HER was known for its slow reaction due to the scarce amounts of protons available in the electrolyte. Herein, Shalom et al. grew highly ordered g‐C_3_N_4_ on different substrates (glass, fluorine‐doped tin oxide (FTO), and TiO_2_) from cyanuric acid melamine (CM) complex, which fostered g‐C_3_N_4_ with different morphologies as shown in Figure 10A–C [215]. It was observed that the structures grown on glass and FTO had a homogenous and relatively organized nanorod structures, while those of TiO_2_ formed a thin g‐C_3_N_4_ layer surrounding the TiO_2_ molecules. HER electrocatalytic activities of C_3_N_4_/FTO nanorods and C_3_N_4_/TiO_2_ nanolayers were experimented in 0.1 m phosphate buffer solution, PBS (pH 6.9) and 0.1 m KOH solution (pH 13.1) where both electrocatalysts manifested similar outcomes. C_3_N_4_/FTO acquired a lower overpotential value of 300 mV in alkaline media compared to neutral media of 600 mV at the same current density of 0.8 mA cm^−2^; C_3_N_4_/TiO_2_ acquired a higher current density 1.3 mA cm^−2^ at 300 mV in alkaline electrolyte and only 1.0 mA cm^−2^ at a massive 600 mV overpotential value in neutral electrolyte. To recap, both C_3_N_4_/ TiO_2_ and C_3_N_4_/FTO functioned better in alkaline media but they were not stable under acidic conditions. In the electrochemical stability test via 500 cycles of CV with a scan rate of 25 mV s^−1^, C_3_N_4_/FTO experienced a full degradation of material shown evidently by the distinction of the fifth (black) and five hundredth (red) scan of the CV curves. As opposed to the degraded electrocatalyst, C_3_N_4_/TiO_2_ only suffered a 15% reduction in current density. All things considered, structural engineering g‐C_3_N_4_ with metallic species showed a better stability than non‐metallic based g‐C_3_N_4_ electrocatalyst.

A 3D porous‐rich g‐C_3_N_4_ electrocatalyst was synthesized through NaCl assisted ball‐milling strategy (Figure 10D). In this scenario, NaCl cubic particles acted as both the template for porous g‐C_3_N_4_ synthesis and concomitantly hindered the aggregation of g‐C_3_N_4_ during the calcination process, the samples were labeled as M0 and M90 which represented melamine to NaCl mass ratio of 1:0 and 1:90, respectively. Next, the as‐synthesized electrocatalyst was doped with Co (Co) atoms with the intention to amplify the intrinsic activity, the samples were denoted as Co‐M0 and Co‐M90, accordingly. As portrayed in Figure 10E,F, Co doping had modulated the morphology of the catalyst's structure by collapsing the pores partially, which was proven by the specific surface area decrement of M90 at 73.3 to 58.3 m^2^ g^−1^. In spite of the partial closure of the pores, the salt‐assisted Co‐M90 still overwhelmed the non‐salt‐assisted M0 with a specific surface area of merely 5.3 m^2^ g^−1^. This showed that the salt‐assisted synthetic procedure had generated a tremendous number of pores in the 3D g‐C_3_N_4_ structure. In electrocatalytic OER, Co‐M90 recorded a small onset potential of 1.47 V versus RHE (Figure 10G), $\eta_{10}$ of 320 mV, and lowest Tafel slope of 64.2 mV dec^−1^ compared with those of Co‐Mo (74.5 mV dec^−1^), M90 (100.4 mV dec^−1^), and M0 (148.8 mV dec^−1^) in the alkaline media of 0.1 m KOH. Clearly, the remarkable OER electrocatalytic activity of Co‐M90 as an OER electrocatalyst showed great prospect in HER as the sluggish kinetics of OER PCET steps were tackled [216].

In another alkaline OER experiment, Wahab et al. synthesized a mesoporous g‐C_3_N_4_ with hexagonal pores (gMesoCN) via nanoconfined‐mediated synthetic protocol using SBA15 silica nanotemplate (Figure 10H). [217] On account of the organized mesoporous structure as well as the presence of pyridinic N and graphitic N, the onset potential of gMesoCN in 0.1 and 1.0 m KOH were 1.545 and 1.514 V, accordingly with respect to RHE while the non‐templated g‐C_3_N_4_ (nTCN) marked a higher value at 1.550 V in 1.0 m KOH. A noticeable upgrade of the gMesoCN was observed when gMesoCN in 0.1 m KOH performed better than nTCN in 1.0 m KOH, which was mainly attributed to the increased accessibility to the active sites by the hexagonal mesopores. The synergistic effects between nitrogen content of gMesoCN and its mesoporous structure were realized when the pyridinic N and graphitic N boosted the number of catalytic active sites while the mesopores of gMesoCN (4.56 nm) enabled the rapid diffusion of electrolyte ions into the inner surface of the electrocatalyst [218], which escalated the OER process. Figure 10I was the chronoamperometric response of gMesoCN in 1 m KOH for 24 h, the electrocatalyst experienced an attenuation of only 1.56%, which reflected its promising structural and electrocatalytic stability. Altogether, the existence of porous g‐C_3_N_4_ structure is a unique feature that can be regarded as the removal of tiny circular invisible walls, allowing more interaction between the inner channels of the electrocatalyst and the intermediate substrates floating around the electrolyte environment.

**FIGURE 11:**
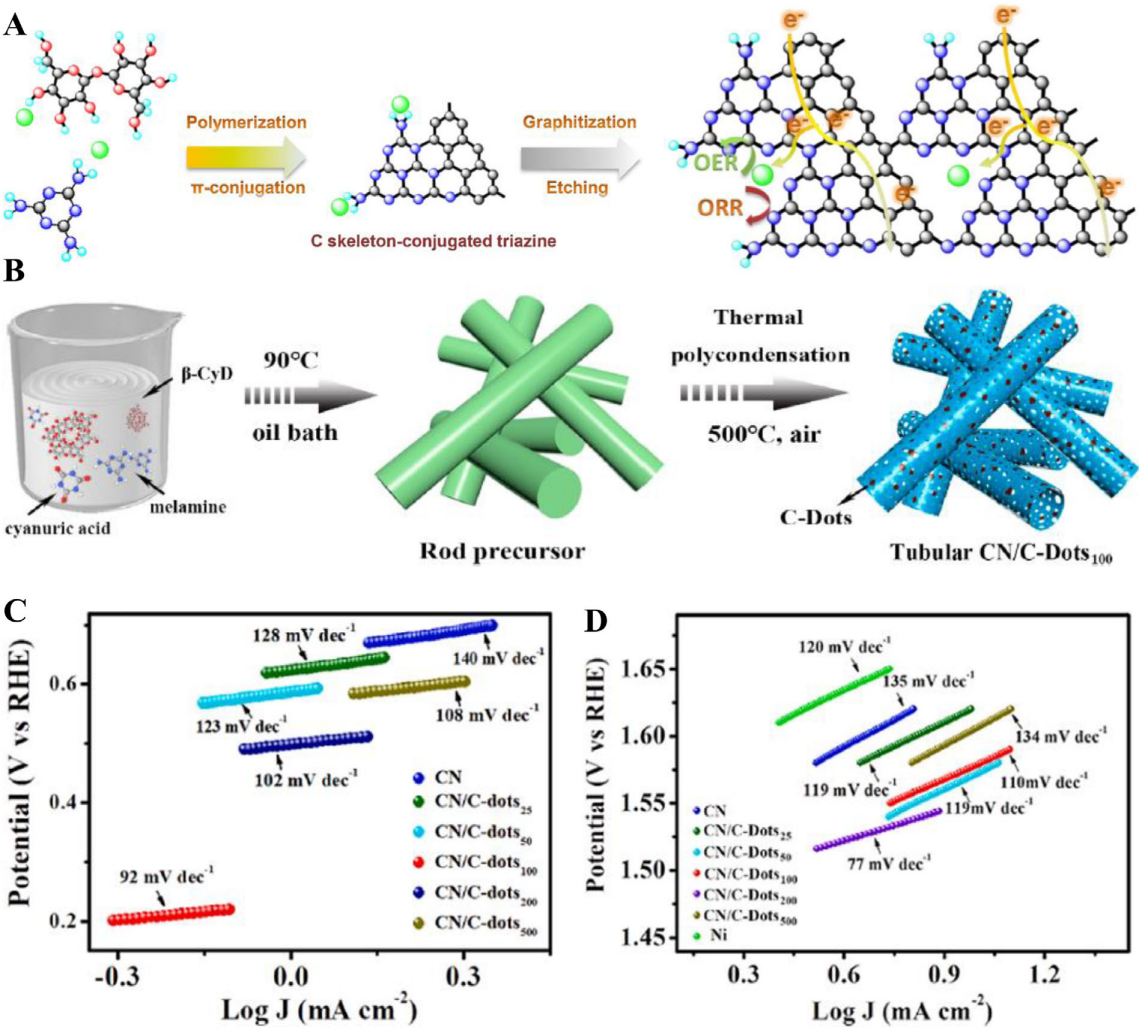
(A) Schematic representation of the synthetic steps of Co‐C_3_N_4_/C. Reproduced with permission [220]. Copyright 2018, American Chemical Society. (B) Schematic illustration of the preparation of ultra‐thin porous tubular CN/C‐Dots_100_ LHSs. (C) HER and (D) OER Tafel plots of pure CN and CN/C‐Dots*_x_* electrocatalyst. Reproduced with permission [224]. Copyright 2020, Elsevier Inc.

The idea of combining conductive carbons and g‐C_3_N_4_ for energy conversion is highly desired by virtue of its superiority [219], but is constantly limited by the complex synthetic procedures to simultaneous grow the carbon skeletons and g‐C_3_N_4_ structures. Niu et al. documented the novel solution to the aforementioned issue by in‐plane conjugation of g‐C_3_N_4_ nanosheets with crystalline carbons by using lactose as a carbon source and NaCl as salt template, Co atoms were doped as catalyst promoters (Figure 11A), the overall composite was denoted as Co‐C_3_N_4_/C [220]. As discussed previously, pyridinic N had played a major role in fostering the electrocatalytic active sites [217]. Therefore, Co atoms were doped to augment the pyridinic N contents, which was proven by the XPS N 1s spectra where the pyridinic N content rose from 3.4 at. % (C_3_N_4_/C) to 4.0% at. % (Co‐C_3_N_4_/C). The refined Co‐C_3_N_4_/C electrocatalyst had produced an OER electrochemical statistic of 1.65 V and 53 mV dec^−1^ for $\eta_{10}$ and Tafel slope, accordingly, which were comparable to those of commercial RuO_2_ electrocatalyst at 1.58 V and 51 mV dec^−1^, respectively in 0.1 m KOH electrolyte.

Principally, lateral heterostructures (LHs) with in‐plane chemical bonding at the interface between two species opened up more active sites rather than vertical heterostructures (VHs) that were formed by VDWs stacking of monolayers [221, 222, 223]. In HER and OER electrocatalysis, Li et al. developed lateral heterostructures of ultra‐thin tubular and porous g‐C_3_N_4_‐Carbon dot via thermal polymerization of melamine, cyanuric acid, and β‐cyclodextrin (Figure 11B) [224]. The as‐synthesized electrocatalyst was denoted as CN/C‐Dots*_x_* with varied amounts of β‐cyclodextrin (*x* = 25, 50, 100, 200, and 500 mg). CN/C‐Dots_100_ comprised the highest BET surface area among its peers (CN/C‐Dots_25_, CN/C‐Dots_50_, CN/C‐Dots_200_, and CN/C‐Dots_500_) at 120.4 m^2^ g^−1^ and possessed a pore volume of 0.48 cm^3^ g^−1^, which was more than twice of pure CN (0.20 cm^3^ g^−1^). Surprisingly, electrocatalytic HER (0.5 m H_2_SO_4_) and OER (1.0 m KOH) of CN/C‐Dots_100_ exhibited totally different trends (Figure 11C,D). CN/C‐Dots_100_ dominated HER activity with the lowest Tafel slope of 92 mV dec^−1^; while CN/C‐Dots_200_ was the most optimal electrocatalyst for OER. Nevertheless, all CN/C‐Dots*_x_* samples were relatively close to each other in terms performance which showed the insignificance of β‐cyclodextrin as an enhancer. Instead, β‐cyclodextrin acted more as a stabilizer for the CN/C‐Dots*_x_* composites, which was in conformity to a metal‐graphene hybrid in another electrocatalytic work [225]. The excellence of bifunctional CN/C‐Dots*_x_* electrocatalysts was mainly ascribed to the ultra‐thin and porous LHs, which substantially inhibited the agglomeration of g‐C_3_N_4_ sheets as well as leading to a larger exposed surface area, in contrast to the inefficient VHs. HER and OER were studied individually in this research, but the CN/C‐Dots*_x_* electrocatalysts had demonstrated their astonishing bifunctionality implied that they can be further employed for OWS reactions.

### 3.3 Hybridization

As we know, hybridization between different materials is a chemical engineering technique devised to overcome flaws of individual counterparts [226], as well as potentially discovering unpredictable functions from the synergistic coupling of materials like enhanced behaviors of electrocatalyst after repeated testing cycles [227]. Hence, diverse hybrids of C*_x_* N*_y_* HER electrocatalysts are discussed.

#### 3.3.1 Graphene Hybrids

Similar to C*_x_* N*_y_*, graphene also belongs to the carbon family with a single layer of carbon atoms packed in a hexagonal lattice structure, it is considered as an ideal HER hybridization candidate due to its distinguished structural merits such as great electrical conductivity, large surface area, and high chemical stability [228, 229]. The inspiration to hybridize graphene and C*_x_* N*_y_* materials originated from the persistent aspiration of researchers to create electrocatalysts that are comparable or even topping the state‐of‐the‐art noble metal‐based HER electrocatalysts.

**FIGURE 12:**
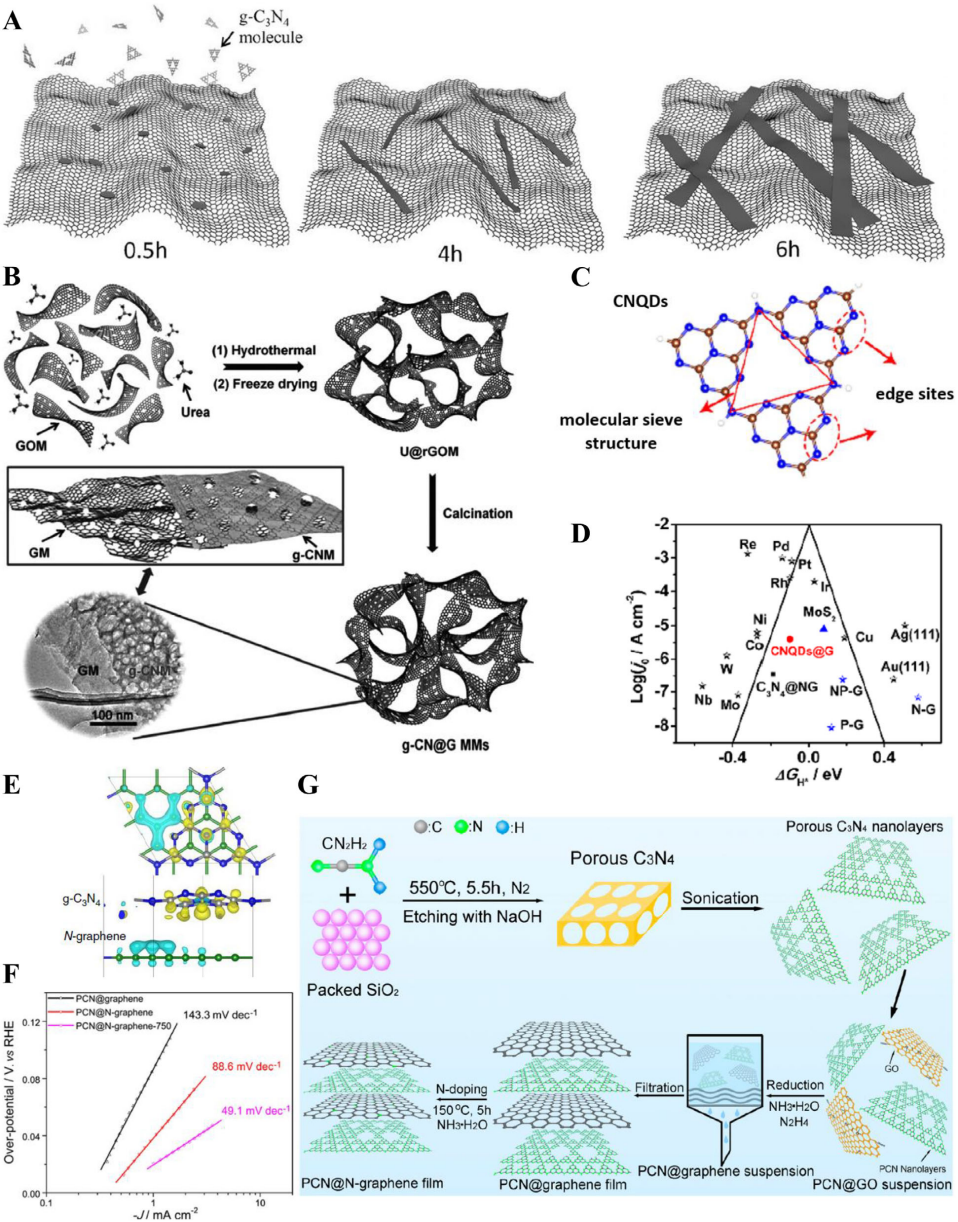
(A) Formation of g‐C_3_N_4_ nanoribbons on G hydrothermally at different reaction times. Reproduced with permission [230]. Copyright 2014, Wiley‐VCH GmbH. (B) Synthetic procedure of mesh‐on‐mesh nanoarchitecture g‐CN@G MMs. Reproduced with permission [231]. Copyright 2017, Wiley‐VCH GmbH. (C) Molecular sieve structure and edge sites of CNQDs. (D) Volcano plot of CNQDs@G and other electrocatalysts. Reproduced with permission [232]. Copyright 2018, American Chemical Society. (E) Interlayer electron transfer from conductive NG to g‐C_3_N_4_. Yellow blobs indicate electron accumulation and cyan blobs represent electron exhaustion. Reproduced with permission [234]. Copyright 2014, Springer Nature. (F) Tafel slopes of PCN with different graphene counterpart. (G) Synthetic procedure of PCN@NG film. Reproduced with permission [237]. Copyright 2015, American Chemical Society.

To begin with, the formation of 1D g‐C_3_N_4_ nanoribbons on 2D graphene sheets (g‐C_3_N_4_ nanoribbon‐G) were investigated by Zhao et al. by means of one‐step hydrothermal method up to 6 h (Figure 12A). The unique 1D nanoribbon structure was able to provide a plentiful amount of hydrogen adsorption sites and reinforce the electrical connection between g‐C_3_N_4_ and graphene, leading to an efficient electron transfer during the HER process ($\eta_{10}$ of 207 mV and Tafel slope of 54 mV dec^−1^). Interestingly, prolonged hydrothermal reaction time (24 h) could lead to the formation of nanorods, indicating the practicality of tuning g‐C_3_N_4_ nanostructures hydrothermally [230]. Likewise, construction of mesh‐on‐mesh nanostructures by a straightforward template‐free procedure (Figure 12B) involving mesoporous g‐C_3_N_4_ and mesoporous graphene (g‐CN@G MMs) provides an alternative approach to generate more HER adsorption/desorption sites, which showed an approximate electrocatalytic performance as g‐C_3_N_4_ nanoribbon‐G with $\eta_{10}$ of 219 mV and Tafel slope of 53 mV dec^−1^ [231]. Besides, g‐CN@G MMs also unveiled a higher thermal stability up to 750°C in comparison to that of g‐C_3_N_4_ nanoribbon‐G (650°C) owing to the strong interlayer coupling of the mesh‐on‐mesh nanostructures. Furthermore, downsizing the bulk g‐C_3_N_4_ and subsequent hybridization with conductive G can effectively increase the active sites and electron transfer. Zhong et al. had presented a magnificent HER performance ($\eta_{10}$ of 110 mV and Tafel slope of 53 mV dec^−1^) of a graphene supported g‐C_3_N_4_ quantum dots hybrid electrocatalyst denoted by CNQDs@G (synthesized via ultra‐sonification of bulk g‐C_3_N_4_ and subsequent hydrothermal combination with G sheets) [232]. The obtained results were attributed to its bisynergistic effects: (1) between CNQDs (electrocatalytic layer) and G (conductive layer) and (2) the molecular sieve structure and edge sites (Figure 12C). Apparently, the ∆*G*(H_ads_) for pyridinic N at edge sites was smaller than that of pyridinic N in the molecular sieve, which implied the transfer of active sites from the internal molecular sieve defects to the active edge sites. Being a non‐metal HER electrocatalysts, CNQDs@G's exceptional performance was emphasized in the volcano plot (Figure 12D) and even outperformed some of the metallic elements. Conclusively, relatively low Tafel slopes (53–54 mV dec^−1^) were observed among g‐C_3_N_4_ nanoribbon‐G, g‐CN@G MMs, and CNQDs@G, hinting a highly favorable HER reaction kinetic via Volmer–Heyrovsky pathway with hydrogen desorption being the RDS.

Even though pristine graphene hybrids were able to satisfy the development of upgraded HER electrocatalysts, innovation by researchers had brought upon the modified graphene hybrids such as N‐doped graphene [233]. In particular, coupling g‐C_3_N_4_ and N‐doped graphene (C_3_N_4_@NG) had allowed facile electrons transition from N‐doped graphene to g‐C_3_N_4_ forming an electron‐rich electrocatalytic g‐C_3_N_4_ layer (Figure 12E) and contributed towards effective charge transfer during the HER process [234]. In this research, HER process was carried out via Volmer–Heyrovsky mechanism as C_3_N_4_@NG had produced a Tafel slope of 51.5 mV dec^−1^ and a moderately low $\eta_{10}$ with 240 mV, which showed signs of similarity in terms of reaction kinetic with N‐free graphene hybrids (g‐C_3_N_4_ nanoribbon‐G, g‐CN@G MMs and CNQDs@G) as discussed previously. Evidently, porous structures have been functioning to promote the HER activities of electrocatalysts regardless of the type of materials or electrocatalysts [235], which is a positive result of its large surface area, low charge resistance, and facilitated adsorption and desorption process. [236] In relation to this topic, porous g‐C_3_N_4_ integrated with NG (PCN@NG) constructed through vacuum filtration method had exhibited a lower $\eta_{10}$ (compared to previously highlighted pristine g‐C_3_N_4_@NG) at 170 mV owing to the in‐plane macropores of the PCN nanolayers which exposed much more HER active sites. Surprisingly, Duan et al. discovered that PCN@NG after 750 cyclic voltammetry (PCN@NG‐750) showed the most optimal HER performance with $\eta_{10}$ of 80 mV and Tafel slope of 49.1 mV dec^−1^, surpassing both PCN@G and PCN@NG (Figure 12F) owing to the interactions between active species and electrocatalyst during the potential cycles [237]. Obviously, the upgraded electrocatalytic activity of PCN@NG‐750 in contrast to N‐free graphene hybrids (g‐C_3_N_4_ nanoribbon‐G, g‐CN@G MMs, and CNQDs@G) was due to the presence of N‐doped atoms on the graphene nanolayer as N atoms were able to enrich the active sites and boost the electron transfer behavior by virtue of its electronegativity [238, 239]. Overall, fascinating HER results of g‐C_3_N_4_ and N‐doped graphene hybrids spark the interest of researchers in pursuing modified hybrids in this domain. Moreover, the conversion of 3D porous g‐C_3_N_4_ to 2D porous g‐C_3_N_4_ nanolayers conducted by Duan et al. (Figure 12G) was also considered a promising method to increase the HER active sites. In short, one of the main factors in the rise of C*_x_* N*_y_* materials as electrocatalysts owes to the presence of its N atoms that directly take part in the electron transfer process.

As a candidate for hybridization, graphene had wonderfully illustrated its synergistic effects with g‐C_3_N_4_ as well as being a great support for N‐dopants. Nevertheless, a possible drawback of employing graphene during electrode synthesis includes the tendency of graphene sheets to restack that consequently minimizes the ECSA [240]. Integrating reduced graphene oxide (RGO), obtained by reducing graphene's derivative—graphene oxide (GO) is one of the most reliable ways to prevent graphene restacking [241]. RGO not only maintains the intrinsic properties of graphene like electrical conductivity by removing excess oxygen‐containing functional groups [242], but also serves as a spacer to suppress the reaggregation or restacking of MXenes, other common spacers include carbon nanotubes (CNTs) [243].

**FIGURE 13:**
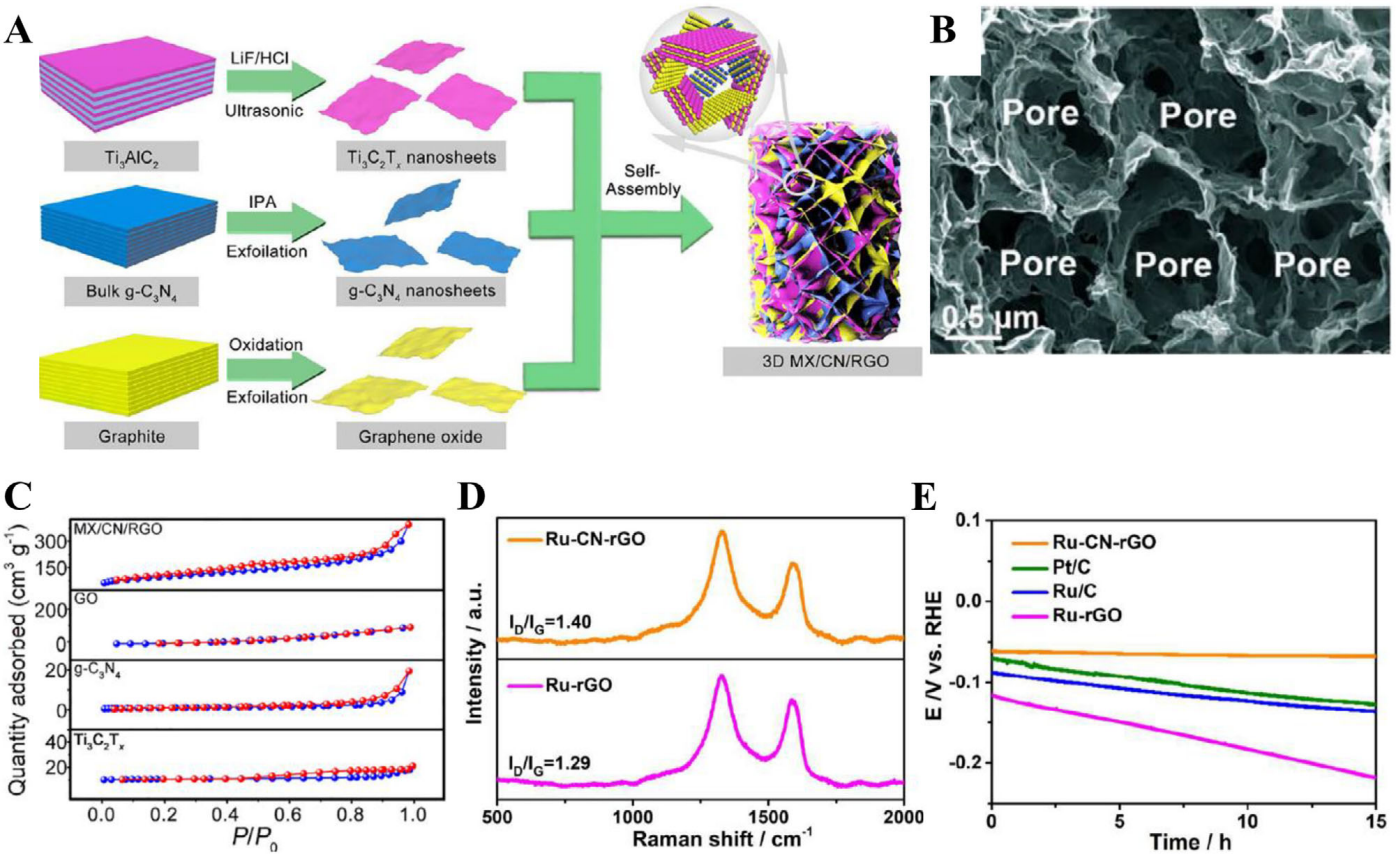
(A) Synthetic procedure of MX/CN/RGO ternary hybrid. (B) FE‐SEM of MX/CN/RGO. (C) N adsorption–desorption isotherms of MX/CN/RGO and their individual counterparts. Reproduced with permission [244]. Copyright 2022, Elsevier Inc. (D) Raman spectra of Ru‐CN‐rGO and Ru‐rGO. (E) Chronopotentiometry analysis of Ru‐Cn‐rGO, Pt/C, Ru/C, and Ru‐rGO. Reproduced with permission [247]. Copyright 2020, Wiley‐VCH GmbH.

He et al. reported a ternary HER electrocatalyst composed of Ti_3_C_2_T*_x_*, g‐C_3_N_4_, and RGO (MX/CN/RGO), formed via solvothermal co‐assembly process (Figure 13A). As depicted in Figure 13A,B, the resulting 3D MX/CN/RGO exhibited a well‐defined interweaving framework with numerous macropores ranging several hundred nm to few µm based on field emission scanning electron microscopy (FE‐SEM) analysis. The BET surface area (measured through N adsorption‐desorption isotherm) of MX/CN/RGO interweaved nanostructure reached 345.6 m^2^ g^−1^, while pristine Ti_3_C_2_T*_x_*, g‐C_3_N_4_, and GO nanosheets only recorded BET surface areas of only 12.2 m^2^ g^−1^, 4.0 m^2^ g^−1^, and 11.2 m^2^ g^−1^ respectively (Figure 13C). The stark variance of BET surface areas was accredited to the severe reaggregation and restacking of pristine Ti_3_C_2_T*_x_* and GO nanosheets, which was suppressed when the ternary interweaving MX/CN/RGO nanoarchitecture was formed. The corresponding pore size ranging from 2 to 40 nm provided significant amount of accessible active sites also contributed to the huge deviation of BET surface areas. Taking MX/CN/RGO as an example, RGO had beautifully acted as a spacer in preventing the reaggregation of Ti_3_C_2_T*_x_* as noted earlier [243]. Hence, the advanced MX/CN/RGO HER electrocatalyst generated a satisfyingly low onset potential at 38 mV (with $\eta_{10}$ of 148 mV and Tafel slope of 76 mV dec^−1^) in 0.5 m H_2_SO_4_ solution [244], which was superior to the state‐of‐the‐art g‐C_3_N_4_‐based electrocatalysts (g‐C3N4/B‐doped RGO at 80 mV and MoS_2_‐C_3_N_4_/RGO at 140 mV) [245, 246].

Generally, researchers strive to obtain a pH universal electrocatalyst as it can tolerate wide range of aqueous solutions with sustained catalytic stability. HER usually thrives under acidic condition due to the abundance of protons, but electrocatalyst performing under alkaline conditions may as well provide similar capabilities. In contrary to the previous work [244], Wang et al. had hydrothermally synthesized ruthenium‐g‐C_3_N_4_‐reduced graphene oxide (Ru‐CN‐rGO) ternary composite, which was a robust HER electrocatalyst under alkaline media (1.0 m KOH) [247]. The rise of the D/G bands intensity ratio from 1.29 to 1.40 through Raman spectroscopy (Figure 13D) implied the increase of disordered carbon structures and defects [248], which was attributed to the strong interaction effects of Ru, CN, and rGO. Moreover, this electrocatalyst had superb electrocatalytic durability up to 15 h of chronopotentiometry testing (Figure 13E) thanks to the presence of g‐C_3_N_4_ that acted as a glue to firmly gripping Ru nanoparticles. This allowed Ru‐CN‐rGO to achieve better HER results than MX/CN/RGO with $\eta_{10}$ of 45 mV and Tafel slope of 40 mV dec^−1^. Overall, g‐C_3_N_4_ hybrids with pristine G (g‐C_3_N_4_ nanoribbon‐G, g‐CN@G MMs, and CNQDs@G; Tafel slope range of 53–54 mV dec^−1^), modified G (C_3_N_4_@NG and PCN@NG; Tafel slope of 51.5 and 49.1 mV dec^−1^), as well as ternary RGO (MX/CN/RGO and Ru‐CN‐rGO; Tafel slope of 76 and 40 mV dec^−1^) revealed uniform HER reaction kinetic through the Volmer–Heyrovsky mechanism. This also means that hydrogen desorption remains as one of the biggest hurdles in achieving the ideal electrocatalyst.

#### 3.3.2 Transition Metal (TM) Hybrids

**FIGURE 14:**
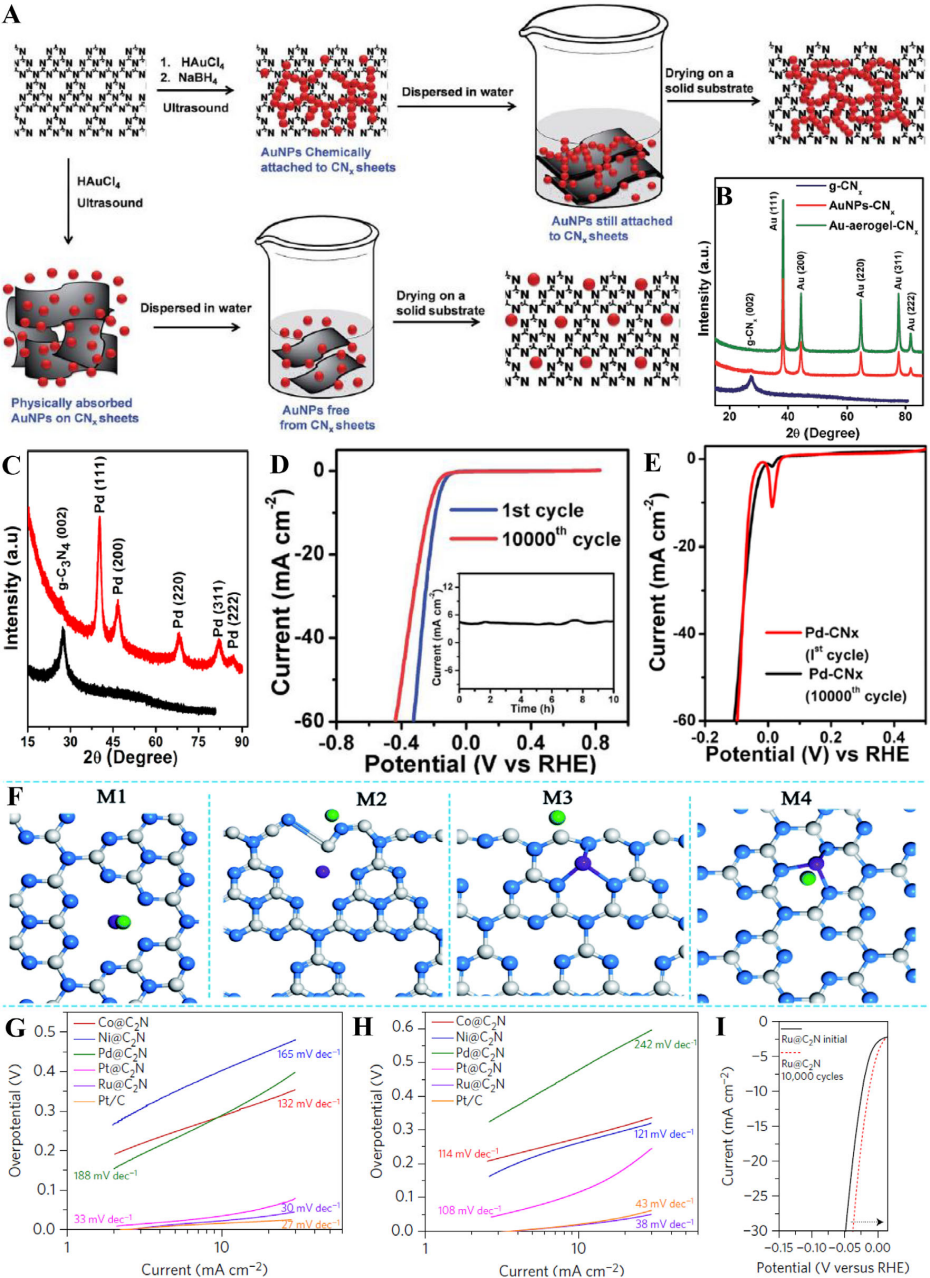
(A) Synthetic illustration of ultrasound‐mediated formation of Au‐based composites. p‐XRD patterns for (B) g‐CN*_x_*, AuNPs‐CN*_x_*, Au‐aerogel‐CN*_x_* and (C) g‐C_3_N_4_ (black line) and Pd‐CN*_x_* (red line). LSV curves of (D) Au‐aerogel‐CN*_x_* and (E) Pd‐CN*_x_* at the first and 10,000th cycle. Reproduced with permissions [251, 252]. Copyright 2016, American Chemical Society. Copyright 2015, Royal Society of Chemistry. (F) DFT optimized structures of M1, M2, M3, and M4 with H atom adsorbed at different positions. M1 and M2 represents tri‐*s*‐triazine while M3 and M4 illustrates triazine structures of g‐C_3_N_4_; H atom was adsorbed in the plane of M1 and M4 while at the edge sites of M2 and M3. The white, blue, purple, and green balls stand for C, N, Ru, and H atoms, respectively. Reproduced with permission [254]. Copyright 2021, Royal Society of Chemistry. Tafel plots of Co@C_2_N, Ni@C_2_N, Pd@C_2_N, Pt@C_2_N, Ru@C_2_N, Pt/C in (G) 0.5 m H_2_SO_4_ and (H) 1.0 m KOH. (I) LSV curves of Ru@C_2_N at the first and 10,000th cycle. Reproduced with permission [255]. Copyright 2017, Springer Nature.

In view of the success and dominance of Pt/C in the electrocatalysis scene, Barman's group had probed into other noble metal based electrocatalysts by a simple ultrasound‐mediated synthetic method as portrayed in Figure 14A. They successfully developed gold aerogel, gold nanoparticles, and palladium nanoparticles supported on g‐C_3_N_4_ (Au‐aerogel‐CN*_x_*, AuNPs‐CN*_x_*, and Pd‐CN*_x_*, accordingly) via the aforementioned method. Aside from its catalytic properties, the commercial Pt/C had displayed poor durability in highly oxidizing conditions [249]. Therefore, Barman's group intended to search for an electrocatalyst with comparable performance but better durability than that of Pt/C for electrocatalytic purposes. The intense and sharp diffraction peaks of Au and Pd were observed in their respective powder‐XRD (p‐XRD) patterns (Figure 14B,C), which indicated that the Au aerogels, Au and Pd nanoparticles were formed in a highly crystalline manner uniformly around g‐C_3_N_4_ that diminished the signal of the host material [250]. The doubts of Au and Pd being dopant materials were also clarified simultaneously via their p‐XRD patterns where different phases were observed rather than the slight shifting of single phase diffraction peaks of doped g‐C_3_N_4_ in Figure 14E,B. $\eta_{10}$ and Tafel slopes of Au‐aerogel‐CN*_x_*, AuNPs‐CN*_x_*, and Pd‐CN*_x_* were 185 mV, 285 mV, and 55 mV, respectively and 53, 72, and 35 mV dec^−1^, accordingly under acidic conditions (0.5 m H_2_SO_4_). The as‐synthesized Au‐aerogel‐CN*_x_* and Pd‐CN*_x_* exhibited superb electrochemical stability after 10,000 cycles (Figure 14D,E), which were highly promising in serving as Pt/C's replacements. In both researches, the porous network of the composites (Au‐aerogel‐CN*_x_*, AuNPs‐CN*_x_*, and Pd‐CN*_x_*) induced during the ultrasound‐mediated synthesis was responsible for their magnificent electrocatalytic performance; while the stability of the electrocatalysts was thanks to the strong chemical interaction of Au and Pd atoms with the g‐C_3_N_4_ structures, which provided sufficient mechanical adhesion to withstand harsh conditions [251, 252]. Evidently, g‐C_3_N_4_ is more competent than carbon supports used in Pt/C when it comes to electrocatalysis.

Pt, Pd, Au and other noble metal‐based electrocatalysts were mostly limited in large scale industrial applications appertaining to their high cost and scarcity. As such, non‐precious transition metals were sourced as an alternative. Among candidate materials, Ru expressed hydrogen bonding strength close to that of Pt and was believed to be a reassuring alternative [253]. Li et al. effectively merged Ru^3+^ and g‐C_3_N_4_ to generate Ru/g‐C_3_N_4_ hybrid with varying average crystal size Ru/g‐C_3_N_4_‐1 (3.74 nm), Ru/g‐C_3_N_4_‐2 (5.38 nm), and Ru/g‐C_3_N_4_‐3 (24.93 nm) via pyrolysis of RuCl_3_ and melamine, larger average crystal size was achieved by increasing the concentration of Ru precursor RuCl_3_ [254]. High‐resolution XPS analysis demonstrated that a strong Ru─N coupling was formed when the C─N═C relative content dropped from 90% (pristine g‐C_3_N_4_) to 2% (Ru/g‐C_3_N_4_‐2), which was predominantly caused by the C─N═C bond breakage and Ru─N bond linkage. Following the Ru‐N coupling, DFT studies were carried out focusing on the Ru─N coordination by simplifying possible Ru/g‐C_3_N_4_ models labeled as M1, M2, M3, and M4 (Figure 14F) and the lowest energy barrier of H adsorption was 0.12 eV achieved by M4, which was exceptionally smaller than others according to the simulated free‐energy profiles. In M4, Ru atoms acted as the active sites and were mediated by the surrounding N atoms of g‐C_3_N_4_. The projected electronic density of states (PDOS) unveiled that s, p, and d orbitals Ru were well‐matched with the neighboring N orbitals near the Fermi level, which were an obvious indicative that Ru d orbitals were involved in the formation of Ru─N bonds while the Ru s and p orbitals only involved in partial orbital hybridization with the nearest N atoms. The intimate interaction between Ru and g‐C_3_N_4_ in the orbital level established a favorable electron transfer and H adsorption that guaranteed an enhanced HER activity. The most optimal as‐synthesized hybrid (Ru/g‐C_3_N_4_‐2) corresponded to the theoretical analysis superbly by achieving a marvelous $\eta_{10}$ and Tafel slope results of 27 mV and 22 mV dec^−1^, respectively in 0.5 m H_2_SO_4_ and 34 mV and 27 mV dec^−1^, accordingly in 1.0 m KOH, better than Pt/C in both pH conditions (31 mV and 27 mV dec^−1^ in 0.5 m H_2_SO_4_; 49 mV and 34 mV dec^−1^ in 1.0 m KOH) [254]. In conclusion, N atoms of pristine g‐C_3_N_4_ usually serve as the active sites for H adsorptions, nevertheless, they interact wonderfully with other species such as transition metals during hybridization by forming chemical bonds and opening the passage for systematic electron transfers.

Through ultrasound‐mediated methodologies similar to that of Barman's group [251, 252], Mahmood et al. analyzed the HER performance of different noble and non‐precious metal atoms (Pt, Pd, Ru, Co, Ni) hybridized with C_2_N, which is an allotrope of g‐C_3_N_4_. The C_2_N framework was formed by reacting hexaaminobenzene and hexaketocyclohexane in *N*‐methyl‐2‐pyrrolidone (NMP) along with NaBH_4_ and other metal precursors under 175°C. Ru@C_2_N was hailed as the most efficient electrocatalyst among studied samples (Co@C_2_N, Ni@C_2_N, Pd@C_2_N, Pt@C_2_N, Ru@C_2_N) in both acidic (Figure 14G) and alkaline (Figure 14H) solutions. Evidently, Ru@C_2_N achieved the best results in both pH conditions with an extraordinary stability after 10,000 cycles of testing (Figure 14I). Interestingly, the exceptional behavior of Ru@C_2_N in 0.5 m H_2_SO_4_ reached Tafel slope of 30 mV dec^−1^ suggested that the H_2_ production pathway was executed via Volmer–Tafel mechanism in which the recombination of adsorbed H atoms was the rate‐limiting step. This achievement of Ru@C_2_N was in conformity with Ru/g‐C_3_N_4_‐2 (Tafel slope 22 mV dec^−1^) [254] mentioned earlier. Also, HER kinetics of Ru@C_2_N and Ru/g‐C_3_N_4_‐2 marked the difference with other metal‐based electrocatalysts including Co@C_2_N (132 mV dec^−1^), Ni@C_2_N (165 mV dec^−1^), Pd@C_2_N (188 mV dec^−1^), Pt@C_2_N (33 mV dec^−1^) in this work [255], as well as Au‐aerogel‐CN*_x_* (53 mV dec^−1^), AuNPs‐CN*_x_* (72 mV dec^−1^), Pd‐CN*_x_* (35 mV dec^−1^) from Barman's group [251, 252], which proceeded through the Volmer–Heyrovsky mechanism where the RDS belongs to the desorption of H_2_ from the electrocatalyst. In conclusion, despite the distinct framework, C_2_N and g‐C_3_N_4_ provided matching characteristics as hybrid materials and the escalated catalytic activities were contributed from their hybrid counterparts.

**FIGURE 15:**
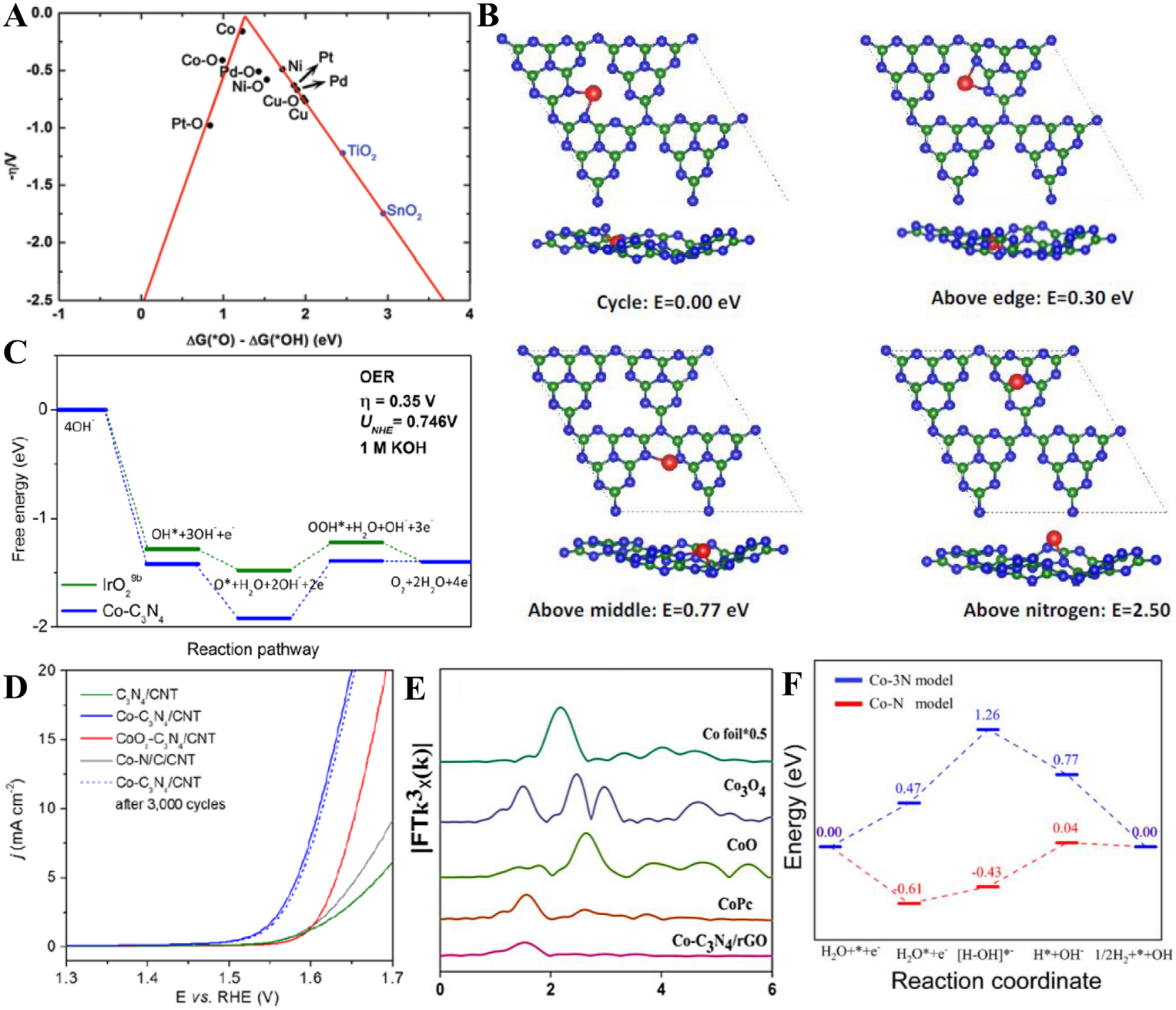
(A) Volcano plots of TM and TM‐O group with OER *η* as a function of ∆*G*(O_ads_)—∆*G*(OH_ads_), which is the Gibbs free energies difference between O_ads_ and OH_ads_ catalytic steps. (B) Top and side views of optimized Co location in different coordination sites of the g‐C_3_N_4_ framework. (C) Calculated free energy diagram for Co‐C_3_N_4_ and benchmark IrO_2_ in OER elementary steps. (D) Polarization curves of Co‐C_3_N_4_ and other electrocatalysts. Reproduced with permission [258]. Copyright 2017, American Chemical Society. (E) Co K‐edge FT k^3^‐weighted EXAFS in *R* space. (F) Relative energy profiles for Co‐3N and Co‐N models. Reproduced with permission [261]. Copyright 2022, American Chemical Society.

Generally, the sp^2^ bonded N atoms of g‐C_3_N_4_ provide coordination sites for TM leading to the formation of strong covalent TM‐N coordinated bonds, which prohibit the TM atoms from aggregating. The electron‐rich N atoms also induce effective polarized charges on the bonded TM that speeds up the HER and OER process [256]. In a first‐principles simulations work, Li et al. conducted DFT calculations on TM coordinated hybrids between Pt, Pd, Co, Ni, Cu and g‐C_3_N_4_ (CN) for OER under alkaline environment. Interestingly, the adsorption of OER intermediates like O on TM@CN produced stable TM‐O@CN structure, which facilitated the OER electrocatalytic activity. As depicted in Figure 15A, Co@CN and Co‐O@CN were listed as the most ideal electrocatalysts among their peers with an exceptionally low *η* of 160 mV and 410 mV, respectively. The Gibbs free energies for OER's four elementary steps were computed for Co@CN and Co‐O@CN as Co and Co‐O group provided and open site for the binding of intermediates. Firstly, Co@CN only needed 0.64 eV in the first reaction step of water dissociation, followed by the adsorption of O_ads_ which required 0.82 eV. Thirdly, the formation of OOH_ads_ on the electrocatalyst surface required 0.98 eV, which turned out to be the highest among other steps and hailed as the RDS in this Co@CN catalyzed OER activity. Finally, the discharge of H^+^ and e^−^ from the OOH_ads_, as well as the desorption of O_2_ consumed 0.82 eV. Obviously, the Co redox reaction induced by the local Co coordination was strongly involved in the OER elementary steps. On the other hand, Co─O@CN recorded Gibbs free energies of 0.83, 0.58, 1.23, 0.62 eV, respectively where the RDS fell on the formation of OOH_ads_ equivalent to that of Co@CN [257]. From this computational study, the local coordination of TM atoms and their subsequent intermediate groups (TM‐O) in OER was proven to be promising electrocatalysts and they behave similarly in catalytic activity.

In view of the success of Co atoms coordination work performed by Li et al. [257], subsequent researches on the coordination of Co atoms on g‐C_3_N_4_ at different level were carried out. Molecular‐level g‐C_3_N_4_ coordinated TM atoms were studied by Zheng et al. theoretically and experimentally confirmed that the remarkable OER electrocatalytic activity of Co‐C_3_N_4_ originated from the precise Co‐N_2_ coordination moiety in the g‐C_3_N_4_ matrix. Based on DFT calculations, the most stable site of Co belonged in the center of the tri‐*s*‐triazine ring bonded to two adjacent pyridinic N atoms with a binding energy of 0 eV (Figure 15B). The theoretical free energy diagram for Co‐C_3_N_4_ and benchmark IrO_2_ catalyst elucidated that the OER rate‐limiting step was the formation of OOH*, which was similar to the previous work (Figure 15C). In their experimental work, CNT was applied to the Co‐C_3_N_4_ as a catalyst support, yielding Co‐C_3_N_4_/CNT that possessed higher performance than other electrocatalysts as depicted in Figure 15D. This Co‐N_2_ coordination bond also granted outstanding stability with minimal shifting of the polarization curve after 3000 cycles in 1.0 m KOH solution [258]. In short, coordination engineering of g‐C_3_N_4_ with transition metals had shown great potential in tackling the sluggish PCET steps of OER. On a different note, Co was proven to be a unique transition metal among the rest as it is one of the few elements that is naturally magnetic and maintains its magnetism at high temperature [259], this feature was explored by Hunt et al. previously to determine the relationship between magnetic flux density and OER activity at the surface of the magnetic responsive CoO*_x_*/FTO anode [260]. However, the research of g‐C_3_N_4_ coordinated Co atoms in OWS is still lacking.

Indeed, the electron‐rich N coordinators on g‐C_3_N_4_ assist to bind transition metal atoms, which was examined clearly earlier [257, 258]. The capabilities of N‐coordinated Co hybrid was substantially upgraded by Liu et al. through the incorporation of reduced graphene oxide (rGO) forming Co‐g‐C_3_N_4_/rGO hybrid electrocatalyst [261]. One of the most noticeable improvements was observed from the HER electrochemical stability test performed on Co‐g‐C_3_N_4_/rGO, Co‐g‐C_3_N_4_, and Pt/C for 500 h. The instability of Co‐g‐C_3_N_4_ as opposed to Co‐g‐C_3_N_4_/rGO was attributed to the unpaired electrons in the Co atoms, which were prone to oxidation and caused the exfoliation of Co atoms (active site) from the hybrid, ultimately impacting the overall performance of the electrocatalyst [262]; conversely, the reinforced stability of Co‐g‐C_3_N_4_/rGO was thanks to the oxygen‐doped surface of rGO, which provided extensive binding energy toward metal atoms and holding them tightly in place [263]. The Co‐N coordination atomic pair was measured by Fourier transformation (FT) *k* ^3^‐weighted extended X‐ray absorption fine structure (EXAFS) and a single pinnacle at 1.5 Å was labeled as Co‐N's first coordination shell, this low coordination value also suggested that Co‐g‐C_3_N_4_/rGO possessed inferior crystal properties in contrary to CoPc protein with a crystalline structure (Figure 15E). The lowest Tafel slope of Co@N‐CNT@g‐C_3_N_4_ (88 mV dec^−1^) was highlighting dramatically improved HER kinetics. DFT calculations revealed that g‐C_3_N_4_ facilitated H_2_O dissociation through electron accumulation, while the Co nanoparticles promoted efficient H* recombination, resulting in a near‐optimal Δ*G*H* value of −0.13 eV for Co@N‐CNT@g‐C_3_N_4_, compared to g‐C_3_N_4_ and Co@CNT [264, 265]. Evidently, Co─N model provided lower energy profiles at each reaction step in contrary to Co─3N. Also, the free energy of adsorbed hydrogen (H^*^) in Co─N and Co─3N models were 0.04 and 0.77 eV, respectively, which further confirmed the lower energy barriers of Co─N in HER process (Figure 15F) [266]. These results demonstrate the pivotal role of g‐C_3_N_4_ in enhancing both the performance and durability of the Co@N‐CNT@g‐C_3_N_4_ catalyst, making it a highly promising candidate for HER applications.

**FIGURE 16:**
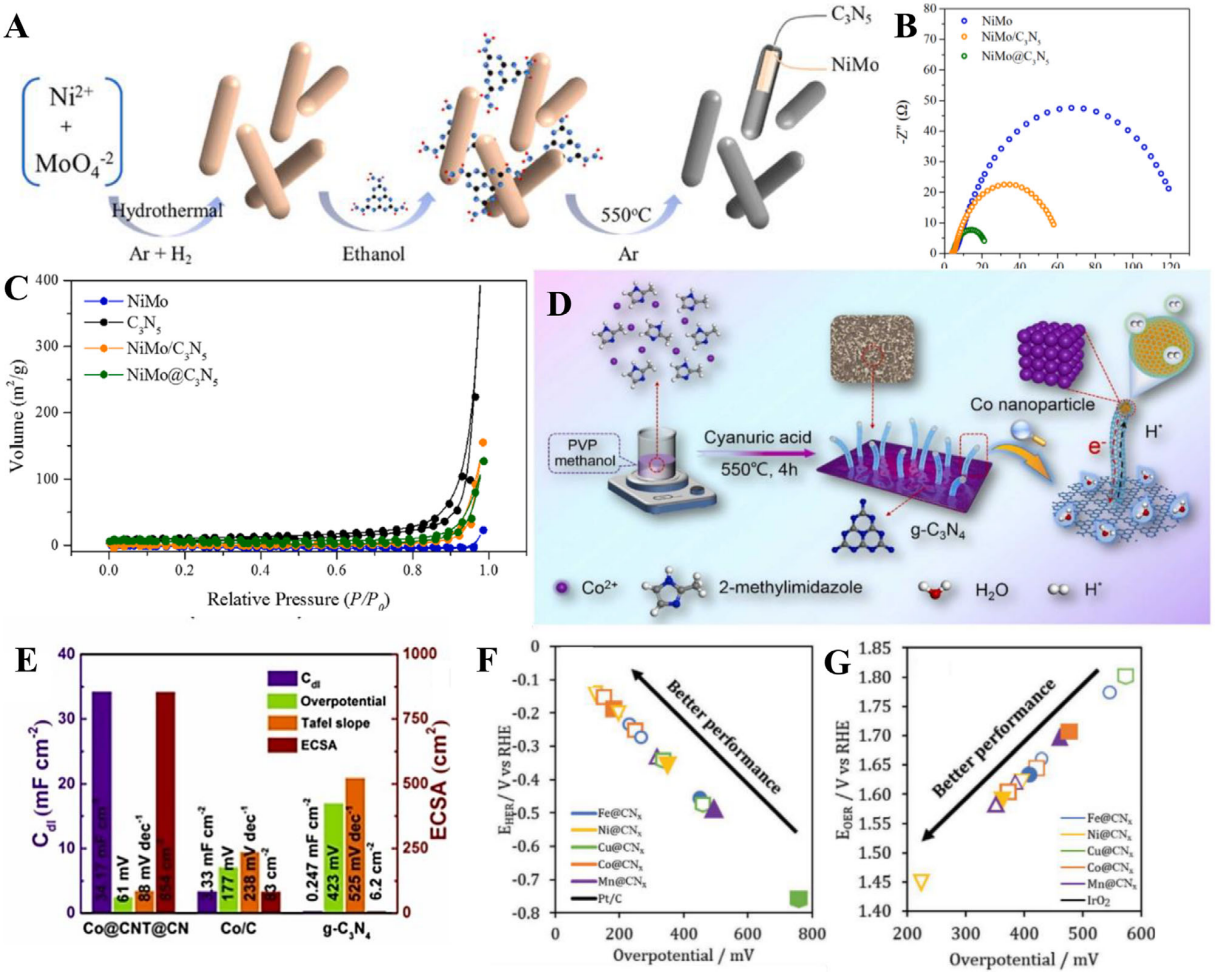
(A) Schematic illustration of the preparation of 1D@2D NiMo@C_3_N_4_ configuration. (B) Nyquist plots of NiMo, NiMo/C_3_N_5_, and NiMo@C_3_N_5_ at $\eta_{10}$ of 100 mV. (C) BET curves of C_3_N_5_, NiMo, NiMo@C_3_N_5_, and NiMo/C_3_N_5_, respectively. Reproduced with permission [268]. Copyright 2022, Elsevier Inc. (D) Schematic diagram of the preparation process of Co@CNT@CN. (E) Summary of double‐layer capacitance (*C*_dl_), overpotential, Tafel slope and electrochemically active surface areas (ECSA) by Co@CNT@CN, Co/C and g‐C_3_N_4_. Reproduced with permission [269]. Copyright 2024, Elsevier Inc. (F) Literature search of transition metal‐based electrocatalysts for the HER. Blue, yellow, orange, green, and violet represent Fe‐, Ni‐, Co‐, Cu‐, and Mn‐based catalysts, respectively. The solid symbols correspond to the data obtained in the work. (G) Literature search of transition metal‐based electrocatalysts for the OER in 1.0 m KOH solution. Blue, yellow, orange, green, and violet represent Fe‐, Ni‐, Co‐, Cu‐, and Mn‐based catalysts, respectively. The solid symbols correspond to the data obtained in the work. Reproduced with permission [273]. Copyright 2023, Wiley‐VCH GmbH.

The exploration for g‐C_3_N_4_ allotropes in water splitting raised positive results when Bu et al. formulated a heterostructure composed of 1D dual‐metal NiMo cores surrounded by 2D C_3_N_5_ shells via two‐step hydrothermal and calcination method (Figure 16A). Initially, NiMo nanorods were expected to possess the lowest *R*_ct_ value seeing that they were comprised of dual‐metallic atoms. Yet, the Nyquist plot revealed that NiMo@C_3_N_5_ core‐shell hybrid had the smallest *R*_ct_ when compared to NiMo and a mixture of both NiMo and C_3_N_5_ (NiMo/C_3_N_5_), indicated that a strong electron interaction between NiMo and C_3_N_5_ was detected (Figure 16B). Specifically, the existence an electron transfer channel was possible owing to the Mo─N covalent bond formed during hybridization, which was in line with another catalytic work [267]. After theoretically revealing the RDS was the initial water dissociation process, NiMo@C_3_N_5_ was found to reduce the energy barrier of this process from 0.96 eV (by NiMo) to 0.82 eV thanks to the porous layer of C_3_N_5_, which considerably increased the BET surface area of the overall electrocatalyst from 1.99 m^2^ g^−1^ (NiMo) to 6.37 m^2^ g^−1^ in (NiMo@C_3_N_5_) as illustrated in Figure 16C. The superiority of NiMo@C_3_N_5_ in alkaline (1.0 m KOH) HER also encouraged the authors to conduct electrocatalytic HER using natural seawater (pH value of 7.4). From the results, NiMo@C_3_N_5_ surpassed Pt/C's performance at 30th cycle hinted that the nanorods hybrid manifested a sustained stability as contrasted with Pt/C, which was ascribed to the C_3_N_5_ shells tightly wrapping around the electrocatalytic NiMo cores from the corrosion and biological contaminants incurred by the seawater [268]. In summary, a core‐shell hybrid had proven to be an amazing candidate to carry out electrocatalytic activities under harsh conditions, which is highly suitable in industrial environments.

Yan et al. have developed Co@N‐CNT@g‐C_3_N_4_, using a coordination‐polymerization integrated strategy to achieve efficient HER at all pH levels. This catalyst integrates Co nanoparticles encapsulated at the tips of nitrogen‐doped carbon nanotubes (N‐CNTs) grown on g‐C_3_N_4_, forming a closely interconnected interface that enhances electron transfer efficiency and stability (Figure 16D) [269]. Compared to Co/C and g‐C_3_N_4_ alone, Co@N‐CNT@g‐C_3_N_4_ demonstrated significantly superior performance, with overpotentials of 61, 145, and 170 mV in 1 m KOH, 0.5 m H_2_SO_4_, and 1.0 m PBS, respectively, to drive 10 mA cm^−2^. The enhancement provided by g‐C_3_N_4_ was further evident in the ECSA, where Co@N‐CNT@g‐C_3_N_4_ achieved 854cm^2^, (Figure 16E). The lowest Tafel slope of Co@N‐CNT@g‐C_3_N_4_ (88 mV dec^−1^) was highlighting dramatically improved HER kinetics. DFT calculations revealed that g‐C_3_N_4_ facilitated H_2_O dissociation through electron accumulation, while the Co nanoparticles promoted efficient H* recombination, resulting in a near‐optimal ΔGH* value of −0.13 eV for Co@N‐CNT@g‐C_3_N_4_, compared to g‐C_3_N_4_ and Co@CNT [264, 265]. These results demonstrate the pivotal role of g‐C_3_N_4_ in enhancing both the performance and durability of the Co@N‐CNT@g‐C_3_N_4_ catalyst, making it a highly promising candidate for HER applications.

Isolated transition metals (TMs) in the form of metal nanoclusters (MNs) and single‐atom catalysts (SACs) have demonstrated outstanding catalytic performance for ORR, OER, and HER [270, 271, 272]. Quílez‐Bermejo et al. explored the advanced design of MNs and SACs embedded in C_1_N_1_‐derived carbon materials for various electrochemical reactions, including the ORR, HER, and OER. By systematically embedding single atoms and nanoclusters of Fe, Ni, Co, Cu, and Mn into a C_1_N_1_‐type material with internal cavities of ≈0.6 nm, they utilized the material's four nitrogen atoms as anchoring points to create transition metal‐nitrogen (TM‐N_4_) [273]. After pyrolysis at 800°C, the resulting TM@CN*_x_* structures displayed remarkable performance across different reactions. Co@CN*_x_* showed the best HER activity with an HER of −0.27 V in alkaline media and −0.25 V in acidic media among the TM‐based electrocatalysts and nearing commercial Pt/C performance. Ni@CN*_x_* demonstrated the best kinetics among the TM@CN*_x_* samples in alkaline solution, with a Tafel slope of 120 mV dec^−1^ indicating the Volmer step. Figure 16F compares the catalytic performance of this work with other transition metal‐based catalyst for HER. In this work, the HER performance follows the order: Co > Ni > Fe > Mn > Cu in both acidic and alkaline conditions [266, 269, 274, 275, 276, 277, 278]. DFT modeling based on Co@CN*_x_* shows the low ΔGH* (0.02 eV), close to the theoretical value, is indicative of a straightforward HER and supports the high experimental catalytic performance.

Ni@CN*_x_* exhibited outstanding OER activity in alkaline media (1 m KOH), achieving an OER of 1.59 V versus RHE, significantly surpassing the performance of benchmark IrO_2_‐based catalysts (OER = 1.72 V vs. RHE). This exceptional catalytic performance can be attributed to the presence of NiN_4_ species, where the electronic coupling of Ni─N bonds lowers the Fermi level and enhances the chemisorption of OER intermediates [279]. Tafel plots revealed that Fe@CN*_x_* displayed the best kinetics among the TM@CN*_x_* materials, with a Tafel slope of 222 mV dec^−1^, outperforming the IrO_2_ catalyst (229 mV dec^−1^). The overall catalytic trend for alkaline OER follows the order: Ni > Fe > Mn > Co >> Cu. Figure 16G compares the OER performance of this work with other transition metal‐based catalysts [269, 280, 281, 282, 283, 284, 285, 286, 287]. DFT modeling based on the NiN_4_ cluster indicated that the second reduction step (OH* → O* + H+ + e−, Δ*G* = 0.95 eV) is the most endergonic stage, requiring high energy. Strategies such as dual‐site or tri‐site systems and advanced carbon supports could further enhance the HER and OER activity of TM@CN*_x_* by optimizing active site configurations and overcoming the energy barriers in the Volmer step for HER and the second reduction step for OER.

#### 3.3.3 Transition Metal Oxide (TMO) Hybrids

In addition to the hybridization of g‐C_3_N_4_ with single or dual TM atoms, transition metal oxides (TMOs) with different structures and morphologies have been reported for their electrocatalytic HER and OER activity [103, 160, 288, 289, 290, 291]. Normally, pure TMO in bulk form was believed to be inactive towards HER in the past attributing to their poor electrical conductivity [292], weak hydrogen adsorption ability, and restricted catalytic active site [293], nevertheless, they displayed better activity for the anodic OER [294, 295].

**FIGURE 17:**
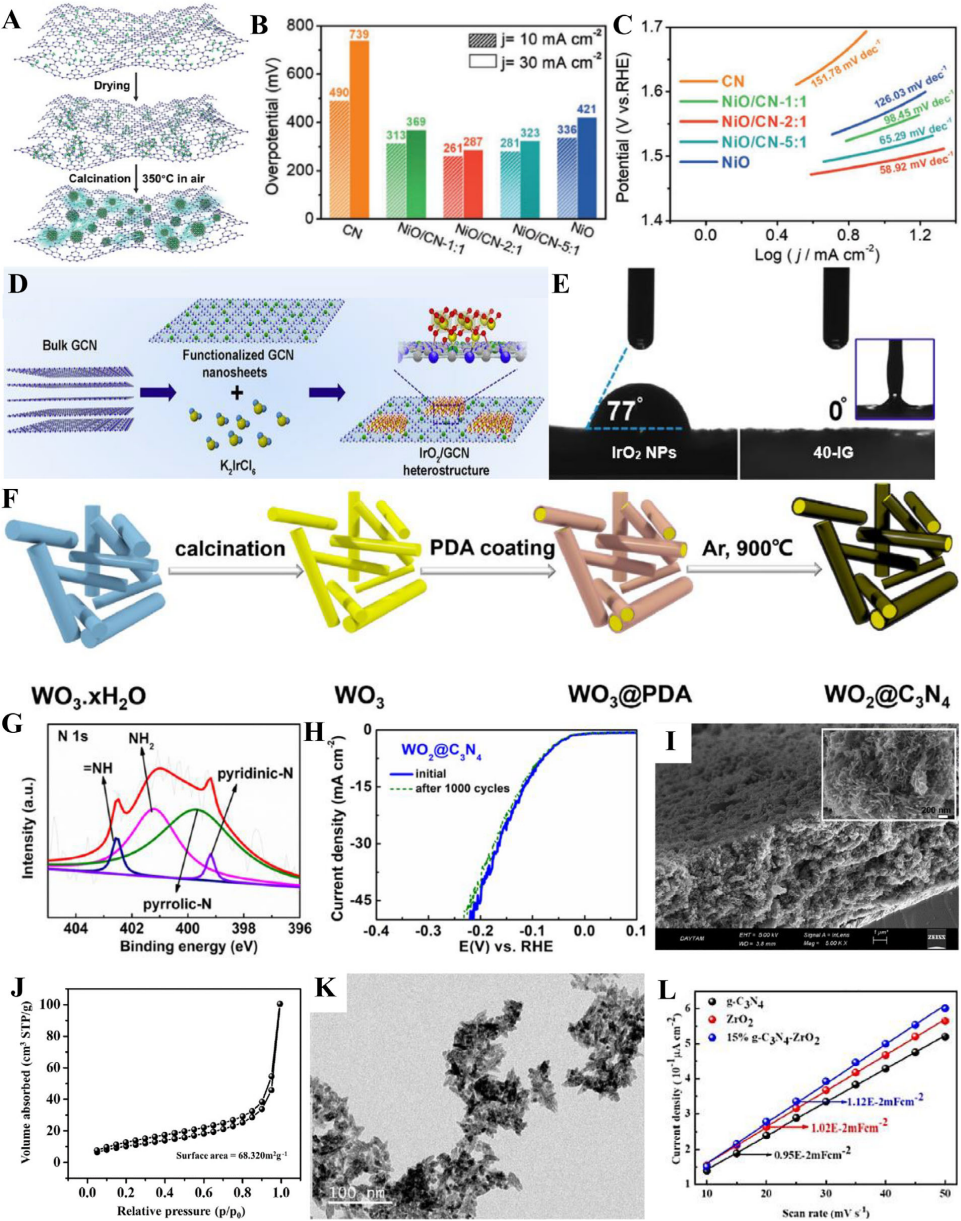
(A) Schematic diagram for the development of NiO/CN (B) Overpotential at 10 and 30 mA cm^−2^ and (C) Tafel plots of pure CN, NiO/CN‐1:1, NiO/CN‐2:1, NiO/CN‐5:1, and pure NiO. Reproduced with permission. [299] Copyright 2019, Wiley‐VCH GmbH. (D) Schematic diagram of the synthesis of IrO_2_/GCN. (E) Contact angle measurement of IrO_2_ and 40‐IG. Reproduced with permission [301]. Copyright 2019, Wiley‐VCH GmbH. (F) Schematic illustration of the formation of WO_2_@C_3_N_4_ nanorods. (G) High resolution XPS spectra of WO_2_@C_3_N_4_ in the N 1s region. (H) Polarization curves of WO_2_@C_3_N_4_ before and after 1000 CV cycles. Reproduced with permission [307]. Copyright 2019, Elsevier Inc. (I) SEM images of 1D MnO_2_@mpg‐C_3_N_4_ coated electrodes at the cross section. Reproduced with permission [308]. Copyright 2019, Elsevier Inc. (J) N_2_ adsorption‐desorption isotherms of Cu_2_O/g‐C_3_N_4_. Reproduced with permission [315]. Copyright 2020, Elsevier Inc. (K) TEM image of 15% g‐C_3_N_4_/ZrO_2_. (l) *C*_dl_ of g‐C_3_N_4_, ZrO_2_, and 15% g‐C_3_N_4_/ZrO_2._ Reproduced with permission [319]. Copyright 2020, Elsevier Inc.

To curb with the electrical conductivity issues, Liao et al. established a facile in situ decomposition method via high temperature calcination (350°C) to synthesize NiO nanocrystals on g‐C_3_N_4_ (NiO/CN), as depicted in Figure 17A. NiO/CN hybrids with different compositions (NiO/CN‐1:1, NiO/CN‐2:1, and NiO/CN‐5:1) were prepared to identify the most optimum OER electrocatalyst using 1.0 m KOH electrolyte. Based on Figure 17B,C, all three NiO/CN hybrids elucidated better results than that of pristine CN and NiO with NiO/CN‐2:1 being the optimum OER electrocatalyst. The promoted OER activity was associated with the intensified electron transmission between NiO and CN [296], which was clearly reflected in the EIS analysis with *R*_ct_ as an indicator. The *R*_ct_ values for CN, NiO, NiO/CN‐1:1, NiO/CN‐2:1, and NiO/CN‐5:1 were 230.6, 49.2, 32.6, 13.4, and 22.9 Ω, respectively. Besides, XPS spectra also confirmed that the NiO and CN were not simply physical mixture as the peak at 852.4 eV in NiO/CN‐2:1 was identified as Ni─N bonds formed at the NiO nanocrystals and CN interface, which was in line with previous literature stating that Ni─N bond is considered metallic and Ni─N─C hybrids are high conductive materials [297, 298], and hence, the reduced R_ct_ in OER as mentioned previously. By virtue of these bonds, the OER stability of NiO/CN‐2:1 can be kept 1.49 V at 10 mA cm^−1^ for 16 h, which is more stable than the commercial RuO_2_ that deviated above the 8 h mark. The upgraded stability was assigned to the synergistic effects between NiO and CN, where the compelling mechanical strength of CN acted as a substrate to suppress NiO nanocrystals from aggregating and falling off from the electrode. Inspired by these results, Liao et al. also studied the adsorption ability of OER intermediates over NiO and NiO/CN models using DFT+U, where surface Ni atoms of NiO is realized as adsorption sites. The Gibbs free energy level of NiO/CN at each intermediate step was distinctly lower than that of NiO suggested that the Ni‐N bonds had effectively minimized the adsorption energy barriers, which ultimately benefitted the OER electrocatalytic process [299]. In short, constructing hybrids with high conductive interfaces is one of the promising solutions to the low electrical conductivities of TMOs, which in general benefits the overall electrocatalytic kinetics.

In another scenario, Chen et al. constructed IrO_2_/GCN heterostructures for acidic (0.5 m H_2_SO_4_) OER via hydrothermal process followed by annealing (Figure 17D). Apart from the impressive interaction between IrO_2_ and GCN similar to that of NiO/CN‐2:1 mentioned in the previous work [299], IrO_2_/GCN heterostructures also ensured a high exposure of ECSA, which was evaluated using *C*_dl_. Pristine GCN, pure IrO_2_, and 40‐IG (IrO_2_/GCN with 40 wt% IrO_2_) recorded *C*_dl_ values of 0.05, 28.5, and 17.0 mF cm^−2^, respectively, which meant that IrO_2_ on the hybrid had accounted for almost 60% (17.0/28.5 = 0.60) of *C*_dl_ and also exposing more active sites since Ir acted as the OER adsorption sites. Despite the negligible *C*_dl_ value of GCN (0.05 mF cm^−2^), the authors discovered the superhydrophilicity properties of GCN surface, which was highly critical for the penetration of electrolyte and gas desorption kinetics [300]. In view of the contact angle measurements in Figure 17E [301], the wettability of IrO_2_ NPs were significantly improved from 77° to 0° upon introducing the GCN nanosheets, a contact angle measurement of 0° was regarded as superhydrophilic or super wetting [302]. Hence, the superhydrophilic 40‐IG had recorded $\eta_{10}$ and Tafel slope of 276 mV and 57 mV dec^−1^, respectively while that of pure IrO_2_ were 297 mV and 82 mV dec^−1^, accordingly [301]. From the point of view of overpotential, IrO_2_ had played a dominant role (60% of the electrocatalyst) in the charge transmission and adsorption sites of 40‐IG, which was the main influence for overpotential; in stark contrast, Tafel slope was commonly affected by the kinetics of an electrocatalyst, and hence, the superhydrophilicity of 40‐IG had contributed towards the O_2_ desorption in the OER, which was proven by the noticeable difference in Tafel slope values.

Principally, pure TM tungsten (W)‐based nanostructures show remarkable electrocatalytic HER activities [303, 304, 305], but their oxide derivatives (WO*_x_*) encounter electrical conductivity deficiencies similar to other TMOs [306]. Herein, a HER electrocatalyst composed of well‐distributed WO_2_ nanorods encapsulated by ultrathin g‐C_3_N_4_ (WO_2_@C_3_N_4_) was fabricated via facile calcination (Figure 17F) and the high graphitization degree of the g‐C_3_N_4_ outer layers were found to simultaneously improve the electrical conductivity as well as protecting the WO_2_ cores from corrosion during HER under acidic environment (0.5 m H_2_SO_4_). The preparation of WO_2_@C_3_N_4_ at high temperature (900°C) in Ar environment had introduced N heteroatoms into the carbon matrix of g‐C_3_N_4_, which was confirmed by the high‐resolution CPS scans in the N 1s region (Figure 17G). Specifically, these electron‐rich N heteroatoms assisted to tune the electronic configuration of the carbon matrix effectively, accomplishing the intrinsic enhanced properties. EIS measurements was performed and the *R*_ct_ values of all samples involved increased in the following order of WO_2_@C_3_N_4_< WO_3_‐Ar < WO_3_< WO_2_< WO_3_‐*x*H_2_O, which revealed that WO_2_@C_3_N_4_ possessed the best electrical conductivity among the rest. The core‐shell structured electrocatalyst also demonstrated phenomenal stability after 1000 CV cycles with negligible current density loss and the polarization curves were observed as almost unaltered (Figure 17H), which was attributed to the physicochemical protection provided by the outer g‐C_3_N_4_ layers [307]. Based on WO_2_@C_3_N_4_ and NiMo/C_3_N_5_ mentioned earlier [268], it can be concluded that the greatest aspect of core‐shell structured hybrids is their incredible stability, which is suitable to be employed in harsh industrial environments.

With the motivation to design open structures for the penetration of electrolyte and transport of ions and electrons, Elmaci et al. assembled 1D MnO_2_ nanowires on mesoporous g‐C_3_N_4_ (MnO_2_@mpg‐C_3_N_4_), which resulted in 3D hybrid nanostructure [308]. The desired objective was achieved upon verification via SEM images under magnification up to 200 nm in scale (Figure 17I), it was inspected that the 1D MnO_2_ nanowires intersected with each other and formed junction points leading to less entanglements in the presence of mpg‐C_3_N_4_. Particularly, the voids and spaces among the nanowires supplied high number of transportation channels and this open structure of MnO_2_@mpg‐C_3_N_4_ favored the quick gas (O_2_ bubbles) release in the OER as aligned with previous literature [309]. Consequently, the TOF of MnO_2_@mpg‐C_3_N_4_ hybrids (0.84 s^−1^ at 480 mV overpotential) was almost three times higher than that of 1D MnO_2_ nanowires (0.32 s^−1^ at 480 mV overpotential) in a neutral environment (PBS at pH 7), which hinted the increment and swift regeneration abilities of the electrochemical active sites. The authors highlighted that the significance of such 3D open structure design rested on its ability to accelerate the mass‐energy transfer process, which minimized the total resistance experienced during the OER [308, 310]. In short, these findings provided in‐depth insights into the use of 3D open structures in electrocatalyst design to control the mass and charge transfer rate of their catalytic performances.

Past experimental studies have narrated that Cu‐based oxides like cuprous oxide (Cu_2_O) own high electrocatalytic activity for OER but the extensive utilization of Cu_2_O as OER electrocatalysts is restricted by its inherent low surface area and instability [311]. In this context, Paul et al. synthesized Cu_2_O/g‐C_3_N_4_ via simple hydrothermal reaction, which presented a 3D crystallography structure confirmed by the XRD studies. As depicted in the N_2_ adsorption‐desorption isotherms (Figure 17J), the BET surface area of Cu_2_O/g‐C_3_N_4_ was 68.32 m^2^ g^−1^, which was significantly higher than that of bare CuO_2_ (21 m^2^ g^−1^) and surpassed other Cu_2_O based nanocomposites namely CuO/Cu_2_O (9.16 m^2^ g^−1^) [312], Cu_2_O‐CuO nanospheres (12 m^2^ g^−1^) [313], and Ag‐Cu_2_O/rGO (14.41 m^2^ g^−1^) [314]. The N_2_ adsorption‐desorption isotherms for Cu_2_O/g‐C_3_N_4_ suggested a type II curve, indicating microporosity of the electrocatalyst. Following the highlighted BET surface areas, the combination of Cu_2_O and g‐C_3_N_4_ was proven to be impressive in HER and yielded an η of 148.7 mV at 12.8 mA cm^−2^ and Tafel slope of 55 mV dec^−1^ in 1.0 m NaOH, which followed the Volmer–Heyrovsky mechanism of HER [315]. The amazing electrochemical activities were ascribed to the large BET surface areas of Cu_2_O/g‐C_3_N_4_, which ensured more adsorption capacity [316]. An in‐depth study on the effect of vacancies towards the BET surface area of 2D C_3_N_4_ was carried out by Zhao and coworkers, they reported that 2D C_3_N_4_ with N vacancies (2D C_3_N_4_‐NV) manifested a BET surface area of 71.3 m^2^ g^−1^, which was 1.7 times that of pure 2D C_3_N_4_ (42.3 m^2^ g^−1^) [317]. Although the fascinating idea of constructing Cu_2_O on N‐vacancy rich g‐C_3_N_4_ has not been realized yet, we envision that the synergistic electronic effects and boosted surface areas will decisively amplify the electrocatalytic behavior of the hybrid in HER and OER.

Another uncommon but sought after specialty of TMO‐based catalysts is their bifunctionality in the mode of catalysis, namely photocatalysis and electrocatalysis of HER or OER [318]. However, photocatalytic activities will not be discussed in this review. A heterostructure composed of ZrO_2_ incorporated g‐C_3_N_4_ (g‐C_3_N_4_/ZrO_2_) was constructed through a simple calcination method, different mass percentages of g‐C_3_N_4_ were investigated. SEM images confirmed that the ZrO_2_ nanoparticles in the 15% g‐C_3_N_4_/ZrO_2_ were dispersed uniformly; while TEM projected the morphology of the electrocatalyst, which existed in spindle shapes (Figure 17K); and the HRTEM captured a clear boundary between ZrO_2_ nanoparticles and g‐C_3_N_4_ nanosheets, hinted the presence of heterojunction formation. The characterization results had pointed out the superior competency of 15% g‐C_3_N_4_/ZrO_2_ with a uniformly scattered adsorption sites (ZrO_2_ nanoparticles), high penetration of electrolyte around the spindle‐shaped morphology, and the efficient charge transfer within the heterostructures. The highest ECSA was also dictated by 15% g‐C_3_N_4_/ ZrO_2_ as shown in the *C*_dl_ plots (Figure 17L). In the HER electrocatalysis under neutral conditions (0.1 m Na_2_SO_4_), 15% g‐C_3_N_4_/ZrO_2_ recorded a lower Tafel slope (550 mV dec^−1^) than that of pristine g‐C_3_N_4_ (1220 mV dec^−1^) and ZrO_2_ (1190 mV dec^−1^). Furthermore, 15% g‐C_3_N_4_/ZrO_2_ was hailed as the most efficient electrocatalyst due to the highest BET surface area of 116.40 m^2^ g^−1^ recorded when compared to the rest: ZrO_2_ (57.71 m^2^ g^−1^), 5% g‐C_3_N_4_/ ZrO_2_ (78.71 m^2^ g^−1^), 10% g‐C_3_N_4_/ZrO_2_ (100.08 m^2^ g^−1^), 20% g‐C_3_N_4_/ZrO_2_ (108.78 m^2^ g^−1^), and 30% g‐C_3_N_4_/ZrO_2_ (80.83 m^2^ g^−1^). This suggested that the specific surface areas of g‐C_3_N_4_/ZrO_2_ enlarged with the addition of g‐C_3_N_4_ until a certain extent where the excess g‐C_3_N_4_ sheets started to overlap with each other [319]. In essence, researchers usually develop highly efficient electrocatalysts by tuning the electrocatalytic layer, however, this work had demonstrated the effectiveness in modulating the composition of the g‐C_3_N_4_ support that proposed a different modification outlook.

**FIGURE 18:**
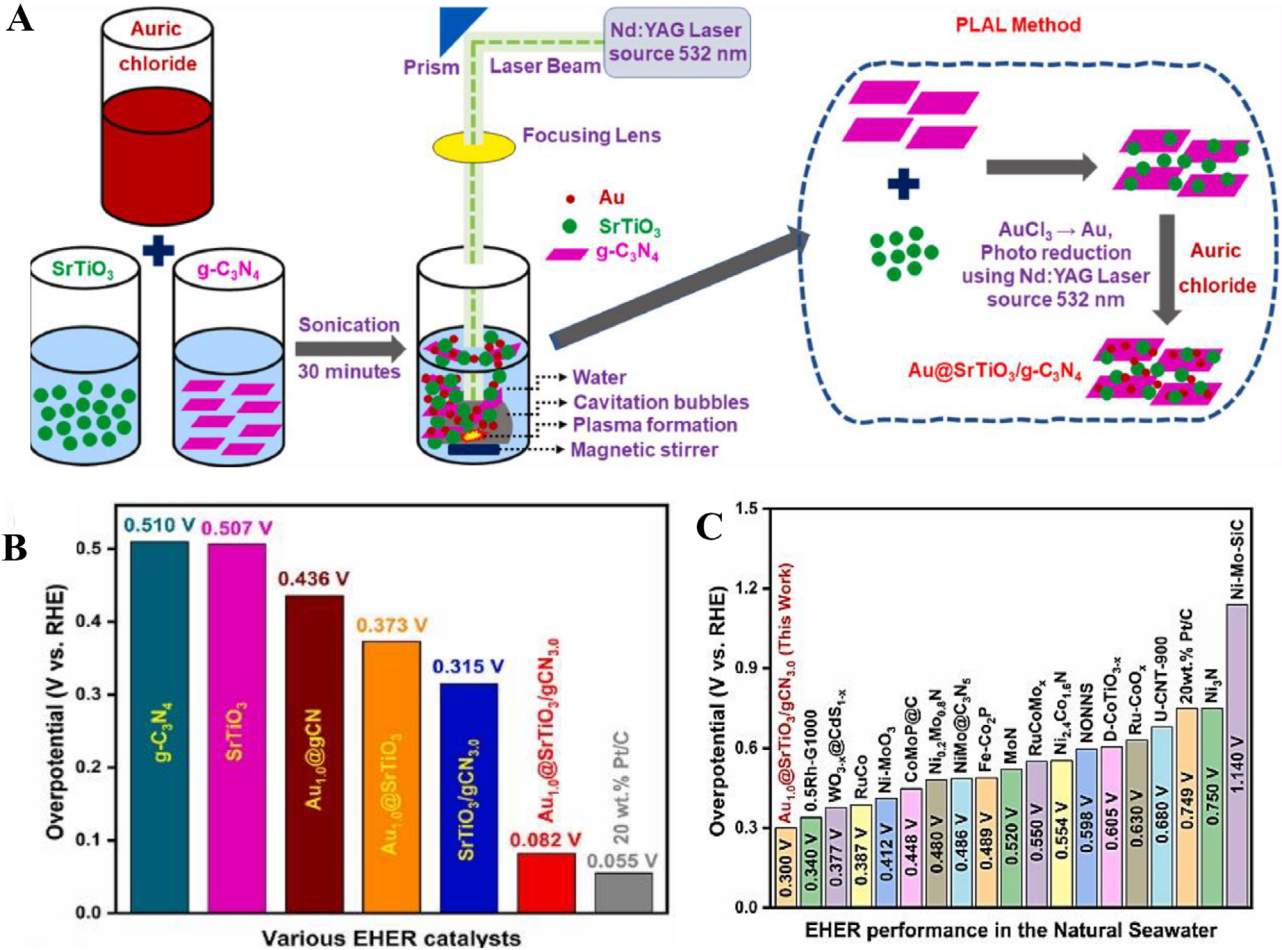
(A) Experimental setup for preparing PLAL with ultrasonic assisted method. (B) Overpotential of HER under acidic condition. (C) HER studies under natural seawater conditions using a cathodic current density of −10 mA cm^−2^ have been conducted in other research. Reproduced with permission [320]. Copyright 2024, Elsevier Inc.

Mohamed et al. developed a ternary nanocomposite, Au@SrTiO_3_/g‐C_3_N_4_, designed for enhanced HER under both acidic and natural seawater conditions. Using an advanced ultrasonic‐assisted pulsed laser ablation technique, they introduced surface oxygen vacancies and Ti^3+^ defects into SrTiO_3_ perovskite, coupling it with g‐C_3_N_4_ and Au nanoparticles to create a synergistic structure (Figure 18A) [320]. The XPS spectra reveal a shift in the Ti2p_1/2_ and Ti2p_3/2_ peaks, indicating a reduction of Ti^4+^ to Ti^3+^. This change confirms the presence of oxygen vacancies on the surface of SrTiO [321]. The optimized composite demonstrated remarkable HER performance with a low overpotential of 0.082 V at −10 mA cm^−2^, a high Tafel slope of 45.36 mV dec^−1^ (Figure 18B). The enhanced electrochemical efficiency of the HER activity was attributed to the synergistic SMSI effect induced by surface oxygen vacancies and Ti^3+^ defects in the PTNCs, which contributed to improved conductivity and surface activity [322]. The inclusion of g‐C_3_N_4_ significantly improved the catalytic activity by increasing ECSA and exposing abundant active sites. However, an excessive amount of g‐C_3_N_4_ reduced performance due to edge sheathing, which hindered active site accessibility and increased recombination centers [323]. HER performance of Au_1.0_@SrTiO_3_/gCN_3.0_ in natural seawater slightly diminishes with increasing testing cycles 0.300 to 0.354 V for the first to 100th cycles at −10 mA cm^−2^ and showed higher activity than the commercial 20 wt% Pt/C catalyst, respectively [109]. The integration of g‐C_3_N_4_ played a crucial role in protecting the Au@SrTiO_3_/g‐C_3_N_4_ electrode from corrosion and poisoning in seawater, enabling stable performance at an overpotential of 0.300 V for 10 h at −10 mA cm^−2^ [324]. Figure 18C compares the HER performance of Au@SrTiO_3_/g‐C_3_N_4_ with previous studies. The study highlights the importance of tuning g‐C_3_N_4_ concentration to balance enhanced activity and stability, demonstrating the potential of these composites for sustainable hydrogen production via interface engineering.

#### 3.3.4 Transition Metal Dichalcogenide (TMDC) Hybrids

**FIGURE 19:**
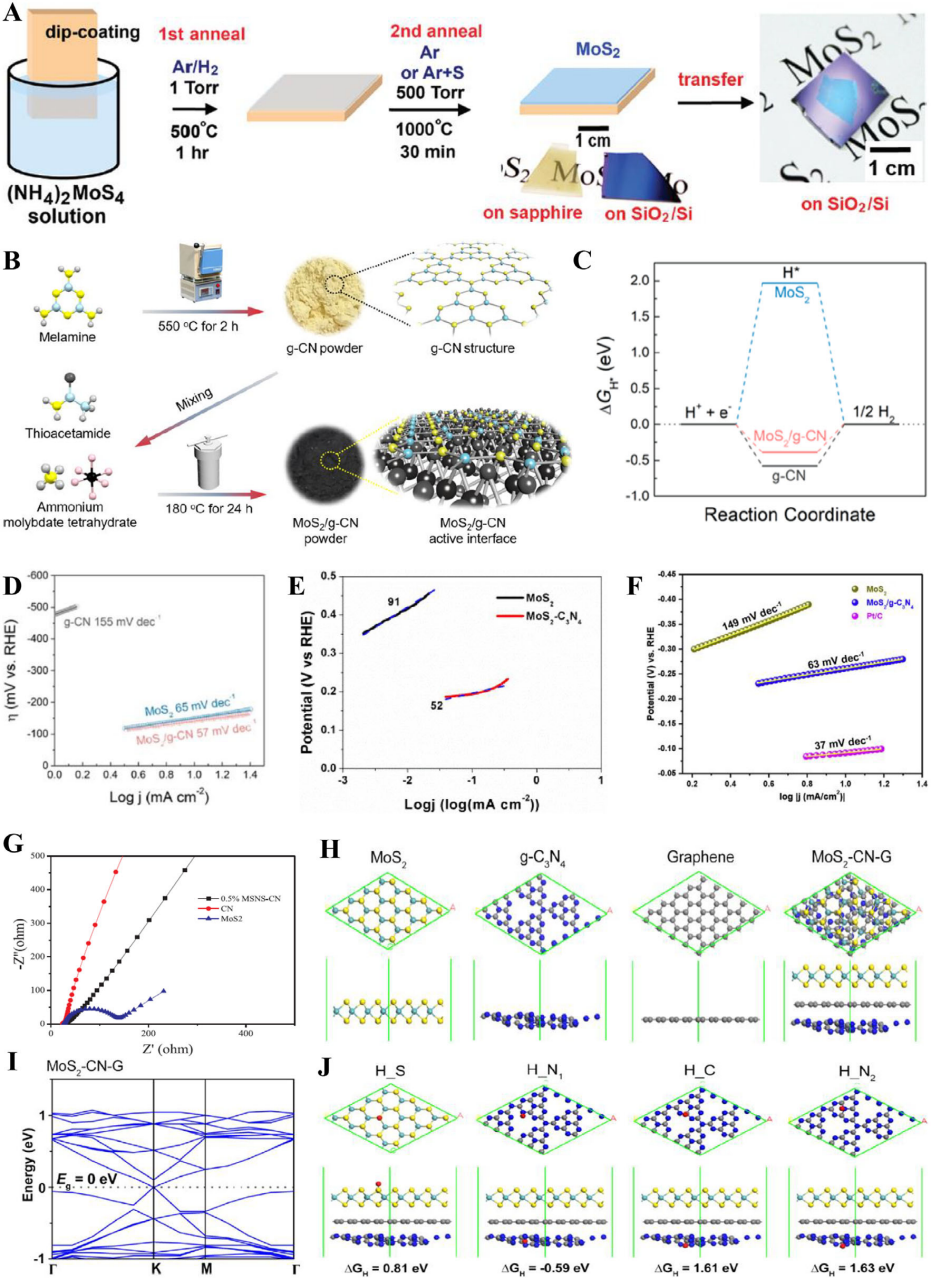
(A) MoS_2_ synthetic pathway via two‐step thermolysis process. Reproduced with permission [330]. Copyright 2012, American Chemical Society. (B) Hydrothermal synthetic pathway and (C) HER Gibbs free energy of MoS_2_/g‐CN (D) Reproduced with permission. [332] Copyright 2020, American Chemical Society. (E) Reproduced with permission [334]. Copyright 2017, Elsevier Inc. (F) Reproduced with permission [333]. Copyright 2017, Elsevier Inc. (F) Tafel plots of different MoS_2_ and g‐C_3_N_4_ hybrid HER electrocatalysts. (g) EIS plot of MSNS‐CN and its pristine counterparts. Reproduced with permission [336]. Copyright 2018, Elsevier Inc. (H) Atomic structures of MoS_2_, g‐C_3_N_4_, G, and MoS_2_─CN─G. The gray, blue, green, and yellow balls represent C, N, Mo, and S atoms, respectively. (I) Hydrogen adsorption sites on H_S (S site of MoS_2_), H_N_1_ (pyridinic N site of g‐C_3_N_4_), H_C (C site of g‐C_3_N_4_), and H_N_2_ (ring N site of g‐C_3_N_4_). (J) Band structure of MoS_2_‐CN/G, where dashed line represents Fermi level. Reproduced with permission [245]. Copyright 2020, American Chemical Society.

Molybdenum disulfide (MoS_2_), a popular representative of transition metal dichalcogenides (TMDCs) is considered a brilliant material comparable to graphene due to its supremacy in transparency, thermal and electrical conductivity, and specific surface area [325, 326, 327]. MoS_2_ can be easily incorporated on graphene or SiO_2_ substrate through chemical vapor deposition (CVD) method [328, 329], as well as via a two‐step thermolysis process (Figure 19A) [330]. An earlier work on DFT had predicted that S‐Mo‐S edges of MoS_2_ were active sites for hydrogen atom adsorption and Mo involves in desirable free energy change in HER [331], which is then proven by Guan et al. with a hydrothermally synthesized (Figure 19B) MoS_2_ hybridized g‐C_3_N_4_ (MoS_2_/g‐CN) electrocatalyst that showed a significantly improved ∆*G*(H_ads_) of −0.39 eV compared to their individual ∆*G*(H_ads_) performance (g‐CN: −0.58 eV; MoS_2_: −0.58 eV), as shown Figure 19C [332]. Apart from remarkably improving g‐C_3_N_4_ as HER electrocatalysts, MoS_2_ hybrids also consistently demonstrated that they proceed the HER via the Volmer–Heyrovsky mechanism with Tafel slopes of 57 mV dec^−1^, 52 mV dec^−1^, 63 mV dec^−1^ (Figure 19D–F) [332, 333, 334]. In one study, the optimal composition of 0D MoS_2_ quantum dots on 2D g‐C_3_N_4_ hybrid (0D/2D MSQD‐CN) was determined. Liu et al. demonstrated that MSQD‐CN with 5 wt% of MS QDs outperformed its counterpart with 2.5 wt% of MS QDs [335], which implied that a higher MoS_2_ content in the hybrid allows better HER performance. In another study, Liu et al. had synthesized a 3D/2D hybrid with flower‐like MoS_2_ nanostructures growing on g‐C_3_N_4_ (MSNS‐CN) through a simple ultrasonic approach. A smaller electrochemical impedance spectroscopy (EIS) arc radius of 0.5 wt% MSNS‐CN compared to pristine g‐C_3_N_4_ (CN) in Figure 19G suggested that the synergistic effects of MoS_2_ on g‐C_3_N_4_ had improved the overall charge migration efficiency of the electrocatalyst, which was mainly due to the manifestation of its impressive electrical conductivity capabilities. Reduction of interfacial charge transfer resistance had brought upon better electrocatalytic activity which is displayed by the improved onset potential shifting from −0.12 V (g‐C_3_N_4_ vs. RHE) to −0.09 V (MSNS‐CN vs. RHE) [336].

Notably, MoS_2_ nanosheets tend to reaggregate during the hybridization process due to the repulsion force exerted by N atoms of g‐C_3_N_4_ heptazine ring, which counters the purpose of fully exposing the active sites. Therefore, FeS_2_ was employed in the ternary architecture of g‐C_3_N_4_/FeS_2_/MoS_2_ hybrid as Fe^3+^ ions were able to prevent agglomeration of MoS_2_ allowing uniform dispersion of MoS_2_ on g‐C_3_N_4_, which formed strong electron transfer channels between g‐C_3_N_4_ and MoS_2_ [337]. In the same context, graphene was employed instead of FeS_2_ in a hybrid consisted of ternary MoS_2_, g‐C_3_N_4_, and graphene (MoS_2_‐CN/G) architectures (Figure 19H) and the 3D porous networks of g‐C_3_N_4_ and graphene were able to prevent the reaggregation of MoS_2_ nanosheets. The synergistic effects of this electrocatalyst were also studied mathematically by its band structures. The band gaps (*E*_g_) of MoS_2_, g‐C_3_N_4_, graphene, and MoS_2_‐CN‐G (Figure 19I) were 1.80, 1.85, 0, and 0 eV accordingly. It can be obviously observed that the incorporation of graphene had a great impact on the band structures, which was able to fine tune the electronic conductivity of the MoS_2_─CN─G electrocatalyst, yielding an excellent HER performance ($\eta_{10}$ of 140 mV and Tafel slope of 79 mV dec^−1^, indicating Volmer–Heyrovsky mechanism similar to that of binary MoS_2_ hybrids). On top of that, Yan et al. had also investigated four possible H atom adsorption sites (Figure 19J) using DFT studies on the MoS_2_─CN/G electrocatalyst, H_N_1_ was considered the most optimal H atom adsorption site as its ∆*G*(H_ads_) = −0.59 eV (closest to ∆*G*(H_ads_) = 0 eV), followed by H_S site with ∆*G*(H_ads_) = 0.81 eV [245]. DFT results obtained signifies that MoS_2_ and g‐C_3_N_4_ layers contributed greatly in the electrocatalytic activity, while graphene was mostly responsible for electrical conductivity.

**FIGURE 20:**
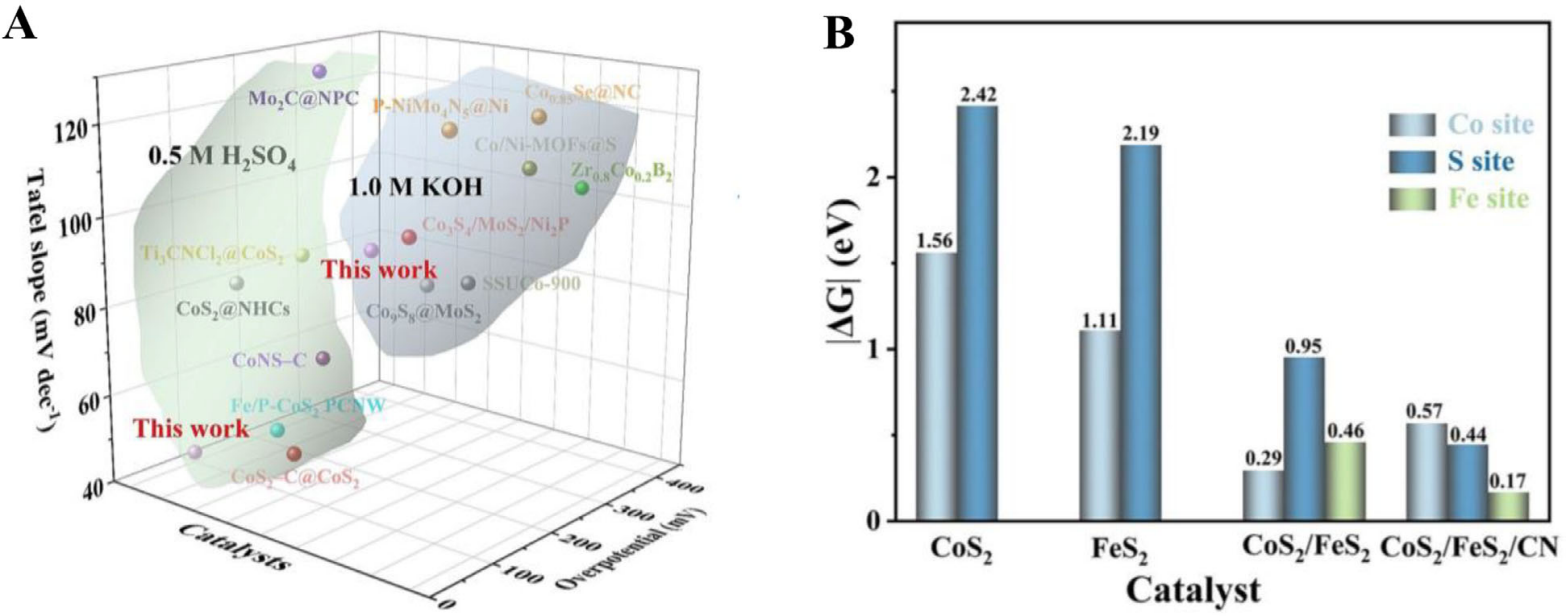
(A) Comparison of the Tafel slopes and overpotentials in the work and other reported result the literature. (B) Comparison of the H adsorption‐free energy at different active sites. Reproduced with permission [338]. Copyright 2024, Elsevier Inc.

Other than MoS_2_, Dong et al. explored the application of bimetallic Co─Fe sulfide ultrathin nanosheets supported on g‐C_3_N_4_ in enhancing electrocatalytic HER performance. They synthesized CoS_2_/FeS_2_/CN, using a combination of CoS_2_, FeS_2_, and g‐C_3_N_4_ through dual doping strategy. [338] The CoS_2_/FeS_2_/CN catalyst exhibited remarkable HER performance, requiring overpotentials of only 76.5, 145.5, and 218.5 mV in acidic media to reach 10, 50, and 100 mA cm^−2^, respectively. The Tafel slope of CoS_2_/FeS_2_/CN was 44.9 mV dec^−1^, higher than that of Pt/C but lower than other synthesized catalyst, signifying significantly faster. In alkaline media, the CoS_2_/FeS_2_/CN catalyst achieves current densities of 10 and 50 mA cm^−2^ with overpotentials of 175.6 and 288.3 mV, respectively (Figure 20A). It demonstrates the lowest Tafel slope (93.5 mV dec^−1^) among the literature, except for Pt/C. Furthermore, CoS_2_/FeS_2_/CN exhibit an excellent HER stability in both condition for 24 h at 10 mA cm^−2^ with minimal degradation after 5000 CV cycles. DFT calculations revealed that the CoS_2_/FeS_2_/CN heterostructure exhibited a Δ*G*H* of 0.17 eV at Fe sites, indicating near‐ideal hydrogen adsorption for HER (Figure 20B). This electronic modulation at the heterostructure interface, combining the advantages of CoS_2_, FeS_2_, and g‐C_3_N_4_, was the key to its exceptional catalytic activity. This study provides a promising pathway for designing efficient, low‐cost HER electrocatalysts with broad applicability across various pH conditions.

#### 3.3.5 Other Metal Hybrids

**FIGURE 21:**
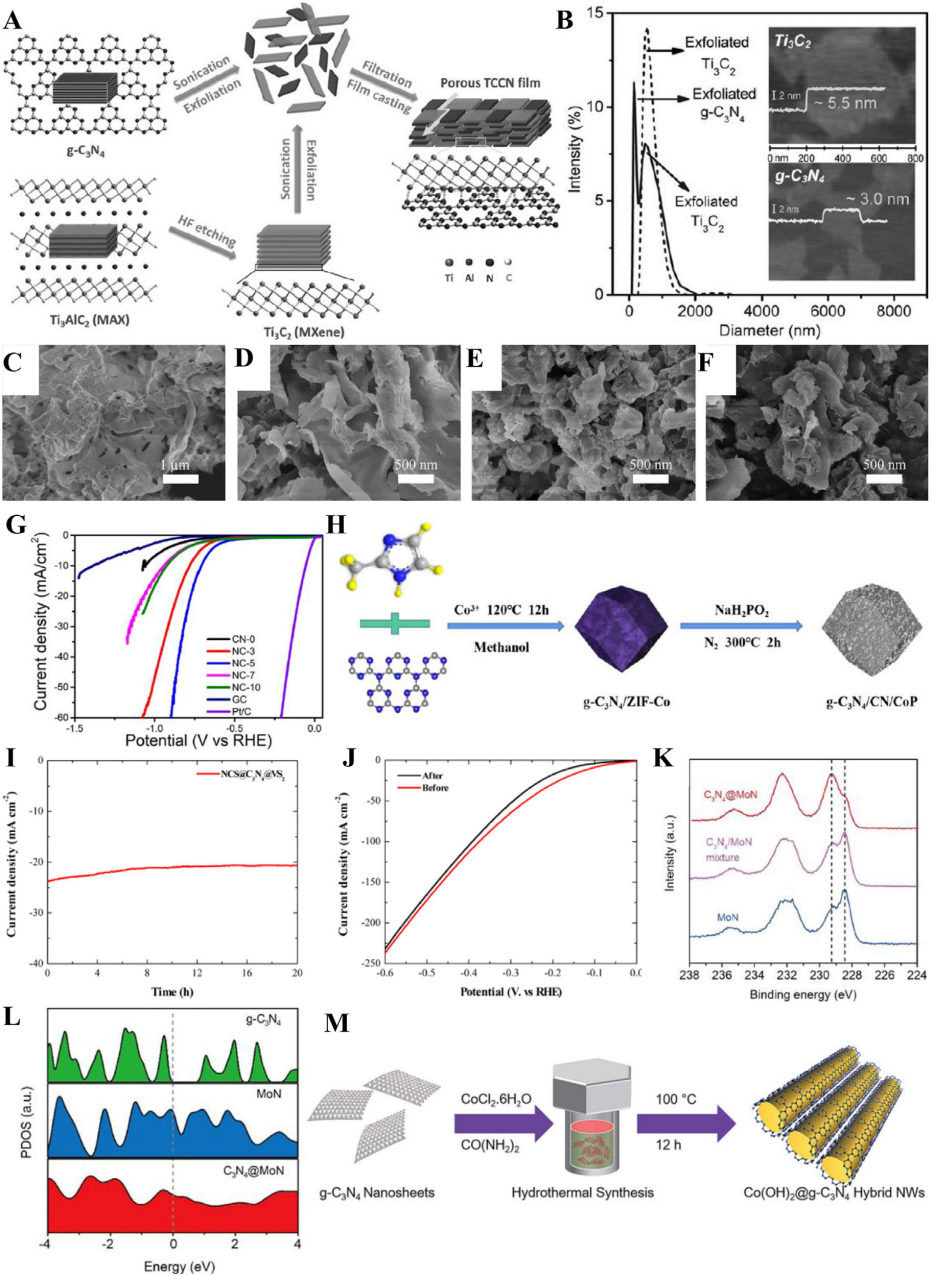
(A) Schematic illustration of the preparation of porous TCCN film. (B) AFM images of exfoliated Ti_3_C_2_ and g‐C_3_N_4_. Reproduced with permission [342]. Copyright 2016, Wiley‐VCH GmbH. (C–F) SEM images for CN‐0, NC‐3, NC‐5, and NC‐7. Reproduced with permission [345]. Copyright 2018, American Chemical Society. (G) Schematic illustration of the construction of g‐C_3_N_4_/CN/CoP hybrid. Reproduced with permission [350]. Copyright 2022, Elsevier Inc. (H) Tafel slope (I) Chronopotentiometric measurements and (J) polarization curves before and after 20 h of NiCo_2_S_4_@C_3_N_4_@VS_2_. Reproduced with permission [356]. Copyright 2020, Elsevier Inc. (K) Mo 3d XPS spectra of C_3_N_4_@MoN, C_3_N_4_/MoN mixture, and MoN. (L) Theoretical density of states of g‐C_3_N_4_, MoN, and C_3_N_4_@MoN. Reproduced with permission [359]. Copyright 2018, Elsevier Inc. (M) Schematic diagram of the fabrication of Co(OH)_2_@g‐C_3_N_4_ hybrid NWs. Reproduced with permission. Copyright 2016, Royal Society of Chemistry.

Since the discovery of HER activity on dimolybdenum carbide (Mo_2_C) by Vrubel et al. in 2012 [339], the research on transition metal carbide (TMC) electrocatalysts has experienced a rapid hike. A detailed review specifically on HER using various heterogeneous TMC electrocatalysts was covered [340], focusing mainly on the intrinsic electrocatalysis as well as the structural and electronic modulation. It is noteworthy that TMCs share similar catalytic and electronic properties with the noble Pt metal [341], and thus, the replacement of Pt with TMCs can economically reduce the electrocatalyst's production cost. Additionally, 2D layered TMCs is also labeled as MXene, which underlines the similarity to graphene. In this context, Ma and co‐workers fabricated a hybrid film comprised of overlapped Ti_3_C_2_ and g‐C_3_N_4_ nanosheets (TCCN) via homogenous assembly using Ti_3_AlC_2_ MAX phase as a precursor (Figure 21A) [342]. The nanosheet morphology of TCCN was verified by SEM and atomic force microscopy (AFM) images, revealing the thickness of 5.5 and 3.0 nm for Ti_3_C_2_ and g‐C_3_N_4_, respectively (Figure 21B). TCCN nanosheets also possessed a high surface area of 205 m^2^ g^−1^ by virtue of their hierarchical pores, which afforded a high *C*_dl_ (29.7 mF cm^−2^) and approximately three times higher than that of TCCN in powdered form (after subjected to ball‐milling, *C*_dl_ = 10.3 mF cm^−2^). As a self‐supported and binder‐free electrocatalyst, this configuration was also more effective in enlarging the ECSA in contrary to other supported electrocatalysts [343]. On that note, TCCN film recorded magnificent OER results in 0.1 m KOH aqueous media with an onset potential at 10.0 mA cm^−2^ (*E_j_* = 10) of 1.65 V, which was lower than those of state‐of‐the‐art electrocatalysts IrO_2_/C and RuO_2_ at 1.70 V and 1.73 V [344], respectively under similar OER conditions. In addition, another apparent advantage of self‐standing electrocatalysts is their robust mechanical stability, these configurations will prevent the exfoliation of catalytic materials from the catalyst support. The aforementioned statement was evidently proven in this work via chronoamperometric test at 10 mA cm^−2^ of TCCN and benchmark IrO_2_/C electrocatalysts, the chronoamperometric revealed that TCCN only manifested a slight anodic current attenuation of 4.3% after 10 h of testing while that of IrO_2_/C suffered an attenuation up to 29.7% under similar conditions. In conclusion, TCCN has shown potent signs of highly efficient self‐supported electrocatalyst, which is highly sought after in practical applications as self‐standing electrocatalysts are generally more stable and the cost for replacing the catalyst support is not necessary anymore.

By virtue of the magnificent physical and chemical properties of P‐block elements, Cao et al. modified g‐C_3_N_4_ with different nickel boride (Ni_2_B) compositions (0, 0.03, 0.05, 0.07, and 0.1 wt%) denoted as CN‐0, NC‐3, NC‐5, NC‐7, and NC‐10, accordingly via thermal treatment at 500°C for 2 h [345]. The impact of Ni_2_B composition variance on the surface morphology of the hybrid samples were clearly unveiled through the SEM images (Figure 21C–F). Pristine g‐C_3_N_4_ presented a smooth and flat g‐C_3_N_4_ multilayers, which agreed well with that of previous literature (Figure 21C) [346, 347]. The addition of Ni_2_B aided the exfoliation and separation of the g‐C_3_N_4_ sheets, which introduced higher degrees of porosity with increasing Ni_2_B composition as witnessed in Figure 21F. The most optimum hybrid was determined through HER activity in alkaline solution (1.0 m KOH), NC‐5 recorded the lowest $\eta_{10}$ and Tafel slope (707 mV and 221 mV dec^−1^) among other prepared samples CN‐0 (1056 mV and 372 mV dec^−1^), NC‐3 (900 mV and 348 mV dec^−1^), NC‐7 (915 mV and 350 mV dec^−1^), and NC‐10 (923 mV and 339 mV dec^−1^). The aforementioned electrochemical results concluded that 0.05 wt% of Ni_2_B was the optimal composition for this electrocatalyst, indicated that NC‐3 did not achieve the desired porosity while that of NC‐7 and NC‐10 suffered excessive porosity, which essentially reduced some of the adsorption sites. The enlargement of specific area by incorporating Ni_2_B was also proven when NC‐5 exhibited BET surface area of 154 m^2^ g^−1^ while CN‐0 only recorded 42 m^2^ g^−1^. Other than the contribution by its morphology, the presence of Ni atoms also enriched the B atoms on the surface, which provided additional electrons to the active site. In this work, the synergistic effects between Ni_2_B and g‐C_3_N_4_ were detected by virtue of two phenomena: (1) the electrons transfer between Ni and B, followed by Ni_2_B and g‐C_3_N_4_ had significantly facilitated the charge transfer process, which lowered the required overpotential for HER;(2) Ni_2_B acted as a spacer for g‐C_3_N_4_ that was responsible for preventing g‐C_3_N_4_ layers restacking, similar to that of RGO spacers for MXenes discussed previously [243]. However, despite being the best sample among its peers, NC‐5's performance had trailed behind the benchmark Pt/C electrocatalyst by a huge margin visibly on the LSV polarization curves, as well as the $\eta_{10}$ and Tafel slope of Pt/C at 56 mV and 36 mV dec^−1^, respectively. In short, this work lays the foundation for hybrids involving g‐C_3_N_4_ with that of metal borides in electrocatalytic HER, which inspires researches to explore deeper into different applications, for instance, electrocatalytic OER and OWS using different metal borides.

Previously, transition metal phosphides (TMPs) were reported to show satisfying HER results as a cocatalyst [348]. With respect to g‐C_3_N_4_, a highly efficient HER CoP/g‐C_3_N_4_ photocatalyst was synthesized for the first time, which produced a H_2_ generation rate up to 130 times higher than that of pristine g‐C_3_N_4_ [349]_._ Inspired by the previous research, Qamar and Liu et al. formulated cobalt phosphide (CoP) based HER electrocatalysts with an emphasis on g‐C_3_N_4_ serving different purposes [350, 351]. Qamar used g‐C_3_N_4_ as a catalyst support for the uniform dispersal of CoP NPs; while Liu et al. constructed g‐C_3_N_4_(*x*)/CN(*y*)/CoP(*z*) hybrid by utilizing g‐C_3_N_4_ as a cocatalyst for CoP via in situ high temperature phosphorization process (Figure 21G), where *x*, *y*, and *z* represents the weight composition of g‐C_3_N_4_, the phosphating temperature, and the mass ratio of NaH_2_PO_2_ to g‐C_3_N_4_, respectively. Inevitably, both researches unanimously proved that CoP/g‐C_3_N_4_ and g‐C_3_N_4_(90)/CN300/CoP3 accomplished better HER activities in acidic condition (0.5 m H_2_SO_4_) compared to pristine CoP. In Qamar's work, η achieved by CoP and CoP/g‐C_3_N_4_ were 280 and 157 mV, respectively at 8.5 mA cm^−2^; while CoP and g‐C_3_N_4_(90)/CN300/CoP3 obtained $\eta_{10}$ of 480 and 221 mV, respectively in Liu et al.’s work. Also, these two HER electrocatalysts operated under the same Volmer–Heyrovsky mechanism with Tafel slope values of 66 (CoP/g‐C_3_N_4_) and 115.1 mV dec^−1^ (g‐C_3_N_4_(90)/CN300/CoP3), accordingly. With regard to electrochemical stability, CoP/g‐C_3_N_4_ surpassed g‐C_3_N_4_(90)/CN300/CoP3 (7 h) up to 12 h under similar aqueous condition, indicated that CoP NPs dispersed on g‐C_3_N_4_ was more durable than the hybridization of CoP and g‐C_3_N_4_ [350, 351]. Owing to the interfacial charge transfer between the uniformly dispersed CoP NPs and g‐C_3_N_4_, a unique metal‐support interaction was induced, which led to the improvement of stability as well as enhancing the electrocatalytic activity as a whole [351, 352]. In essence, both surface‐dispersion and cocatalyst‐hybridization are functional synthetic methods in developing highly efficient electrocatalysts, however, the comparison studies of different catalyst design methods are currently lacking in HER, OER and OWS. The reason being researches that compare the performance of different synthetic methods under similar conditions can create the opportunities to unravel the underlying factors that influence the electrocatalytic HER, OER, and OWS process.

Transition metal sulfides (TMSs) especially nickel cobalt sulfides (NiCo_2_S_4_) are anticipated to display remarkable electrochemical HER activities as a result of the optimized electronic structures by the addition of the second metal atom [353]. For instance, Jiang et al. constructed NiCo_2_S_4_ double‐shell ball‐in‐ball hollow spheres (NiCo_2_S_4_ BHSs) for alkaline HER, which delivered exceptional performance with a small $\eta_{10}$ of 89.7 mV in 0.1 m KOH [354]. Nevertheless, the NiCo_2_S_4_ hollow nanostructures are restricted for industrial applications ascribed to their structural degradation during extensive redox process which led to poor cycling stability. One of the promising resolutions in addressing the aforementioned subject was realized by Sekar et al. In this work, ultrathin VS_2_ nanosheets were vertically aligned on the g‐C_3_N_4_ coated NiCo_2_S_4_ through multiple hydrothermal and calcination reactions forming a hollow NiCo_2_S_4_@C_3_N_4_@VS_2_ hybrid nanostructure, which was bifunctional in asymmetric supercapacitor and alkaline HER applications. The incorporation of another TMS known as vanadium sulfide (VS_2_) into the hybrid was vital to modulate the capacitance and cycle life by virtue of their huge surface area and decreased lengths of ion diffusion path [355]. Ergo, NiCo_2_S_4_@C_3_N_4_@VS_2_ shows a relative low Tafel slope with 71.8 mV dec^−1^ and its chronopotentiometry measurements of HER at 20 mA cm^−2^ experienced a negligible degradation of current density after 20 h in 1.0 m KOH aqueous media (Figure 21H,I), which expressed signs of great HER activity and long‐term stability. Next, the polarization curves before and after 20 h of chronopotentiometry test also specified a slight shift in overpotential (Figure 21J), which further confirmed the improved stability of the electrocatalyst. Without a doubt, NiCo_2_S_4_@C_3_N_4_@VS_2_’s electrochemical properties were superior than that of pure NiCo_2_S_4_ and NiCo_2_S_4_@C_3_N_4_, which benefited from the synergistic effect of NiCo_2_S_4_, C_3_N_4_, and VS_2_ in the hybrid structures. NiCo_2_S_4_ provided good electrical conductivity accredited to the dual‐metallic atom configuration, which enriched the catalytic active sites; N‐rich g‐C_3_N_4_ created more active sites and served as a wonderful support for NiCo_2_S_4_ and the vertically aligned VS_2_; lastly, the large surface area of VS_2_ enabled rapid charge transfer within the electrocatalyst along with regulating the stability of the hybrid [356]. In short, the as‐synthesized electrocatalyst is regarded as a multi‐component hybrid involving three different species (NiCo_2_S_4_, C_3_N_4_, and VS_2_) playing different roles, which simultaneously settles the instability of NiCo_2_S_4_ as well as improving the overall electrocatalytic HER performance.

Normally, transition metal nitride (TMN) based materials are less dominant in the field of electrocatalysis as opposed to TMCs due to the high reputation of MXene materials. With the intention to contribute towards this research gap, Xiao and coworkers proposed an efficient strategy to synthesize 2D molybdenum nitride (MoN) electrocatalyst with large yield, they found that 2D MoN possessed excellent corrosion resistance and electrical conductivity, which was highly promising in competing with the likes of TMC‐based electrocatalysts [357]. Adopting similar salt‐templated method, Jin et al. first prepared 2D MoN, followed by the construction of a heterostructure consisted of 2D MoN and 2D g‐C_3_N_4_ (C_3_N_4_@MoN). The molecular level interaction of MoN and g‐C_3_N_4_ was studied by high‐resolution Mo 3d XPS spectra (Figure 21K), it was detected that C_3_N_4_/MoN mixture and pristine MoN shared matching spectra patterns, implied that there was no chemical interaction occurred in the mixture. Conversely, C_3_N_4_@MoN generated a spectrum where the concentration of Mo^3+^ (approximately 228.5 eV) was lower than Mo^4+^ (approximately 229.2 eV), which meant that the valence state of Mo atoms in the hybrid was higher than that of C_3_N_4_/MoN mixture and pristine MoN. The higher valence state Mo atoms induced stronger adsorption energy for intermediates like OH_ads_ and hence expedited the water dissociation process [357, 358]. On the other hand, the atomic level charge transfer between g‐C_3_N_4_ and MoN was unveiled by DFT calculations. As depicted in Figure 21L, pristine g‐C_3_N_4_ and MoN manifested semi‐conductive and conductive characteristics, respectively while C_3_N_4_@MoN contained more states near the Fermi level, which was attributed to the electron transfer from MoN to g‐C_3_N_4_ at the interlayer of the hybrid. The electron redistribution during the hybridization process confirmed the strong interaction between both species and more importantly, modifying the electronic structure and adsorption capacities. Indeed, C_3_N_4_@MoN topped both C_3_N_4_/MoN mixture and pristine MoN in alkaline HER (1.0 m KOH) with $\eta_{10}$ and Tafel slope of 110 mV and 57.8 mV dec^−1^, respectively up to 10 h of stability test. Incidentally, the authors discovered that C_3_N_4_@MoN also functioned under dual active sites for the intermediates H_ads_ and OH_ads_ and concluded that maintaining the balance between both adsorbed intermediates is the key towards highly efficient water dissociation and H_2_ production [359].

As mentioned in earlier sections, Co‐based materials were able to modulate the intrinsic activities of an electrocatalyst [216], which allows direct creation of more active sites for OER. Thus, combining Co with g‐C_3_N_4_ and introducing a hybrid structure is an ideal way to make use of their respective active sites. Along with this, the consolidation of aqueous ions also helps to increase the electrode wettability and ionic conductivity, which is an important element highlighted by Chen and coworkers [301]. To exploit the merits of aforementioned elements, Tahir et al. fabricated a hybrid containing cobalt hydroxide nanowires coated by g‐C_3_N_4_ nanosheets denoted by Co(OH)_2_@g‐C_3_N_4_ via facile hydrothermal process (Figure 21M). Pristine g‐C_3_N_4_, pure Co(OH)_2_ NWs, and Co(OH)_2_@g‐C_3_N_4_ with different g‐C_3_N_4_ compositions (2, 5, and 7 wt%) were tested in 1.0 m KOH electrolyte, the onset potential was chosen as a study parameter, which were 1.65, 1.65, 1.48, 1.47, and 1.49 V, respectively. Hence, the increment of g‐C_3_N_4_ content delivered better performance, but an excessive amount of g‐C_3_N_4_ lowered the OER activity as a consequence of thicker outer sheets that restricted electrolyte penetration and slower ionic transport. Strong synergistic coupling between Co(OH)_2_ NWs and g‐C_3_N_4_ permitted the faster electron transfer to and from the electrochemical active sites, the engineered structure and improved wettability of the hybrid enabled easy access of the electrolyte, which led to a more superior OER outcome when competing with benchmark IrO_2_ and RuO_2_ electrocatalysts. Co(OH)_2_@g‐C_3_N_4_‐5 recorded $\eta_{10}$ and Tafel slope of 1.55 V and 59 mV dec^−1^, respectively while IrO_2_ recorded 1.65 V and 65 mV dec^−1^, accordingly, and RuO_2_ obtained 1.58 V and 90 mV dec^1^, respectively. Lastly, Co(OH)_2_@g‐C_3_N_4_‐5 displayed an impressive stability by retaining a high efficiency of 98.9% after 10 h of testing [360]. In summary, the strong synergistic effect between different components of a hybrid is highly significant as it gives rise to desired qualities that facilitate the electrocatalytic reactions. Table 2 summarizes performance of C_x_N_y_ in electrocatalytic HER and OER.

**TABLE 2:** Electrocatalytic performance of various C*_x_* N*_y_* based catalysts in HER and OER.

Catalyst	Modification	Overpotential [mV]	Tafel slope [mV dec^−1^]	Current density [mA cm^−2^]	Catalytic Stability	Medium	Ref.
**Electrocatalytic HER**							
Co_1_/PCN	Co atoms atomically dispersed in the framework of phosphorized carbon nitride, forming single‐atom active sites	89	52	10	24 h	1.0 m KOH	[266]
Ru@C_2_N	Ru nanoparticles dispersed on a nitrogenated holey 2D carbon framework	22 28 34.8	30	10 15 20	10,000 cycles	0.5 m H_2_SO_4_	[255]
		17 27 35.5	38	10 15 20	10,000 cycles	1.0 m KOH	
Co‐CNG	Low‐coordination single‐atom Co on g‐C_3_N_4_	47	44	10	500 h	1.0 m KOH	[261]
CNQDs@G	Atom‐think C_3_N_4_ quantum dots supported on graphene	110	53	10	10 h	0.5 m H_2_SO_4_	[232]
g‐CN@G MMs	Mesoporous g‐C_3_N_4_ formed in‐situ on mesoporous graphene to yield mesh‐on‐mesh structure	219	53	10	∼14 h	0.5 m H_2_SO_4_	[231]
CN/C‐Dots_100_	One step polymerization of supramolecular complexes (melamine, cyanuric acid, and β‐cyclodextrin	415	92	10	−	0.5 m H_2_SO_4_	[224]
		340	110	10	−	1.0 m KOH	
FeSe_2_/g‐C_3_N_4_	Construction of FeSe_2_ on g‐C_3_N_4_	1630	70	10	1000 cycles	0.5 m H_2_SO_4_	[361]
MoS_2_‐CN/G	3D ternary hybrid constructure from graphene, MoS_2_ and g‐C_3_N_4_	332	79	10	2000 cycles	0.5 m H_2_SO_4_	[245]
Ru/g‐C_3_N_4_‐C‐TiO_2_	Ru single atoms dispersed on oxygen vacancy‐rich g‐C_3_N_4_‐C‐TiO_2_ hybrid	112	83	10	10 h	0.5 m H_2_SO_4_	[362]
		107	65	10	10 h	1.0 m KOH	
C_3_N_4_‐MoS_2_	g‐C_3_N_4_ covalent cross‐linked with MoS_2_	278	88	5	3	0.5 m H_2_SO_4_	[363]
C_3_N_4_‐NRGO	g‐C_3_N_4_ covalent cross‐linked with nitrogenated reduced graphene oxide	506	147	5	3	0.5 m H_2_SO_4_	
Porous Pd‐CN*_x_*	Reduction of PdCl_2_ in the presence of g‐C_3_N_4_ nanosheets with NaBH_4_ and ultrasound treatment	55	35	10	10,000 cycles	0.5 m H_2_SO_4_	[251]
B‐CN/PC	P‐block elements heteroatoms doped (B, P, S) g‐C_3_N_4_ supported on macroporous carbon	405	93	10	3000 cycles	0.5 m H_2_SO_4_	[180]
P‐CN/PC		343	100	10			
S‐CN/PC		186	84	10			
PCN@N‐graphene‐750	2D porous g‐C_3_N_4_ integrated into N‐doped graphene sheets.	80	49.1	10	5000 cycles	0.5 m H_2_SO_4_	[237]
MoS_2_/g‐CN	Incorporation of g‐C_3_N_4_ onto the bulk MoS_2_ support	141	57	10	12 h	0.5 m H_2_SO_4_	[332]
CoO*_x_*/mC@ MoS_2_@g‐C_3_N_4_	Hybridization of Co‐nanomaterials, MoS_2_ and N‐rich g‐C_3_N_4_	70	66	10	4 h	0.5 m H_2_SO_4_	[364]
Ni_2_B/g‐C_3_N_4_	Ni_2_B nanomaterials modified g‐C_3_N_4_	707	221	10	1000 cycles	1.0 m KOH	[345]
Ni/C_3_N_4_	Electrodeposition of Ni on g‐C_3_N_4_	356	128	100	12 h	1.0 m NaOH	[365]
		222	128	10	−	1.0 m NaOH	
g‐C_3_N_4_ nanoribbon‐G	Assembly of 1D in‐situ formed g‐C_3_N_4_ nanoribbons on 2D graphene sheets to form 3D interconnected networks	207	54	10	−	0.5 m H_2_SO_4_	[230]
MSNS‐CN	3D flower‐like MoS_2_ nanoparticles attached to g‐C_3_N_4_ nanosheets	200	96	10	−	0.5 m H_2_SO_4_	[336]
Ir‐g‐CN Ru‐g‐CN	Single atom Ir and Ru anchored on mesoporous g‐C_3_N_4_	41.9 54.5	47.6 54.0	10	3 h	0.5 m H_2_SO_4_	[198]
		60.2 75.5	49.3 63.5	10	3 h	1.0 m KOH	
NCS@C_3_N_4_@VS_2_	Ultrathin VS_2_ nanosheets vertically aligned on C_3_N_4_ coated NiCo_2_S_4_ hollow nanospheres	110	71.8	10	20 h	1.0 m KOH	[356]
WO_2_@C_3_N_4_	WO_2_ nanorods encapsulated by ultrathin C_3_N_4_	98	94.4	10	1000 cycles	0.5 m H_2_SO_4_	[307]
0.31Cu‐C_3_N_4_	Cu ions doped g‐C_3_N_4_	390	76	10	43 h	0.5 m H_2_SO_4_	[197]
Mo‐S‐CN	Mo and S co‐doping on g‐C_3_N_4_	630	110	10	5 h	1.0 m KOH	[204]
g‐C_3_N_4_@P‐pGr	P‐doped nanoporous graphene integrated with 2D g‐C_3_N_4_	340	90	10	8.3 h	0.5 m H_2_SO_4_	[366]
g‐C_3_N_4_/FeS_2_/MoS_2_	Uniform dispersion of MoS_2_ on g‐C_3_N_4_ with the aid of FeS_2_	193	87.7	10	3000 cycles	0.5 m H_2_SO_4_	[337]
PtRu@C_2_N	PtRu allow nanoparticles embedded on 2D C_2_N matrix	52	31	10	10,000 cycles	0.5 m H_2_SO_4_	[367]
		59	63	10	10,000 cycles	0.1 m KOH	
NiMo@C_3_N_5_	Core‐shell heterostructures composed of 1D NiMo cores and 2D C_3_N_5_ shells	81	68.3	10	10 h	1.0 m KOH	[268]
		486	−	10	10 h	Natural seawater (pH =7.4)	
Ru‐DCN	CO_2_‐assisted anti‐solvent growth of defect‐rich g‐C_3_N_4_ and loading of Ru nanoparticles	51	59.5	10	24 h	0.5 m H_2_SO_4_	[368]
Ru‐C_3_N_4_/rGO	Ru ions complexed C_3_N_4_ integrated with reduced graphene oxide	80	55	10	9 h	0.5 m H_2_SO_4_	[369]
Cu_2_O/g‐C_3_N_4_	Cu_2_O encapsulated with g‐C_3_N_4_	148.7	55	12.8	−	1.0 m NaOH	[315]
30P‐rGO‐g‐C_3_N_4_	Red phosphorus embedded in rGO‐g‐C_3_N_4_ composite	575	122.5	10	500 cycles	0.5 m H_2_SO_4_	[370]
MoS_2_/g‐C_3_N_4_	MoS_2_ microstructures decorated on g‐C_3_N_4_ surface	260	63	10	1000 cycles	0.5 m H_2_SO_4_	[333]
MoS_2_@Mo‐S‐C_3_N_4_	2D MoS_2_ grown on 2D Mo‐S‐C_3_N_4_ composite	193	65	10	24 h	0.5 m H_2_SO_4_	[371] [372]
		290	146	10	24 h	1.0 m KOH	
CN‐MS/Acc	g‐C_3_N_4_ nanosheets/quantum dots modified by MoS_2_ nanostructures and deposited on activated carbon cloth	280	88	2	1000 cycles	0.5 m H_2_SO_4_	[372]
UH‐g‐C_3_N_4_	Uniform hexagonal g‐C_3_N_4_ nanotubes and Pt deposition	670	112.3	−	4 h	0.5 m H_2_SO_4_	[373]
UH‐g‐C_3_N_4_+ 1% Pt		450	85.3	−	4 h	0.5 m H_2_SO_4_	
g‐C_3_N_4_(90)/CN300/CoP3	In‐situ assembly of g‐C_3_N_4_ and ZIF‐Co, followed by uniform distribution of CoP	221	115	10	7 h	0.5 m H_2_SO_4_	[350]
MoS_2_‐C_3_N_4_‐60	Coupling of S‐doped C_3_N_4_ on MoS_2_	173	53	10	5000 cycles	0.5 m H_2_SO_4_	[374]
4.2 Rh‐CN	Interfacial engineering of Rh particles on carbon nitride	13	26	10	12 h	0.5 m H_2_SO_4_	[375]
		18.7		20			
		46	42	10		1.0 m KOH	
		75		20			
MX/CN/RGO	3D interweaved Ti_3_C_2_T*_x_* MXene/g‐C_3_N_4_ nanosheets/reduced graphene oxide nanoarchitecture	148	76	10	5.5 h	0.5 m H_2_SO_4_	[244]
Ru/g‐C_3_N_4_‐2	Thermal annealing of Ru nanoparticles on g‐C_3_N_4_ nanosheets.	27	22	10	20 h	0.5 m H_2_SO_4_	[254]
SCN‐MPC	S‐doped g‐C_3_N_4_ incorporated into mesoporous carbon	145	51	10	5000 cycles	0.5 m H_2_SO_4_	[175]
PtCNP_2_	P‐doping and Pt‐dispersion on g‐C_3_N_4_	22	31.2	10	−	0.5 m H_2_SO_4_	[184]
g‐C_3_N_4_@S‐Se‐pGr	g‐C_3_N_4_ coupled with S and Se co‐doped porous graphene	300	86	10	1000 cycles	0.5 m H_2_SO_4_	[376]
Au‐aerogel‐CN*_x_*	Formation of Au‐aerogel on carbon nitride nanosheets.	185	53	10	∼7 h	0.5 m H_2_SO_4_	[252]
CoP/g‐C_3_N_4_	CoP immobilized on porous g‐C_3_N_4_ nanosheets	157	66	8.5	12 h	0.5 m H_2_SO_4_	[351]
MoS_2_‐Ni‐g‐C_3_N_4_‐1	MoS_2_ decorated on Ni‐doped g‐C_3_N_4_	168	65.2	10	10 h	0.5 m H_2_SO_4_	[377]
		190	63.2	10		1.0 m KOH	
MoS_2_‐C_3_N_4_	MoS_2_ vertically anchored on C_3_N_4_ substrate	158	52	10	1000 cycles	0.5 m H_2_SO_4_	[334]
FN‐H	Fe_2_O_3_ and NiO co‐loading on crystalline g‐C_3_N_4_ nanosheets.	672	74	10	−	0.5 m H_2_SO_4_	[378]
CNF‐G	Carbon nitride frameworks padded with graphene	149	116	−	5000 cycles	0.5 m H_2_SO_4_	[379] [359]
		319	160	−	−	1.0 m KOH	
2U‐CN	Nitrogen vacancies introduced in g‐C_3_N_4_	423	18.4	10	0.6 h	Neutral media (pH 7)	[234]
C_3_N_4_@MoN	Heterostructure composed of 2D MoN and 2D g‐C_3_N_4_	110	57.8	10	1000 cycles	1.0 m KOH	[380]
C_3_N_4_@NG	Coupling between g‐C_3_N_4_ and N‐doped graphene	∼240	51.5	10	1000 cycles	0.5 m H_2_SO_4_	[246]
MoS_2_/g‐C_3_N_4_	Ultrathin Van der Waals layers consisting of MoS_2_ and g‐C_3_N_4_	∼140	45	10	1000 cycles	0.5 m H_2_SO_4_	[381]
CN0.3/BG	g‐C_3_N_4_ hybridized with B‐doped graphene	260	90	10	10 h	0.5 m H_2_SO_4_	[382]
MT‐CN‐80	C_3_N_4_ decorated onto Ti_4_O_7_ anchored MoS_2_ composite	300	54	50	33 h	0.5 m H_2_SO_4_	[383]
Pt‐NTiCN‐1.2	Nanoporous Ti‐carbon nitride loaded with Pt	27	71	10	20 h	0.5 m H_2_SO_4_	[379]
RuSe_2_/g‐C_3_N_4_	Ultrafine RuSe_2_ nanoparticles as cocatalyst for g‐C_3_N_4_	95	74.8	`	16 h	1.0 m KOH	[359]
MoSe_2_/g‐C_3_N_4_	MoSe_2_ nanosheets on superior thin C‐doped g‐C_3_N_4_ nanosheets	285	54.1	10	10 h	1.0 m KOH	[176]
Co@N‐CNT@g‐C_3_N_4_	Immobilization of Co nanoparticles into N‐doped CNTs on g‐C_3_N_4_	61	134	10	1000 cycles	1.0 m KOH	[384]
S‐g‐C_3_N_4_ /MoS_2_‐0.8	Heterojunction with MoS_2_ nanosheets grown in S‐doped g‐C_3_N_4_	267	77.2	10	1000 cycles	0.5 m H_2_SO_4_	[385]
CoS_2_/FeS_2_/ CN	Fe co‐doped with CoS_2_ ultrathin nanosheets supported on g‐C_3_N_4_	76.5	44.9	10	24 h	1.0 m KOH	[338]
Au@SrTiO_3_/g‐C_3_N_4_ PTNCs	Surface oxygen vacancy and Ti^3+^ defects in perovskite‐based ternary nanocomposites (PTNCs) decorated with Au@SrTiO_3_/g‐C_3_N_4_	82	45.36	10	10 h	0.5 m H_2_SO_4_	[320]
CNTS‐gCN (40)	Copper nickel tin sulfide decorated graphitic carbon nitride	221	86	10	1000 cycles	0.5 m H_2_SO_4_	[386]
**Electrocatalytic OER**							
gMesoCN	Nanoconfined‐mediated mesoporous carbon nitrite using SBA15 silica template	1710	62	10	24 h	0.1 m KOH	[217]
		1606	52.4	10	24 h	1.0 m KOH	
Co_3_O_4_/P‐CN	Co_3_O_4_ nanocrystals supported on P‐doped g‐C_3_N_4_	320	66.8	10	∼3 h	1.0 m KOH	[185]
Co‐M90	Co doping on 3D porous g‐C_3_N_4_ synthesized via NaCl‐assisted ball‐milling method	320	64.2	10	16 h	0.1 m KOH	[216]
Co‐C_3_N_4_/C	Co doping on in‐plane conjugated g‐C_3_N_4_ sheets with crystalline carbon	1650	53	10	3000 cycles	0.1 m KOH	[220]
NiO/CN‐2:1	NiO nanocrystals deposited on polymeric g‐C_3_N_4_	261	58.92	10	16 h	1.0 m KOH	[299]
		287	58.92	30	16 h	1.0 m KOH	
		356	58.92	100	16 h	1.0 m KOH	
		580	131.6	1	6 h	1.0 m PBS	
		666	131.6	30	6 h	1.0 m PBS	
40‐IG (IrO_2_/GCN)	IrO_2_ nanoparticles anchored on g‐C_3_N_4_ sheets	278	57	10	4 h	0.5 m H_2_SO_4_	[301]
g‐C_3_N_4_ NS‐CNT	Low temperature self‐assembly of g‐C_3_N_4_ nanosheets and CNTs	1600	83	10	10 h	0.1 m KOH	[387]
Ni‐Mn‐LDH/g‐C_3_N_4_	Coupling of Ni‐Mn‐LDH with layered polymeric g‐C_3_N_4_	316	65	10	12 h	1.0 m KOH	[388]
NiSe_2_/g‐C_3_N_4_	NiSe_2_ nanoparticles supported on multi‐layered g‐C_3_N_4_	290	143	40	10 h	1.0 m KOH	[389]
Ni‐CN‐200	Ni‐based species molecularly dispersed on g‐C_3_N_4_	1670	60	1	∼1.4 h	1.0 m KOH	[390]
g‐CN1.5	Template‐free synthesis of g‐C_3_N_4_ nanorods	316	125	10	24 h	1.0 m KOH	[391]
Co_1_Al_2_(OH)*_m_*/g‐CN*_x_*	2D Co_1_Al_2_(OH)*_m_* nanosheet dispersed on g‐C_3_N_4_	320	36	10	10 h	1.0 m KOH	[392]
g‐C_3_N_4_ /graphene	Ultrathin g‐C_3_N_4_ and graphene composite	539	68.5	10	1000 cycles	0.1 m KOH	[393]
NR‐Ni_3_N/GCNs	Ni_3_N particles constructed on N‐defective g‐C_3_N_4_, stimulation Ni‐rich sites	∼290	∼70	10	36 h	1.0 m KOH	[209]
S‐modified g‐CN*_x_*	Hydrothermal treatment of melamine nanogeodes and thermal annealing with S	290	120	10	18 h	1.0 m KOH	[394]
TCCN	Homogeneous assembly of Ti_3_C_2_ and g‐C_3_N_4_ nanosheets	1440	74.6	10	10 h	0.1 m KOH	[342]
Co(OH)_2_@g‐C_3_N_4_	Co(OH)_2_ nanowires coated by g‐C_3_N_4_ layers	320	59	10	10 h	1.0 m KOH	[360]
NiFeCr‐LDGs/g‐C_3_N_4_	Hybridization of NiFeCr‐LDHs and g‐C_3_N_4_.	223	89	10	∼2.67 h	1.0 m KOH	[395]
Co‐C_3_N_4_/CNT	Molecular‐level g‐C_3_N_4_ coordinated Co atom supported on CNTs	1610	68.4	10	3000 cycles	1.0 m KOH	[258]
MnO_2_@mpg‐C_3_N_4_	1D MnO_2_ nanowires assembled on mesoporous g‐C_3_N_4_	480	15	1	16 h	0.07 m PBS	[308]
CoNiSSe‐g‐C_3_N_4_	Bimetallic CoNiSSe grown on g‐C_3_N_4_	282	59	10	30 h	1.0 m KOH	[396]
NiFe@g‐C_3_N_4_/CNT	Ni and Fe dual metal atom doped g‐C_3_N_4_ supported on CNTs	326	67	10	16 h	1.0 m KOH	[206]
B‐doped g‐C_3_N_4_	B‐doped g‐C_3_N_4_ synthesized using H_3_BO_3_ and urea	1800	50.6	11.7	4 h	0.1 m KOH	[186]
CoFe_2_O_4_/g‐CN	CoFe_2_O_4_ supported on g‐CN nanosheet on nickel foam	200	39	10	25 h	1.0 m KOH	[397]
TCN (2:1:1)	g‐C3N4 loaded Co3O4 nanoparticles and then anchoring onto 2D Ti_3_C_2_Tx MXene	247	150.77	10	24 h	1.0 m KOH	[398]
MnO_2_/g‐CN	PE‐CVD of MnO_2_ nanoarchitectures on porous Ni scaffolds, anchoring of controllable g‐CN EPD process	430	70	10	35 h	0.1 m KOH	[399]
AR8_Ni_1_Fe_0.2__A	Ni and Fe‐nanosites onto the oxygen‐rich carbon nitrides (CNO)	351	64	−	20 h	0.1 m KOH	[400]

## 4 Bifunctional C*_x_* N*_y_* Based Electrocatalysts for OWS

Thermodynamically, electrocatalytic OWS to produce H_2_ and O_2_ is an unfavorable uphill reaction which requires Gibbs free energy, ∆*G*° of 237 kJ mol^−1^ [401]. Yet, the actual energy requirement of OWS exceeds that of theoretical value due to the charge resistances originate internally within the electrocatalyst and externally across the electrochemical circuit. In pursuance of highly efficient bifunctional OWS electrocatalysts, we hereby propose three fundamental criteria that must be fulfilled: (1) electrocatalysts serve as both cathode (HER) and anode (OER) concurrently during a reaction; (2) electrocatalysts provide low onset potential for both half‐reactions in order to achieve a closer value to that of 1.23 V; (3) electrocatalysts perform well under wide range of pH conditions. On account of the scarce number of researches performed on electrocatalytic OWS using C*_x_* N*_y_*, this section will discuss the works based on their intrinsic and extrinsic modifications of the OWS electrocatalysts. The intrinsic modifications comprise of TM doping, low atomic coordination, and strain engineering while extrinsic modifications involve the formation of unique 3D structures and hybridization.

### 4.1 Intrinsic Modifications

**FIGURE 22:**
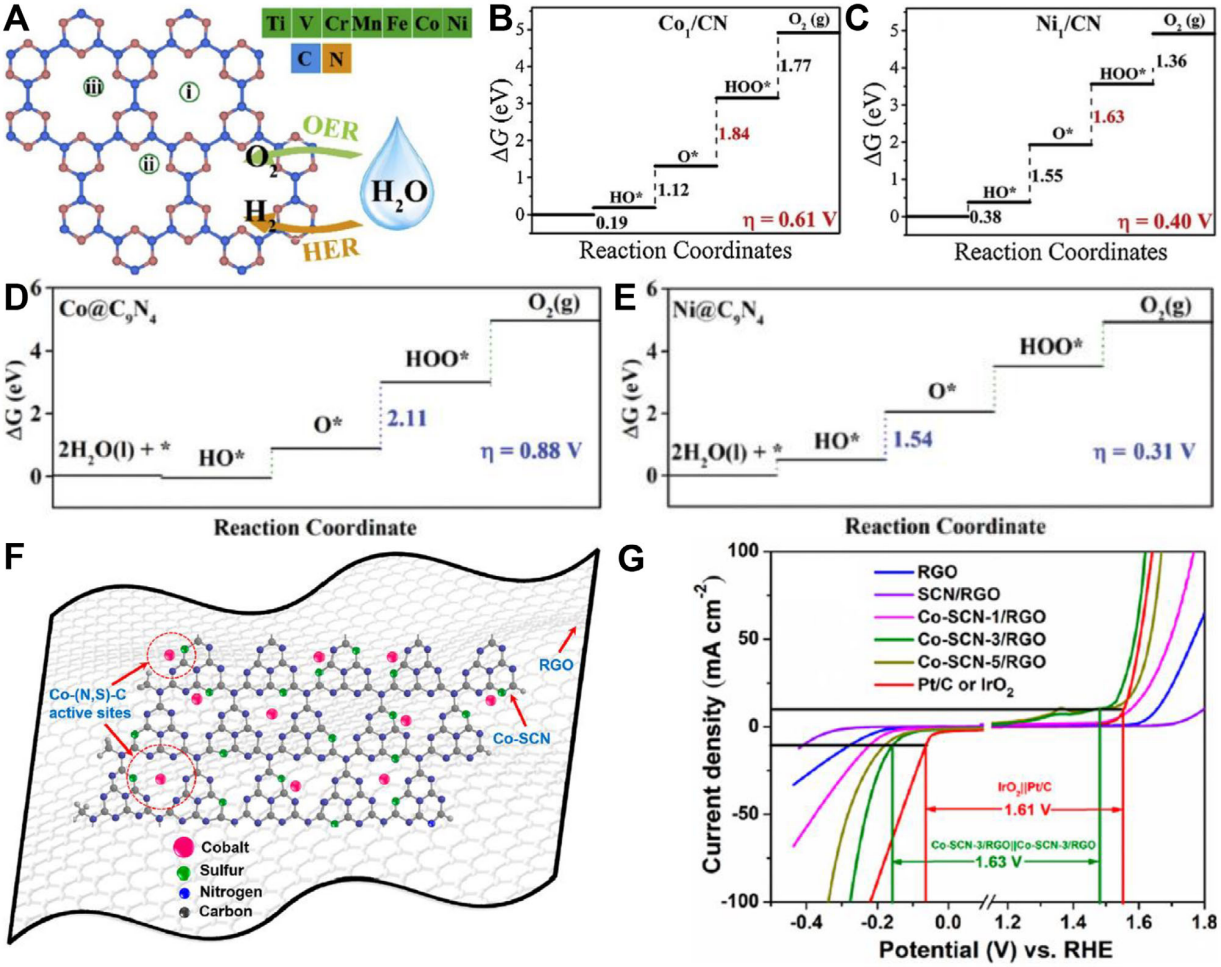
(A) Optimized structure of holey g‐CN and the possible TM doping sites: (i) located at the center of the hole, (ii) bonded to two N atoms, and (iii) bonded to three N atoms. OER free energy diagrams for (B) Co_1_/CN, (C) Ni_1_/CN. Reproduced with permission [95]. Copyright 2020, Elsevier Inc. (D) Co@C_9_N_4_, and (E) Ni@C_9_N_4_. Reproduced with permission [99]. Copyright 2020, Elsevier Inc. (F) Schematic diagram describing the possible chemical structure of Co‐SCN/RGO electrocatalyst. (G) HER and OER polarization curves of Co‐SCN‐3/RGO and other related electrocatalysts during OWS in 1.0 m KOH media. Reproduced with permission [409]. Copyright 2019, American Chemical Society.

To modulate g‐C_3_N_4_ intrinsically, Lv et al. documented a computational study involving single, double, and triple TM (Ti, V, Cr, Mn, Fe, Co, Ni) atoms doping on defective graphitic carbon nitride (g‐CN) for bifunctional HER and OER (Figure 22A). The motive behind this work was to incorporate the metallic properties of the TM atoms into the holey g‐CN framework by modulating the intrinsic charge transfer within the TM/g‐CN electrocatalysts for OWS. Thermodynamic stabilities of these electrocatalysts were deduced by calculating the difference between binding energies (*E*_b_) and cohesive energies (*E*_coh_). Since the resultant ∆*E*_b_ of single and double TM atoms doped g‐CN were all negative in value, the TM atoms were energetically more favorable to disperse uniformly on g‐CN; conversely, the addition of the third TM atom resulted in lowering the *E*_coh_ of the TM trimer and generating positive ∆*E*_b_ values, which was an indication of unwanted metal clusters formation [402, 403]. In terms of electronic properties, the Bader charge analysis demonstrated a considerable amount of electrons transfer (0.56–1.42 |e|) from the TM atoms to g‐CN framework demonstrating great potential in facilitating the OER PCET steps in OWS. In contrary to unifunctional electrocatalysis of either HER or OER, bifunctional activities require the investigation into both of these reactions. An acidic condition (pH 0) was assumed in this DFT calculation and HER performance was described via the reaction's ∆*G*(H_ads_) while that of OER was related with free energy diagrams of the PCET steps. The ∆*G*(H_ads_) closest to 0 eV was recorded by V_2_/g‐CN double TM atom electrocatalyst with −0.01 eV, closely followed by Cr_1_/g‐CN (0.02 eV), Fe_1_/g‐CN (−0.02 eV), Co_1_/g‐CN (−0.15 eV), Ni_1_/g‐CN (−0.12 eV), and Ni_2_/g‐CN (−0.05 eV). Among these splendid HER electrocatalysts with |∆*G*(H_ads_)| < 0.2 eV, Co_1_/g‐CN (Figure 22B) and Ni_1_/g‐CN (Figure 22C) recorded the lowest OER simulated overpotential ($\eta_{\mathrm{sim}}^{\mathrm{OER}}$) of 0.61 V and 0.4 V, respectively, and thus acknowledged as the two most preferred electrocatalyst configurations [95]. In another unique DFT work published by Zhou and co‐workers, C_9_N_4_ monolayer was simulated for the doping of TM (Mn, Fe, Co, Ni, Cu, Ru, Rh, Pd, Ir, and Pt) single atoms for OWS. In HER, Co@C_9_N_4_ exhibited the lowest ∆*G*(H_ads_) and highest exchange current among its peers. In this context, the charge transfer initiated by the Co dopant enabled Co@C_9_N_4_ to have multiple active sites namely Co and N atoms that are close to the optimal value zero. Similar selection methods with the previous work was employed to identify the best performing OER electrocatalysts [95]. As depicted in Figure 22D,E, a similar trend can be identified when comparing to Figure 22B,C where Ni‐doped electrocatalyst surpassed Co‐doped electrocatalyst on both occasions regardless of the type of carbon nitride material. In addition, Ni@C_9_N_4_ also had the lowest $\eta_{\mathrm{sim}}^{\mathrm{OER}}$ among the rest at 0.31 V [99]. In conclusion, computational studies in electrocatalytic OWS works best in screening potential bifunctional electrocatalysts, even so, the data simulated does not represent the OWS as a whole because only $\eta_{\mathrm{sim}}^{\mathrm{OER}}$ is set as a selection parameter while computational HER relies heavily on the ∆*G*(H_ads_), which means the half‐reactions of OWS are computed separately and individually. In actual circumstance, both HER and OER are experimented simultaneously to obtain the most accurate information of the whole electrocatalytic system, and hence, computational studies on OWS involving both HER and OER concurrently are highly sought after and revolutionary.

In general, elemental phosphorus (P) exists in several allotropes mainly white phosphorus (WP), violet phosphorus (VP), black phosphorus (BP), and red phosphorus (RP). Amongst, WP is the most active allotrope with the highest toxicity; VP suffers from limited information since its unconvincing experimental data [404, 405]; RP is less toxic than WP but it is deemed not suitable for electrocatalytic applications due to its extremely low electronic conductivity, unless forming heterostructure with BP [406, 407]; finally, BP is considered to be non‐toxic, thermodynamically stable, and possesses noteworthy electrical conductivities [408]. As such, BP has been widely applied in energy storage devices, electrocatalytic, and photocatalytic water splitting [407]. In light of this, Shiravani et al. prepared a black‐red phosphorus doped graphitic carbon nitride (BRP‐g‐C_3_N_4_) via facile one‐step thermal treatment. The synergistic effect for this composite arose from the intrinsic modulation of g‐C_3_N_4_’s electrochemical ability by the catalytic effect of the BRP dopants. In this work, BRP‐g‐C_3_N_4_ was implemented as a modifier on the carbon paste electrodes (CPE), the capabilities of BRP‐g‐C_3_N_4_ as an OWS electrocatalyst was investigated from there. BRP‐g‐C_3_N_4_‐CPE was tested in 0.5 m H_2_SO_4_, 0.1 and 1.0 m KOH solutions and the HER/OER's $\eta_{10}$ recorded were 524 (HER), 143/490, and 115/430 mV, respectively. Intriguingly, the electrocatalyst elucidated better performance at alkaline condition even for HER, which deviated from an established pattern where HER requires acidic conditions to perform well. The reason behind this achievement was partly thanks to the BRP heterostructured dopant that amplified the charge transfer within the g‐C_3_N_4_ framework, which was addressed evidently in the EIS measurements. The *R*_ct_ values of bare CPE, RP‐CPE, BRP‐g‐C_3_N_4_‐CPE were 1827, 1141, and 112 Ω, accordingly showing above tenfold of the enhancement of electrical conductivity in R_ct_ terms, which was attainable with the heterostructure formation between BP and RP as well as doping on the g‐C_3_N_4_ structure [69]. In short, metal‐free P‐based dopants or electrocatalysts are proven to be prospective alternatives of expensive noble metal catalysts in OWS given their exceptional unifunctional and bifunctional electrocatalytic behavior.

Meanwhile, the combination of P‐block element doping and TM atom coordination was realized by Jo et al. while creating a highly efficient OWS bifunctional electrocatalyst. In this research, they had designed a cobalt‐coordinated sulfur‐doped graphitic carbon nitride supported on reduced graphene oxide (Co‐SCN/RGO) for electrocatalytic OWS in 1.0 m KOH solution (Figure 22F) [409]. Among different composition (Co‐SCN‐*x*/RGO, where *x* = 1, 3, 5 wt%) of Co, Co‐SCN‐3/RGO displayed the most optimal HER/OER activities in alkaline condition. The HER/OER $\eta_{10}$ of Co‐SCN‐1/RGO, Co‐SCN‐3/RGO, and Co‐SCN‐5/RGO were 220/360, 150/250, 170/310 mV, respectively while the Tafel slope values were 118/148, 94/96, 114/126 mV dec^−1^. The origin of Co‐SCN/RGO's electrocatalytic abilities resided in its unique metal‐(N,S)‐C active sites and the lack Co contents in Co‐SCN‐1/RGO was a clear indication of insufficient Co─(N,S)─C active sites for HER and OER. In the case of Co─SCN‐5/RGO, excessive Co ions dispersed in the SCN matrix also hindered the propagation of the reaction by forming strong chemical bonds with intermediate products released during HER and OER, which consequently resulted in the decrease of H_2_ and O_2_ production. As depicted in Figure 22G, all three Co─SCN/RGO electrocatalysts were more superior than the pristine RGO and SCN/RGO electrocatalysts. On one hand, the optimum amount of Co species (3 wt%) in the Co─SCN‐3/RGO led to their effective coordination with the tri‐*s*‐triazine moieties of SCN and created the highly regarded Co─(N,S)─C active sites. On the other hand, the incorporation of S dopants boosted the adsorption and desorption energies for the facile production of H_2_, leading to more efficient electrocatalytic kinetics. Given the remarkable electrocatalytic activities of Co─SCN‐3/RGO toward both HER and OER, it obtained an overall cell potential (*E*_cell_) of 1.63 V under the cell configurations of Co─SCN‐3/RGO‖Co─SCN‐3/RGO, which was comparable to the benchmark OWS catalytic system of IrO_2_‖Pt/C at 1.61 V. Overall, Co─SCN‐3/RGO can be regarded as an electrocatalyst that operates in a class of its own with special Co─(N,S)─C active sites that perform closely to the state‐of‐the‐art noble metal electrocatalysts in OWS. Also, the action of P‐block element doping and TM coordination complements each other splendidly as observed from this research, which could inspire researchers to combine different modifications within a single catalyst design.

Strain engineering has been proven to be an effective strategy for tuning the electronic interaction between TM atoms and its substrate [410]. As discussed previously, strain engineering of g‐C_3_N_4_ electrocatalysts via non‐metal heteroatoms doping had successfully raised the electrocatalytic activities of HER [189]. Inspired from the aforementioned work, Xu et al. investigated the effects of intrinsic tensile strain on C_9_N_4_ framework before and after the doping of TM atoms through computational method. Prior to TM doping, the HER activity was represented by ∆*G*(H_ads_) at different hydrogen coverages assuming there were three equivalent N‐binding sites in each C_9_N_4_ supercell (*θ* = *n*/3, where *n* = 1, 2, 3) for both nine‐ and twelve‐membered rings of C_9_N_4_ atomic lattice. After tensile strain (up to 6%) was induced, ∆*G*(H_ads_) of all samples experienced a descending trend with every increment of strain by 1%, even the HER performance of the worst 2/3 (tmr) coverage was significantly improved to reach a ∆*G*(H_ads_) close to 0 eV. TM atoms (Co, Ni, Rh, Pd) were only loaded into the C_9_N_4_‐tmr as the C_9_N_4_‐nmr was too small for the doping of these atoms. Among these samples, ∆*G*(H_ads_) of Rh@CN (−0.03 eV) marked the lowest in comparison to Pd@CN (0.16 eV), Cp@CN (0.39 eV), and Ni@CN (0.85 eV), which was then subjected to the strain testing. By introducing small tensile strain of 0.5, 1.0, 1.5, 2.0% to Rh@CN, the ∆*G*(H_ads_) generated were −0.01, 0.01, 0.03, 0.05 eV, respectively signifying that only a miniscule amount strain (0.5 and 1.0%) was necessary to enhance the HER and any stretching beyond the optimum point led to the structural distortion of the C_9_N_4_ lattice framework, which dampened the electrocatalytic process. Considering the potential of C_9_N_4_ as a bifunctional electrocatalyst, the authors also calculated the $\eta_{\mathrm{sim}}^{\mathrm{OER}}$ of Rh@CN and benchmark IrO_2_ to be 0.58 V and 0.56 V, respectively. This demonstrated that Rh@CN's capability in OER was comparable to that of state‐of‐the‐art IrO_2_ electrocatalyst, which was considered as a highly efficient OWS bifunctional electrocatalysts [98]. In conclusion, the authors emphasized that the biggest challenge in realizing this rewarding effort is the complexity to accurately induce such a small strain upon the structure of electrocatalysts in actual practice.

**FIGURE 23:**
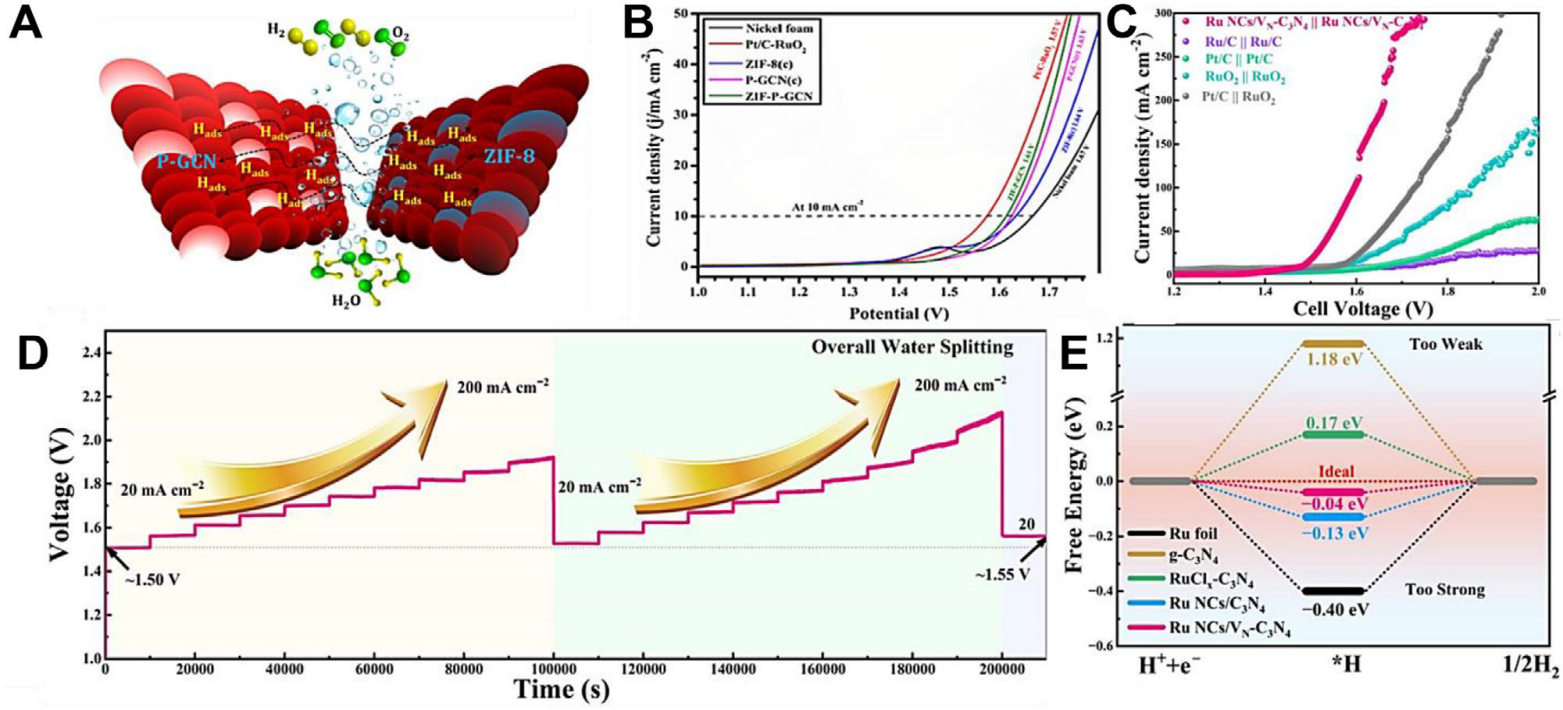
(A) Schematic illustration of the electrocatalytic water‐splitting mechanism using ZIF‐P‐GCN. (B) Electrochemical measurements of overall water splitting reactions of Pt/C, ZIF‐8(c), P‐GCN(c), and ZIF‐P‐GCN in 3 m KOH studied at 2 mV/s. Reproduced with permission. [414] Copyright 2024, Elsevier Inc. (C) LSV curves of the two‐electrode electrolyzers. (D) Multi‐current stability test starting at 20 mA cm^−2^ and ending at 200 mA cm^−2^ with an increment of 20 mA cm^−2^ every 10,000 s. (E) Calculated hydrogen adsorption free energy diagram. Reproduced with permission [416]. Copyright 2023, The Royal Society of Chemistry.

Metal‐organic frameworks (MOFs) are versatile materials characterized by their tunable porosity, high surface area, well‐defined nanometer‐scale cavities, and diverse morphologies, making them ideal candidates as catalyst precursors for electrocatalytic reactions. They have been utilized as effective sacrificial templates or starting materials to construct intricate nanoarchitectures, thereby enhancing charge transport [411, 412, 413]. Nair et al. developed a bifunctional 2D–2D electrocatalyst (ZIF‐P‐GCN nanosheets) by interacting Zn(II)‐MOFs nanosheets (ZIF‐8) with phosphorus‐doped graphitic carbon nitride (P‐GCN) nanosheets to achieve efficient overall water splitting in alkaline media (Figure 23A). XPS analysis shows that the substitution of the carbon atom in the triazine ring with a phosphorus atom led to the formation of P─N bonds. The successful incorporation of phosphorus into the GCN framework, along with the presence of P─O bonds, was verified by the peak observed at 135.05 eV. The ZIF‐P‐GCN composite demonstrated remarkable performance for OER and HER [414]. For OER, it achieved the lowest overpotential of 290 mV at 10 mA cm^−2^, and the highest TOF of 4.32 s^−1^ compared to GCN and P‐GCN(c). The Tafel slope was significantly reduced to 30.26 mV dec^−1^ for the ZIF‐P‐GCN composite, compared to 93.06 mV dec^−1^ for GCN and 61.84 mV dec^−1^ for P‐GCN(c). For HER, the ZIF‐P‐GCN composite exhibited the lowest overpotential of 200 mV. The TOF value reached 39.16 s^−1^, significantly higher than GCN and P‐GCN(c) and the Tafel slope was reduced to 46.53 mV dec^−1^. The ZIF‐P‐GCN//ZIF‐P‐GCN electrode system facilitates overall water splitting, achieving a current density of 10 mA cm^−2^ at 1.61 V, comparable to the benchmark Pt/C//RuO_2_ system (1.57 V) and outperforming other synthesized catalysts (Figure 23B). This structural modification introduced new active sites and enhanced the electrical stability of the material, facilitating efficient charge transfer during electrocatalytic reactions. These advancements underline the critical role of phosphorus doping in optimizing the electrochemical properties of g‐C_3_N_4_‐based electrocatalysts. The creation of nitrogen vacancies (VN) effectively modulates the electronic structure and charge transfer in g‐C_3_N_4_ [415]. Zhao et al. demonstrated this by encapsulating Ru nanoclusters (NCs) into g‐C_3_N_4_ rich in VN [416]. XPS and N 1s spectra confirmed VN formation through N atom removal facilitated by Ru interaction. EPR measurements revealed structural defects and strong Ru–C_3_N_4_ electron interactions, leading to electron deficiency in the carbon framework UV–vis spectra show the increased π‐electron delocalization and improved conductivity due to VN incorporation [417, 418]. Ru NCs/VN‐C_3_N_4_ exhibits outstanding HER activity with an ultra‐low overpotential of 8 mV and a Tafel slope of 24 mV dec^−1^, surpassing benchmark Pt/C (35 mV dec^−1^) and following the Volmer–Tafel mechanism. Ru NCs/VN‐C_3_N_4_ also delivers exceptional OER performance with a low overpotential of 200 mV at 10 mA cm^−2^, and a low Tafel slope of 60 mV dec^−1^, indicating fast reaction kinetics, and the highest TOF of 0.32 s^−1^ at 300 mV, 18 times higher than Ru/C. In the OWS reaction, the symmetric electrolyzer with Ru NCs/VN‐C_3_N_4_ electrodes achieves 10 mA cm^−2^ at a low cell voltage of 1.488 V, surpassing RuO_2_‖RuO_2_, Pt/C‖Pt/C, and Ru/C‖Ru/C (Figure 23C). Multistep chronopotentiometry (20–200 mA cm^−2^) demonstrates rapid potential responses and stable values over 10,000 s at each step. After two cycles (∼55.55 h), the potential returns to 1.55 V at 20 mA cm^−2^, indicating minimal potential loss and robust performance (Figure 23D). The free energy of hydrogen adsorption (Δ*G*_h_) for Ru NCs/VN‐C_3_N_4_ is −0.04 eV (Figure 23E) [419]. For the OER, the potential‐determining step (PDS) of *OOH formation has an overpotential of 1.16 V, outperforming other Ru‐based catalysts [420, 421]. VN incorporation optimizes the d‐band center, enhances electron transfer, reduces bonding strength, and shortens the Ru–substrate bond, leading to superior stability and reactivity, as confirmed by DOS, COHP, and charge density analyses. This research provides a new strategy for developing highly efficient bifunctional electrocatalysts, and displaying the prospect of commercial applications in water splitting.

In conclusion, intrinsic modification of carbon nitride‐based electrocatalysts are described. Intrinsic modification focuses on the fine‐tuning of g‐C_3_N_4_ framework itself rather than combining with other species to form a hybrid. Defect engineering via heteroatom doping, strain engineering, and TM atoms coordination are proven to be immensely promising in developing OWS electrocatalysts. Moreover, an ideal OWS electrocatalyst is one that is able to excel in wide pH conditions as shown by the investigations. Although considerable progress has been made recently in modulating g‐C_3_N_4_ electrocatalysts intrinsically, the fundamental knowledge on the mechanism between electrocatalysts and the OWS process is still lacking in general.

### 4.2 Extrinsic Modifications

**FIGURE 24:**
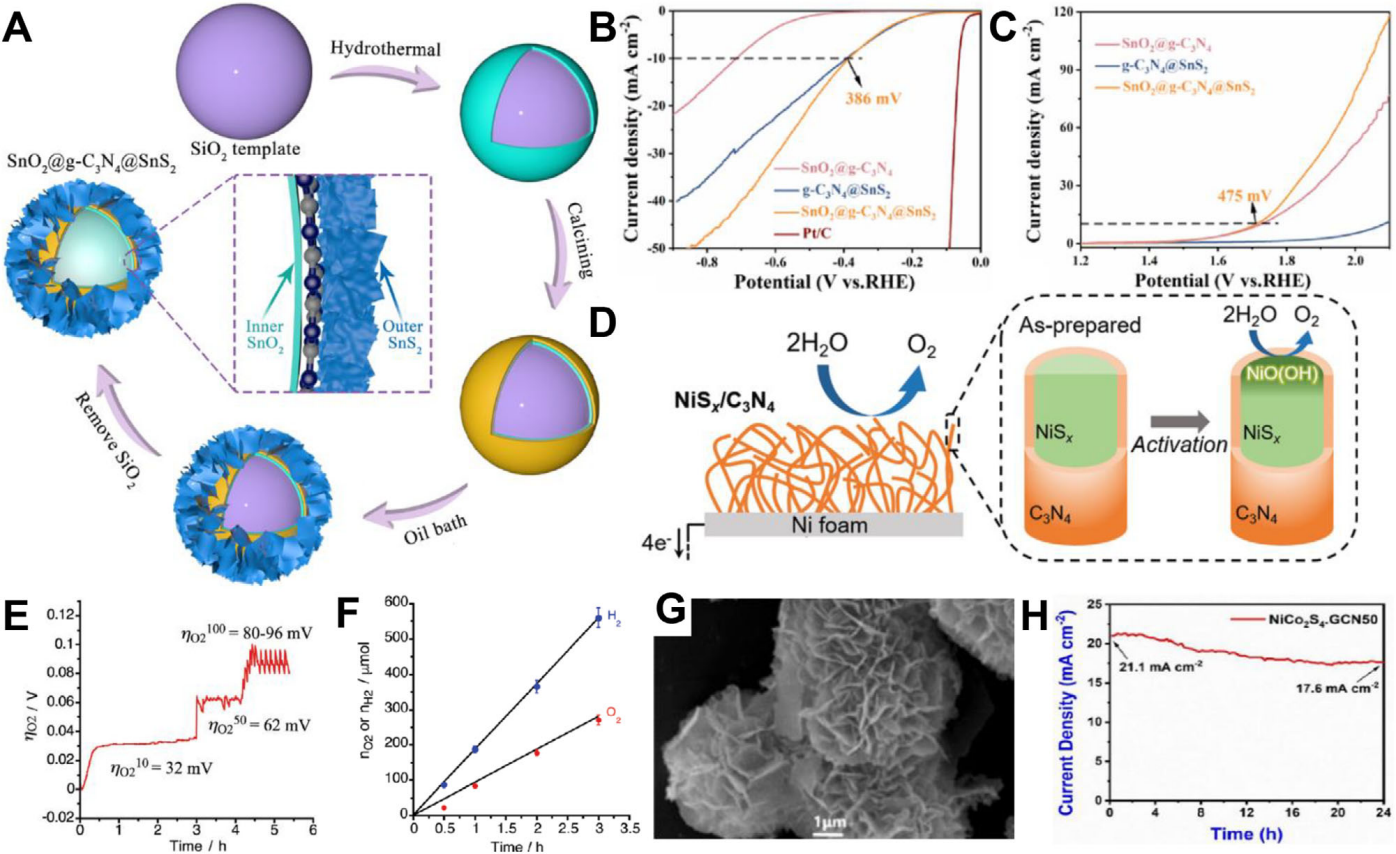
(A) Schematic illustration of the fabrication of SnO_2_@g‐C_3_N_4_@SnS_2_. LSV polarization curves of (B) HER, (C) OER. Reproduced with permission [425]. Copyright 2022, Elsevier Inc. (d) Schematic illustration of the activation process and the OER activity of the NiS*_x_*/C_3_N_4_ electrode. (E) Chronopotentiograms of OER at current densities of 10, 50, and 100 mA cm^−2^ for NiS*_x_*/C_3_N_4_. (f) $\eta_{10}$ of H_2_ (blue) and O_2_ (red) and their theoretical values (black) as a function of time. Reproduced with permission [427]. Copyright 2021, The Royal Society of Chemistry. (g) SEM image of CoNi_2_S_4_GCN50 at 1000 nm. (h) Chronoamperometric response of CoNi_2_S_4_GCN50 for 24 h. Reproduced with permission [437]. Copyright 2022, Elsevier Inc.

In order to achieve an ideal OWS electrocatalyst, catalyst design strategy combining compositional modulation and structural engineering are crucial for the selection of HER and OER active components as well as regulating the interfacial electronic structure [422]. In particular, Zhang et al. created a bifunctional electrocatalyst with SnO_2_ and SnS_2_ species separately located on the inner and outer walls of hollow g‐C_3_N_4_ denoted as SnO_2_@g‐C_3_N_4_@SnS_2_ utilizing an effective stepwise self‐assembly strategy (Figure 24A). The rationale of selecting SnO_2_ as the inner wall of the electrocatalyst was attributed to SnO_2_’s excellent corrosion resistance in strong alkaline conditions and exceptional electron transfer ability [423]. Likewise, tin‐based sulfides such as SnS_2_ are composed on S‐SN‐S atomic layers coordinated by van der Waals forces, which is distinctly suitable to serve as an outer layer because it provides abundant active sites [424]. To accurately investigate the active components, SnO_2_@g‐C_3_N_4_ and g‐C_3_N_4_@SnS_2_ were synthesized as well for comparison. As portrayed in the LSV curves, SnO_2_@g‐C_3_N_4_@SnS_2_ was almost similar to that of g‐C_3_N_4_@SnS_2_ in HER under 1.0 m KOH when the $\eta_{10}$ reached 386 mV (Figure 24B); while in stark contrast, SnO_2_@g‐C_3_N_4_@SnS_2_ and SnO_2_@g‐C_3_N_4_ revealed a similarity of $\eta_{10}$ (475 mV) in OER under similar alkaline conditions (Figure 24C). Hence, g‐C_3_N_4_@SnS_2_ and SnO_2_@g‐C_3_N_4_ were the active components for HER and OER, respectively. As the carrier (for SnS_2_) and protective layer (for SnS_2_) of this electrocatalyst, g‐C_3_N_4_ played a major role in improving the stability (Figure 24D,E) and catalytic activity of the electrocatalyst. Notably in HER, the C atoms in g‐C_3_N_4_ were able to increase the electron density around the S atoms, which incited the appearance of additional states near the Fermi level, thereby promoting the adsorption of hydrogen atoms. For OER, N atoms of g‐C_3_N_4_ with lower electronegativity than O atoms of SnO_2_ led to lesser electron transfer to the adjacent O atoms, thus reducing the Bader charge numbers during the adsorption of OH^−^ and OOH_ads_ to optimize the adsorption of OER intermediate products. In addition, these synergistic interactions between oxide‐carrier‐sulfide also produced higher values of C_dl_ and ECSA for SnO_2_@g‐C_3_N_4_@SnS_2_ with 0.247 mF cm^−2^ and 0.441 cm^2^, respectively, which proved that the sample possessed higher specific area and exposed active sites compared to SnO_2_@g‐C_3_N_4_ (0.068 mF cm^−2^ and 0.122 cm^2^) and _g_‐C_3_N_4_@SnS_2_ (0.246 mF cm^−2^ and 0.439 cm^2^). In OWS under 1.0 m KOH, SnO_2_@g‐C_3_N_4_@SnS_2_ achieved a cell potential of 1.76 V at 10 mA cm^−2^ [425]. Briefly, the extrinsic modification of g‐C_3_N_4_ nanosheets into hollow nanospheres has determined to be highly functional especially the rigid structure and relatively superior electrocatalytic activity in both OWS half‐reactions.

Essential to the OWS half‐reaction, OER is technically referred to as the bottleneck since it consists of sluggish kinetics arising from the rigid O─O double bond formation and multiple PCET steps [426], which ultimately dampens the OWS activity. In light of this, Zahran et al. succeeded in lowering the required $\eta_{10}$ drastically for OER (32 mV) to achieve an $\eta_{\mathrm{cell}}$ of 72 mV with a cell potential of 1.30 V by stuffing nickel sulfide nanowires into carbon nitride scabbards (NiS*_x_*/C_3_N_4_) via calcination procedure up to 450°C. These results were possible as a result of its activation mechanism and unique electrocatalyst‐support relationship. As illustrated in Figure 24F, OWS was initiated upon the activation of the electrocatalyst by transforming NiS*_x_* active components into Ni^III^O(OH) as the OER active sites. Then, by having NiS*_x_* formed directly from the basal nickel foam (NF) surface and covered with C_3_N_4_ outer layer, the resistance at the interface between the NiS*_x_*/C_3_N_4_ electrocatalyst and NF support was greatly minimized. Besides, nickel sulfides possess higher quality of electronic conductivity, even higher than that of nickel oxides [140]. Hence, these discriminative features led to the efficient charge transfer from the NF support to the OER active sites, which generated superior OER performance in 1.0 m KOH electrolyte solution. The as‐prepared electrocatalyst was subjected to chronopotentiometry testing at high current densities of 50 and 100 mA cm^−2^ and recorded overpotentials of $\eta_{50}$= 62 mV and $\eta_{100}$= 80–96 mV, respectively (Figure 24G), these gratifying results proved that NiS*_x_*/C_3_N_4_ was indeed suitable for high current densities applications. To further verify its potential, the stability of NiS*_x_*/C_3_N_4_ was examined at 250 mA cm^−2^ (standard technologically relevant current density). It was observed that the overpotential rose from 35 to 236 mV@10 to 250 mA cm^−2^ for the first 30 min and continue fluctuating between 236 and 53 mV until 6.5 h mark owing to the formation of O_2_ bubbles from OER, and the average overpotential for this period was approximately 146 mV. After 8.5 h, the overpotential obtained at 250 mA cm^−2^ was 269 mV, which was attributed to the partial detachment of the NiS*_x_*/C_3_N_4_ from the NF support. In terms of OWS at 10 mA cm^−2^, the amount of O_2_ and H_2_ evolved at both anode and cathode as well as the $\eta_{10}$ increased linearly with time and the FE calculated for OER and HER were close to 100% from the comparison with the theoretical coulometric slope data (Figure 24H) [427]. Overall, $\eta_{\mathrm{cell}}$ value of 72 mV (comprised of 32 mV and 40 mV from OER and HER, respectively) was considered extremely low compared with the state‐of‐the‐art OWS cells that exceeded 315 mV [428, 429]. In summary, the authors had developed a bifunctional electrocatalyst that leaned towards unprecedentedly low OER overpotential values that boasted high potentials for overcoming the OWS bottleneck. However, exfoliation of electrocatalytic materials from the support is still considered a major setback for practical applications, which requires further attention.

One of the fundamental reasons that g‐C_3_N_4_ is considered as a robust catalyst support is ascribed to the pyridinic N species that continuously induce a high positive charge towards the nearby sp^2^‐hybridized C atoms [430], which favors the adsorption of reactants and rapid charge transfers. Besides, g‐C_3_N_4_ encourages good dispersion of active components to expose more active sites and prevents aggregation [431]. Considering these merits, Jiang et al. constructed a nanocomposite with monodispersed Ir NPs on g‐C_3_N_4_ coupled N‐doped graphene (Ir/g‐C_3_N_4_/NG) via simple chemical reduction approach for OWS in 0.5 m H_2_SO_4_ medium. Ir NPs were selected as active components in this work because they exhibited a ∆*G*(H_ads_) value closer to zero than Pt. Among as‐prepared samples (g‐C_3_N_4_/NG, pure Ir, 6.2 wt% Ir/g‐C_3_N_4_, 5.7 wt% Ir/NG, and Pt/C), the 5.9 wt% Ir/g‐C_3_N_4_/NG registered the best HER performances with $\eta_{10}$ of 22 mV and, which was smaller than that of state‐of‐the‐art Pt/C electrocatalyst at 28 mV. A HER Tafel slope value of 22 mV dec^−1^ hinted that the electrocatalyst's HER kinetic was based on Volmer–Tafel mechanism and the RDS belonged to the H‐H recombination step. Similarly, 5.9 wt% Ir/g‐C_3_N_4_/NG was also the most optimum electrocatalyst in OER with $\eta_{10}$ and Tafel slope of 287 mV and 72.8 mV dec^−1^, respectively. The mass activities, which directly reflects ECSA of 5.9 wt% Ir/g‐C_3_N_4_/NG were determined to be 5.52 and 2.31 A mg_Ir_^−1^, respectively which were many times larger than that of 5.7 wt% Ir/NG (1.28 and 0.81 A mg_Ir_^−1^) and pure Ir (0.30 and 0.52 A mg_Ir_^−1^). Thus, the impressive electrocatalytic activity of the as‐synthesized electrocatalyst was contributed by the large ECSA and rapid charge transfer process. Furthermore, the coupling of g‐C_3_N_4_ with NG assisted in modulating the adsorption‐desorption behavior of the nanocomposite, which benefitted in the electrocatalytic activity and the bifunctional electrocatalyst reached an overall cell potential of 1.56 V during OWS [432]. Interestingly, 5.9 wt% Ir/g‐C_3_N_4_/NG's performance was satisfyingly good despite using an acidic solution (0.5 m H_2_SO_4_), which was highly beneficial to HER but not OER. By envisioning this bifunctional electrocatalyst in an alkaline medium, the current $\eta_{10}$ of OER (287 mV) can be further reduced.

Generally, 3D porous carbon materials present many benefits over 0D carbon dots, 1D carbon nanotubes, and 2D graphene nanosheets, especially in catalysis, which include the large surface area abundant in exposed active sites. For instance, edge‐located active sites were proven to be more active than the ones located on the plane or in bulk form of the material. Likewise, 3D nanostructures provide a multidimensional conductive network with exceptional electrical conductivity and allow easy access of ions‐containing electrolyte through the porous 3D frameworks [433, 434]. In the quest for highly efficient metal‐free OWS electrocatalysts, Peng et al. developed a metal‐free carbon nitride‐based electrocatalyst for OWS by growing C_3_N_4_ on hierarchical carbon nanotube/carbon fiber (C_3_N_4_─CNT─CF) for OER and sulfur‐doped C_3_N_4_─CNT─CF (S─C_3_N_4_─CNT─CF) for HER via in situ growth using π─π stacking. These electrocatalysts were tested in both 0.5 m H_2_SO_4_ and 1.0 m KOH electrolytes. Owing to its self‐supporting properties, both C_3_N_4_─CNT─CF and S─C_3_N_4_─CNT─CF were used directly as the working electrodes. For OER in 0.5 m H_2_SO_4_ and 1.0 m KOH, C_3_N_4_─CNT─CF had recorded the onset potentials of 1.52 and 1.35 V, respectively while the Tafel slope values were 45 and 129 mV dec^−1^, accordingly. On the other hand, the HER electrocatalyst (S─C_3_N_4_─CNT─CF) documented onset potentials of 0.15 and 0.05 mV in 0.5 m H_2_SO_4_ and 1.0 m KOH, respectively, and the Tafel slopes were 81.6 and 79 mV dec^−1^. Unexpectedly in this work, OER (C_3_N_4_─CNT─CF) preferred acidic condition while HER (S─C_3_N_4_─CNT─CF) preferred alkaline condition, which was uncommon based on previous literature. In OWS, the C_3_N_4_─CNT─CF‖S─C_3_N_4_─CNT‐CF system presented low cell potentials of 1.60 and 1.55 V in 1.0 m KOH and 0.5 m H_2_SO_4_, respectively, which suggested that the these electrocatalysts were highly suited for wide ranges of pH with superior activity in acidic conditions [435]. In short, this work proves that 3D structured electrocatalysts are highly versatile in terms of wide range of pH conditions.

In another work, Zahra et al. developed a 3D hierarchical flowers‐like bifunctional electrocatalyst by combining nickel cobalt sulfide and g‐C_3_N_4_ (CoNi_2_S_4_GCN) via facile hydrothermal method. At 50 mg of g‐C_3_N_4_, the optimized CoNi_2_S_4_GCN50 structure presented a well‐defined petal‐like pattern on the outer layer of the spherical hybrid. The petals belonged to the interconnecting layers of g‐C_3_N_4_ nanosheets and amplified the exposure of active sites by providing specific transportation channels towards the CoNi_2_S_4_ active material [436], which was validated through the BET surface area increment from 3.5 (CoNi_2_S_4_) to 11.61 m^2^ g^−1^ (CoNi_2_S_4_GCN50). The ECSA of CoNi_2_S_4_GCN50 was indeed the highest value at 961.7 as compared to its counterparts CoNi_2_S_4,_ CoNi_2_S_4_GCN30, and CoNi_2_S_4_GCN100 at 325.7, 362.2, and 145, respectively. These results suggested that low composition of g‐C_3_N_4_ in the hybrid (CoNi_2_S_4_GCN30) only had small effect on the structure which excessive addition of g‐C_3_N_4_ (CoNi_2_S_4_GCN100) backfired and caused the blockage of the CoNi_2_S_4_ active sites. Hence, the optimized CoNi_2_S_4_GCN50 obtained an OWS cell potential of 1.58 V at 10 mA cm^−2^, which was much lower than the state‐of‐the‐art IrO_2_ and Pt/C electrocatalysts at 1.81 V. The exceptional results acquired stemmed from the electrocatalyst's superior performance in the OWS half reactions. Obviously, the OWS‐limiting reaction was associated with the sluggish OER. Therefore, HER and OER was tested under 1.0 m KOH solution and the overpotentials achieved by CoNi_2_S_4_GCN50 were 160 mV at 10 mA cm^−2^ and 310 mV at 30 mA cm^−2^, respectively while the Tafel slopes were 90.76 and 49.86 mV dec^−1^, accordingly. The electrocatalytic activities were mainly contributed by the synergistic effects between CoNi_2_S_4_ and g‐C_3_N_4_, the addition of g‐C_3_N_4_ lowered the binding energy of CoNi_2_S_4_ to the reacting intermediates, which facilitated the HER and OER process. Additionally, the hierarchical arrangement of g‐C_3_N_4_ on the hybrid's structure enabled equilibrium coverage of intermediates on CoNi_2_S_4_ active sites and the transportation channels created were responsible for enhanced diffusion of ions. In terms of stability, CoNi_2_S_4_GCN50 was able to maintain 90% and 83% of its initial current density after 12 and 24 h, respectively of OWS process in a 1.0 m KOH solution [437]. In short, both experiments conducted on 3D‐structured g‐C_3_N_4_ electrocatalysts had shown satisfying results, it was also observed that 3D structures were considerably robust with great potential in serving as self‐standing electrodes. Table 3 summarizes the performance of C_x_N_y_ in electrocatalytic overall water spliting.

**TABLE 3:** Electrocatalytic performance of bifunctional C*_x_* N*_y_*‐based catalysts.

Catalyst	Modification	Overpotential @10 mA cm^−2^ [mV]		Tafel Slope [mV dec^−1^]		OWS cell potential @10 mA cm^−2^ [V]	Medium	Ref.
		HER	OER	HER	OER			
Co‐SCN‐3/RGO	Co atoms coordinated‐S‐doped g‐C_3_N_4_ supported on RGO.	150	250	94	96	1.63	1.0 m KOH	[409]
ZIF‐P‐GCN	Zn(II)‐MOFs nanosheets interaction with P‐doped gCN nanosheets	290	200	46.53	30.26	1.61	3.0 m KOH	[414]
Ru NCs/VN‐C_3_N_4_	Encapsulating Ru nanoclusters into g‐C_3_N_4_ rich in N‐vacancies	8	200	24	60	1.488	1.0 m KOH	[416]
SnO_2_@g‐C_3_N_4_@SnS_2_	Hollow nanosphere with SnO_2_ as inner wall and SnS_2_ as outer wall of g‐C_3_N_4_	386	475	291	253	1.76	1.0 m KOH	[425]
NiS*_x_*/C_3_N_4_	Nickel sulfide nanowires stuffed into carbon nitride scabbards	40	32	−	48	1.30	1.0 m KOH	[427]
5.9 wt% Ir/g‐C_3_N_4_/NG	Monodispersed Ir NPs on g‐C_3_N_4_ coupled N‐doped graphene nanocomposite	22	287	22	72.8	1.56	0.5 m H_2_SO_4_	[432]
C_3_N_4_─CNT─CF (OER) S‐C_3_N_4_─CNT─CF (HER)	3D hierarchical carbon network and layered carbon nitride with S doping for HER	236	348	81.6	129	1.55	0.5 m H_2_SO_4_	[435]
		131	1600	79	45	1.60	1.0 m KOH	
CoNi_2_S_4_GCN50	3D hierarchical flowers‐like CoNi_2_S_4_ and g‐C_3_N_4_ hybrid electrocatalyst	160	310@30 mA cm^−2^	90.76	49.86	1.58	1.0 m KOH	[437]
CoO*_x_*/CoN*_y_* @ CN*_z_*_,700_	A series of cobalt oxides embedded within carbon nitride matrix	265	280	84	61	1.57	1.0 m KOH	[438]
NS‐3.0	NiS‐NiS_2_ nanoparticles in situ grown on the layered porous sulfur‐doped graphitic carbon nitride	144	298@50 mA cm^−2^	96.7	108.9	1.66	1.0 m KOH	[439]
CGD	Incorporate cobalt ferrite using gCN and NGQDs	287	455	94	69	2	1.0 m KOH	[440]
3DG‐Mix	Hexagonal Cu_3_P platelets and g‐C_3_N_4_ nanoflakes constructed on 3D‐graphene network	67	255	45	40	1.54	1.0 m KOH	[94]
Ru@g‐CN*_x_*	Ru electrocatalyst embedded in graphite carbon nitride	53.2	280	33.2	49.5	1.51	1.0 m KOH	[441]
NiTe‐HfTe_2_/g‐C_3_N_4_	NiTe‐HfTe_2_ was anchored onto the ultrathin layered g‐C_3_N_4_	71	150	75	47	1.49	0.1 m KOH	[442]
Mo_0.84_Ni_0.16_‐Mo_2_C@NC	Mo‐Ni alloy and Mo_2_C nanoparticles homogeneously distribute in nitrogen‐rich carbon‐based material	151	285	112	60	1.64	1.0 m KOH	[443]
Fe_3_N@CN‐700	Iron nitride embedded graphitic carbon nitride iron nitride embedded graphitic carbon nitride	133	281	86	56	1.62	1.0 m KOH	[444]
WC/Co_3_W_3_N/Co@NC	Nitrogen‐doped porous carbon confined yolk/shell of WC/Co_3_W_3_N/C	141	280	103	79	1.61	1.0 m KOH	[445]
SmAlS_3_/g‐C_3_N	Samarium aluminum sulfide and graphitic carbon nitride composite	305	278	63	49	1.61	1.0 m KOH	[446]
MoCoPCN	Bimetallic MoCo catalyst integrated within a three‐dimensional (3D) nanoporous network of N, P‐doped carbon nitride	49.5	202	47.5	45.7	1.49	1.0 m KOH	[447]

## 5 Conclusion and Future Perspective

Electrocatalysis in OWS functions to accelerate the realization of the seventh sustainable development goal, which is ensuring access to affordable, reliable, sustainable, and modern energy for every nation via production of H_2_. The development C*_x_* N*_y_*‐based electrocatalysts has shown significant advances in recent years owing to its unique physicochemical properties and versatility in application. In this review, we have summarized recent state‐of‐the‐art C*_x_* N*_y_*‐based electrocatalysts in HER, OER, and OWS. Emphasis of this topic was put on the structure‐performance relationship of the electrocatalysts as well as bridging available computational and experimental findings. In summary, the performance of unifunctional C*_x_* N*_y_*‐based electrocatalysts in HER and OER were partly determined by pH conditions. For instance, H_2_ generation is boosted under low pH while O_2_ production is better at high pH. On the other part, modifications implemented on the pristine C*_x_* N*_y_* brings about numerous rewarding qualities such as enhancing charge transfer abilities, strengthening physicochemical and catalytic stabilities, and establishing synergistic effects that ultimately contribute towards astonishing reaction kinetics. However, the application of bifunctional C*_x_* N*_y_*‐based electrocatalysts in OWS is currently considered a new age in the fields of catalysis, materials science, and energy production. In this burgeoning field of OWS, there is no doubt that research in this field will only gain more attention in the upcoming years following the actual potential for C*_x_* N*_y_* to become the next catalytic material of choice in energy conversion devices like water electrolyzers in this context. As part of the carbon family similar to that of renowned graphene material, C*_x_* N*_y_* itself is easily synthesized, low cost, highly tunable, remarkable catalytic performance, and in some cases, multifunctional electrocatalyst. Hence, we envision and propose six main aspects that researchers should focus their efforts on.

### 5.1 Meet Real‐World Applicability and Performance Under Industrial Conditions

Enhanced long‐term stability of carbon nitride‐based catalysts under industrially relevant conditions is crucial for their large‐scale deployment in water electrolysis. While these materials demonstrate promising performance in laboratory studies, their durability and efficiency under practical operating conditions remain underexplored. Industrial OWS operates at high current densities (0.4–1 A cm^−2^), fluctuating pH, elevated pressures (0.1–0.3 MPa), and temperatures ranging from 50 to 80°C [448, 449]. To meet these demands, catalysts must exhibit sustained activity, minimal degradation, and resistance to fouling over extended periods. Recent studies have reported electrocatalyst durability of up to 4000 h in electrocatalytic OWS [450], yet further improvements are required to match the operational lifetimes needed for commercial applications. Additionally, industrial‐scale electrolyzers require high efficiency and low overpotentials (1.8–2.2 V) at high throughput, necessitating engineering advancements in C*_x_* N*_y_* to optimize active site accessibility and electron transport properties [448, 449]. Future research should focus on pilot‐scale demonstrations to bridge the gap between laboratory innovation and industrial implementation, ensuring the viability of carbon nitride‐based catalysts for large‐scale hydrogen production.

### 5.2 Synthesize Bifunctional Electrocatalysts That Perform Well Under Wide Range of pH Conditions

Generally, most non‐precious bifunctional electrocatalysts for OWS only function well in alkaline or neutral conditions as OER is the more demanding half‐reaction compared to its counterpart HER. However, there is another underlying factor that encourages researcher to investigate OWS under alkaline conditions, that is carbon‐based electrocatalysts suffered from greater risks of degradation in acidic electrolyte. Furthermore, the current commercial fuel cells and water electrolyzers operate with the utilization of nafion as a proton conducting membrane, which is also highly acidic. Therefore, it is crucial for researchers to develop bifunctional C*_x_* N*_y_* materials that tolerate wide range of pH conditions to replace the costly noble metal electrocatalysts. Also, g‐C_3_N_4_ can also be fine‐tuned to manifest commendable corrosion resistance especially in OWS using seawater as the substrate, which is another highly sought‐after direction.

### 5.3 Fabrication Limitations of Carbon Nitride in OWS

Developing cost‐effective and scalable synthetic strategies is a prerequisite for advancing the industrialization of C*_x_* N*_y_*‐based electrocatalysts. While these materials can be synthesized from various molecular precursors, their final structures are highly dependent on monomer selection and fabrication conditions. However, conventional methods such as thermal polymerization, chemical vapor deposition (CVD), and templating approaches often require precise reaction conditions, high‐purity precursors, and energy‐intensive processing, limiting their feasibility for large‐scale production. Additionally, high‐temperature synthesis typically results in low yields due to nitrogen gas evolution, which reduces the nitrogen content in the final product. The need for an inert atmosphere during pyrolysis and template‐assisted synthesis further constrains scalability, making large‐scale industrial production challenging under current methodologies. Furthermore, ensuring structural uniformity, porosity control, and high surface area in C*_x_* N*_y_* materials remains a critical challenge, as these factors directly impact catalytic performance. Addressing these fabrication limitations is essential for transitioning carbon nitride‐based catalysts from experimental research to industrial application, and without significant advancements in synthesis techniques, large‐scale adoption may remain distant.

### 5.4 Construct Self‐Supported Electrocatalysts via Additive Manufacturing Techniques

Based on some of the works discussed in this review, g‐C_3_N_4_ possesses suitable characteristics to be constructed as a self‐supported electrocatalysts by forming hybrids and unique 3D structures. The merits of self‐supported electrocatalysts reside in overcoming some of the common issues like exfoliation of catalytic active components from the catalyst support after an extended period of usage that often reduces the efficiency of the electrochemical. Without the supporting material, higher order formulation techniques can be employed to produce C*_x_* N*_y_*‐based electrocatalysts in large scale directly. Additive manufacturing also known as 3D printing is a promising technique to rapidly construct diverse 3D C*_x_* N*_y_* architectures with controllable macro‐ and microstructures. For example, Jiang and coworkers had recently developed a self‐supported 3D printed g‐C_3_N_4_/CNT arrays for photoelectrocatalytic HER via direct ink writing that proved to facilitate outstanding catalytic performance [396]. Therefore, other high‐resolution 3D printing techniques namely masked stereolithography and digital light processing can also be applied in developing bifunctional C*_x_* N*_y_*‐based electrocatalysts for OWS in the near future as the technology evolvement in the IR4.0 constant expands from the point of view of automation and machine learning.

### 5.5 Bridge Theory and Experiment to Advance Catalyst Design

Computational and experimental findings should be considered during catalyst design as both methods verify each other simultaneously, which strengthens the credibility of a finding and making it more meaningful. Next, theoretical research can be performed on extremely rare metals to obtain novel discoveries such as novel structures and chemical properties before performing the experiments. This allows the researchers to speed up their catalyst design stage when searching for new materials as well as avoiding any wastage of resources. Additionally, the extent of computational research, namely machine learning, in catalyst design has yet to reach its full potential, which possibly opens up novel experimental avenues beyond that of g‐C_3_N_4_ and other identified allotropes like C_9_N_4_ and C_2_N in electrocatalytic water splitting.

### 5.6 Involve ML to Predict Novel Discoveries

With the deepening of computational researches, ML is often used to identify the significance of proposed descriptors for an electrocatalyst. With the aid of DFT, many excellent descriptors, for example, charge transfer, binding energies, and formation energies predicted by ML have been compared with analytical experimental parameters and the utmost important parameter is usually adopted directly in the experiments while very few researchers verify the accuracy of such predictions. Related follow‐up studies on other parameters are highly encouraged to raise the credibility of ML in the field of electrocatalysis. Furthermore, researchers who aim to improve beyond the scope of electrocatalytic process are recommended to apply ML during system design.

## Conflicts of Interest

The authors declare no conflicts of interest.

## Data Availability

Data sharing not applicable no new data generated
